# Prevalence of sarcopenia in patients with chronic kidney disease: a global systematic review and meta‐analysis

**DOI:** 10.1002/jcsm.13425

**Published:** 2024-01-24

**Authors:** Marvery P. Duarte, Lucas S. Almeida, Silvia G.R. Neri, Juliana S. Oliveira, Thomas J. Wilkinson, Heitor S. Ribeiro, Ricardo M. Lima

**Affiliations:** ^1^ Faculty of Physical Education University of Brasilia Brasília Brazil; ^2^ Faculty of Health Sciences University of Brasilia Brasília Brazil; ^3^ Institute for Musculoskeletal Health The University of Sydney and Sydney Local Health District Sydney Australia; ^4^ School of Public Health, Faculty of Medicine and Health The University of Sydney Sydney Australia; ^5^ Diabetes Research Centre University of Leicester Leicester UK

**Keywords:** Cachexia, Chronic kidney disease, Dialysis, Muscle mass, Muscle strength, Physical function

## Abstract

Sarcopenia is a risk factor for adverse clinical outcomes in chronic kidney disease (CKD) patients, including mortality. Diagnosis depends on adopted consensus definition and cutoff values; thus, prevalence rates are generally heterogeneous. We conducted a systematic review and meta‐analysis to investigate the global prevalence of sarcopenia and its traits across the wide spectrum of CKD. A systematic search was conducted using databases, including MEDLINE and EMBASE, for observational studies reporting the prevalence of sarcopenia. We considered sarcopenia according to the consensus definition of the European Working Group on Sarcopenia in Older People (EWGSOP), the Asian Working Group for Sarcopenia, the Foundation for the National Institutes of Health Sarcopenia Project, and the International Working Group on Sarcopenia (IWGS). Subgroup analyses by CKD stages, consensus, and gender were performed. Pooled prevalence was obtained from random‐effect models. A total of 140 studies (42 041 patients) across 25 countries were included in this systematic review and meta‐analyses. Global prevalence of sarcopenia was 24.5% [95% confidence interval (CI): 20.9–28.3) and did not differ among stages (*P* = 0.33). Prevalence varied according to the consensus definition from 11% to 30%, with no significant difference (*P* = 0.42). Prevalence of severe sarcopenia was 21.0% (95% CI: 11.7–32.0), with higher rates for patients on dialysis (26.2%, 95% CI: 16.6–37.1) compared to non‐dialysis (3.0%, 95% CI: 0–11.1; *P* < 0.01). Sarcopenic obesity was observed in 10.8% (95% CI: 3.5–21.2). Regarding sarcopenia traits, low muscle strength was found in 43.4% (95%CI: 35.0–51.9), low muscle mass in 29.1% (95% CI: 23.9–34.5), and low physical performance in 38.6 (95% CI: 30.9–46.6) for overall CKD. Prevalence was only higher in patients on dialysis (50.0%, 95% CI: 41.7–57.4) compared to non‐dialysis (19.6%, 95% CI: 12.8–27.3; *P* < 0.01) for low muscle strength. We found a high global prevalence of sarcopenia in the wide spectrum of CKD. Low muscle strength, the primary sarcopenia trait, was found in almost half of the overall population with CKD. Patients on dialysis were more prevalent to low muscle strength and severe sarcopenia. Nephrology professionals should be aware of regularly assessing sarcopenia and its traits in patients with CKD, especially those on dialysis.

## Introduction

Sarcopenia was first described as an age‐related loss of skeletal muscle mass.^1^ Currently, sarcopenia is recognized as a musculoskeletal disease and involves not only loss of muscle mass but also the presence of other traits, namely, reduced muscle strength and/or physical performance.^2^ Chronic kidney disease (CKD) is an accelerated aging condition that may lead to progressive changes in physical function and nutritional abnormalities, predisposing CKD patients to a higher risk of developing sarcopenia, especially in end‐stage kidney disease, which has been associated with multiple adverse clinical outcomes, including mortality.^3^, ^4^

Although sarcopenia is a well‐recognized disease in the older population, CKD *per se* is considered a risk factor for musculoskeletal disorders, mainly given its associated metabolic disturbances and systemic inflammation.^5^, ^6^ Indeed, a growing body of evidence shows a positive association between CKD stages and sarcopenia prevalence.^7^ Overall, the prevalence of sarcopenia in the whole spectrum of CKD ranges from 4% to 42%.^7^ Although previous publications have reported the prevalence of sarcopenia in dialysis and kidney transplant recipients,^4^, ^8^ there is currently no robust prevalence data considering the whole spectrum of the CKD population. The concept of sarcopenia severity has also been introduced, with severe sarcopenia being defined as the presence of all sarcopenia traits (i.e., low muscle strength, low muscle mass, and low physical performance).^9^ The frequency of sarcopenia‐related traits and severe sarcopenia in patients with CKD would be valuable in clinical and research settings, but these data have yet to emerge. The coexistence of sarcopenia and excess body adiposity has been referred to as sarcopenic obesity, a condition recognized as an important health concern in different populations, including in CKD patients.^10^ The frequency of sarcopenic obesity in this population, however, has been poorly investigated.

Therefore, we conducted a comprehensive systematic review and meta‐analysis to identify the global prevalence of sarcopenia and related traits in the wide spectrum of CKD population. This is of particular interest given the impact of sarcopenia on adverse clinical outcomes in patients with CKD to provide vital information for future research and clinical practice guidelines and to set priorities regarding public health and research.

## Methods

This systematic review and meta‐analysis was conducted following the meta‐analyses and systematic reviews of observational studies and the Joanna Briggs Institute (JBI) methodological guidance for systematic reviews of observational epidemiological studies.^11^, ^12^ The protocol was previously registered at PROSPERO (CRD42020213659).

### Search strategy

We conducted a systematic search in MEDLINE/PubMed, Embase, Web of Science, CINAHL, and LILACS databases, performed from inception to March 11, 2021, and updated on May 15, 2022, using terms related to ‘sarcopenia’ and ‘chronic kidney disease’ without time restriction and language. All detailed search strategy for each database is provided in Supporting Information S1. In this process, one reviewer (JSO) searched and removed duplicates using Covidence (Veritas Health Innovation, Melbourne, Australia).

### Selection criteria

Studies were included based on the CoCoPop mnemonic framework (Condition, Context, and Population).^12^

### Condition

We included cross‐sectional, case–control, or cohort studies that reported the prevalence of sarcopenia and severe sarcopenia according to the well‐established consensus definitions as the European Working Group on Sarcopenia in Older People (EWGSOP; EWGSOP2),^9^, ^13^ the International Working Group on Sarcopenia (IWGS),^14^ the Asian Working Group for Sarcopenia (AWGS; AWGS 2019),^15^, ^16^ and the Foundation for the National Institutes of Health Sarcopenia project (FNIH).^17^ Cutoff values for the diagnoses may be seen in Table S1.

We also included studies that evaluated sarcopenic obesity, according to The European Society for Clinical Nutrition and Metabolism (ESPEN) and the European Association for the Study of Obesity (EASO) as low handgrip strength in addition to obesity.^18^

We also considered studies that reported the prevalence of sarcopenia traits (i.e., low muscle strength, muscle mass, or physical performance) if they used the cutoff values recommended by the consensuses for further subgroup analysis. When multiple studies reported the prevalence of sarcopenia based on the same study, the one with the largest sample size was included.

### Context

No restriction was placed on the setting or context of the included studies. Studies in hospital inpatient services, dialysis centres, outpatient programmes, community‐based facilities, and others were included.

### Population

Adult patients (≥18 years) with CKD in stages 3a−5 (glomerular filtration rate <60 mL/min per 1.73 m^2^) and receiving any kidney replacement therapy (i.e., haemodialysis, peritoneal dialysis, or transplantation) were included.

### Exclusion criteria

Studies that include only individuals in stages 1 and 2, or paediatric patients (i.e., <18 years), cohort studies that did not report prevalence data at baseline moment, used indirect instruments to measure any sarcopenia traits (e.g., SARC‐F, calf circumference, biomarkers, or another self‐reported questionnaire), did not report the diagnosis criteria, animal studies, conference abstracts, theses, and letters to editors were excluded.

### Study selection

Titles, abstracts, and full‐text manuscripts were screened by two independent reviewers (M. P. D. and L. S. A.) according to predetermined eligibility criteria using the COVIDENCE software (Veritas Health Innovation, Melbourne, Australia). Disagreements were resolved by a third reviewer (H. S. R.). Additionally, the reference lists of the included studies were hand‐searched to identify potential additional studies.

### Data extraction

Data extraction of eligible articles was performed independently by the main reviewer (L. S. A.) using a standardized spreadsheet form and a second reviewer (M. P. D.) independently made double‐checks for accuracy in all extractions. The detailed list of information collected is available in details in Table S2. Authors were contacted when additional information was required (reply rate = 15.2%). In cohort studies, baseline data was extracted.

### Methodological quality assessment

The methodological quality of included studies was assessed using the JBI Critical Appraisal Checklist, a tool designed to assess the quality/risk of bias of observational studies that report prevalence data.^12^

### Data analysis

Meta‐analyses of pooled prevalence were calculated using the inverse‐variance obtained from random‐effect models, as the assumption was made that the prevalence of sarcopenia would vary between CKD stages and dialysis modalities, countries, and criteria diagnosis methods. When the study used different consensuses but did not report the overall prevalence, the highest prevalence data was considered for pooled meta‐analyses, despite subgroup analyses being conducted using each diagnostic criterion. Moreover, when a single study reported several prevalence data using different cutoff values and diagnosis methods, we only included in overall meta‐analyses those prevalence data based on a consensus. Studies that reported simultaneously sarcopenia prevalence in different stages of CKD and/or kidney replacement therapies were included in the same meta‐analysis with separate estimates.

We categorized studies, mainly according to the disease stages, in six groups (i) non‐dialysis; (ii) haemodialysis; (iii) peritoneal dialysis; (iv) dialysis (haemodialysis plus peritoneal dialysis); v) kidney transplant; and (vi) CKD‐grouped for those that grouped two or more CKD stages (e.g., stage 3+ dialysis) in the same groups and did not report the prevalence in each stage separately. The heterogeneity between studies was assessed using a forest plot and quantified using the *I* ^2^ statistic.^19^ Publication bias was assessed visually in a funnel plot and by Egger's and Begg's tests.^20^, ^21^ To explore heterogeneity, we performed subgroups meta‐analyses according to CKD stages and dialysis modality, geographical regions, gender, diagnostic criteria, and sarcopenia stages. Moreover, χ^2^ test for subgroup differences was applied. Meta‐regression analyses were conducted to investigate the effects of mean age, sex, and body mass index (BMI) on the overall prevalence. All statistical analyses using the *metaprop* command in STATA 16 (StataCorp LP, College Station, TX, USA).^22^

## Results

### Study selection

**Figure 1:**
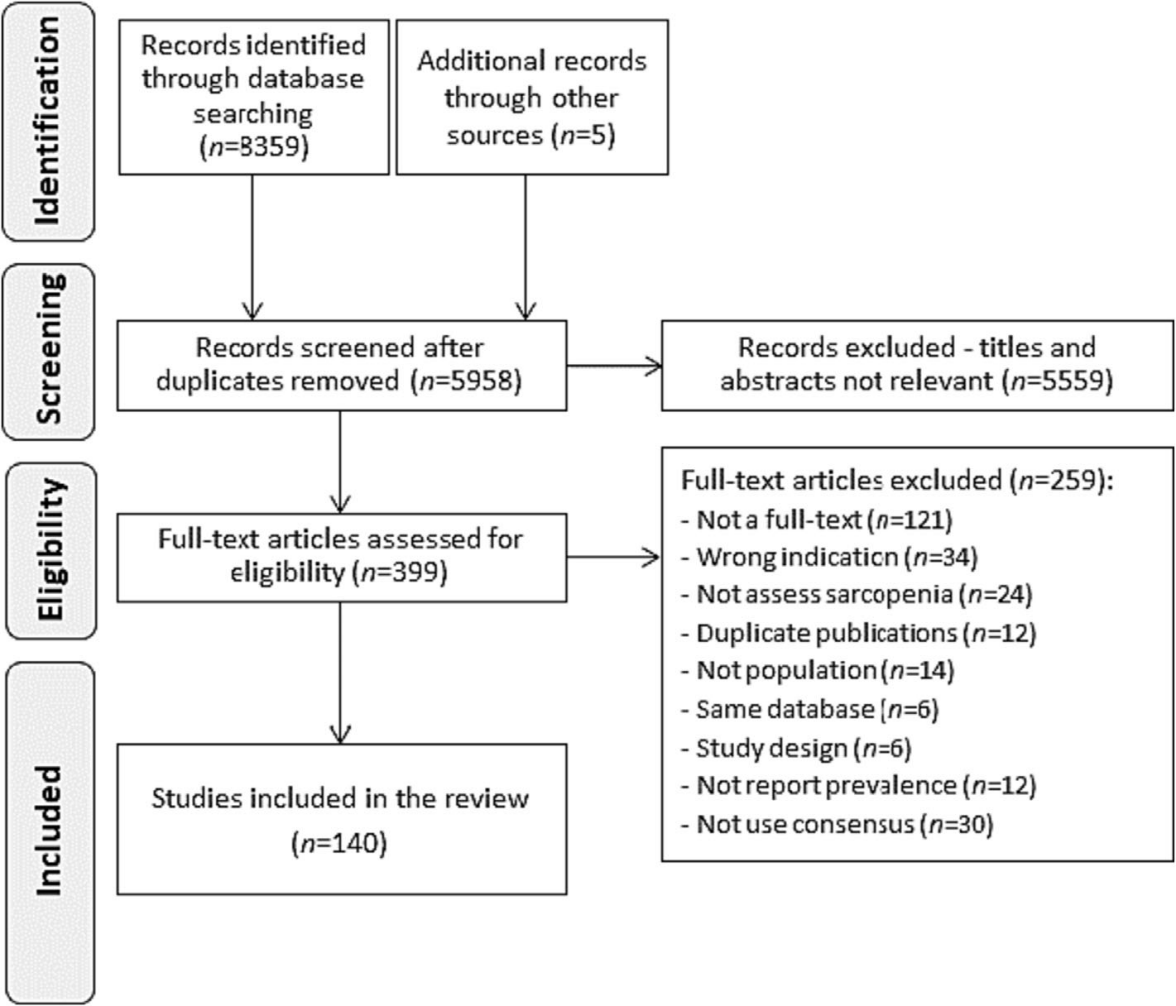
Flow diagram.

The electronic search retrieved 8359 articles, complemented by five records from reference lists of the included studies. A total of 140 studies (42 041 patients) across 25 countries from five continents were included in this review. From these, 114 studies (36 190 patients) were included in the pooled meta‐analysis for sarcopenia and/or severe sarcopenia, while 26 only in the meta‐analysis for sarcopenia traits (Figure 1; and Supporting Information S2 for the full reference list).

### Characteristics of included studies: Narrative synthesis

Among studies in the overall analysis, 90 were cross‐sectional and 24 cohorts. Geographically, most were conducted in Asia (*n* = 70) and Europe (*n* = 16). In general, studies were primarily conducted with the population on haemodialysis (63 studies, 18 190 patients), followed by non‐dialysis (19 studies; 12 908 patients), kidney transplant (13 studies; 1672 patients), and peritoneal dialysis (8 studies; 1283 patients). The study by Chiang et al. was the only one that included men (440 patients).^23^ The median sample size was 130 participants (range 20–8740) and the mean age was 61 years.

Handgrip strength was used in all measures of muscle strength (114 studies; 100%), while bioelectrical impedance (BIA) was the most common method to assess muscle mass (82 studies; 72.6%), followed by dual‐energy x‐ray absorptiometry (31 studies; 27.2%). Gait speed was the most adopted test to assess physical performance (60 studies; 83.3%). Baseline characteristics and diagnosis methods of included studies are available in Table S2.

### Methodological quality

Of the 140 included studies, the median methodological quality score was 6 (IQ 4–7 score). Only eight studies (6%) achieved the maximum score. A non‐probabilistic sample was found in 125 of the studies (94%), and only six were randomized or population‐based (6%). Table S3 summarizes the methodological quality assessment of the included studies.

### Publication bias

Egger's, but not Begg's test, showed evidence for publication bias in the overall meta‐analysis (*P* = 0.006 and 0.174, respectively; Figure S1). When stratifying the prevalence of sarcopenia according to CKD stages, asymmetry was not observed (Figure S2).

### Meta‐analyses

**Figure 2:**
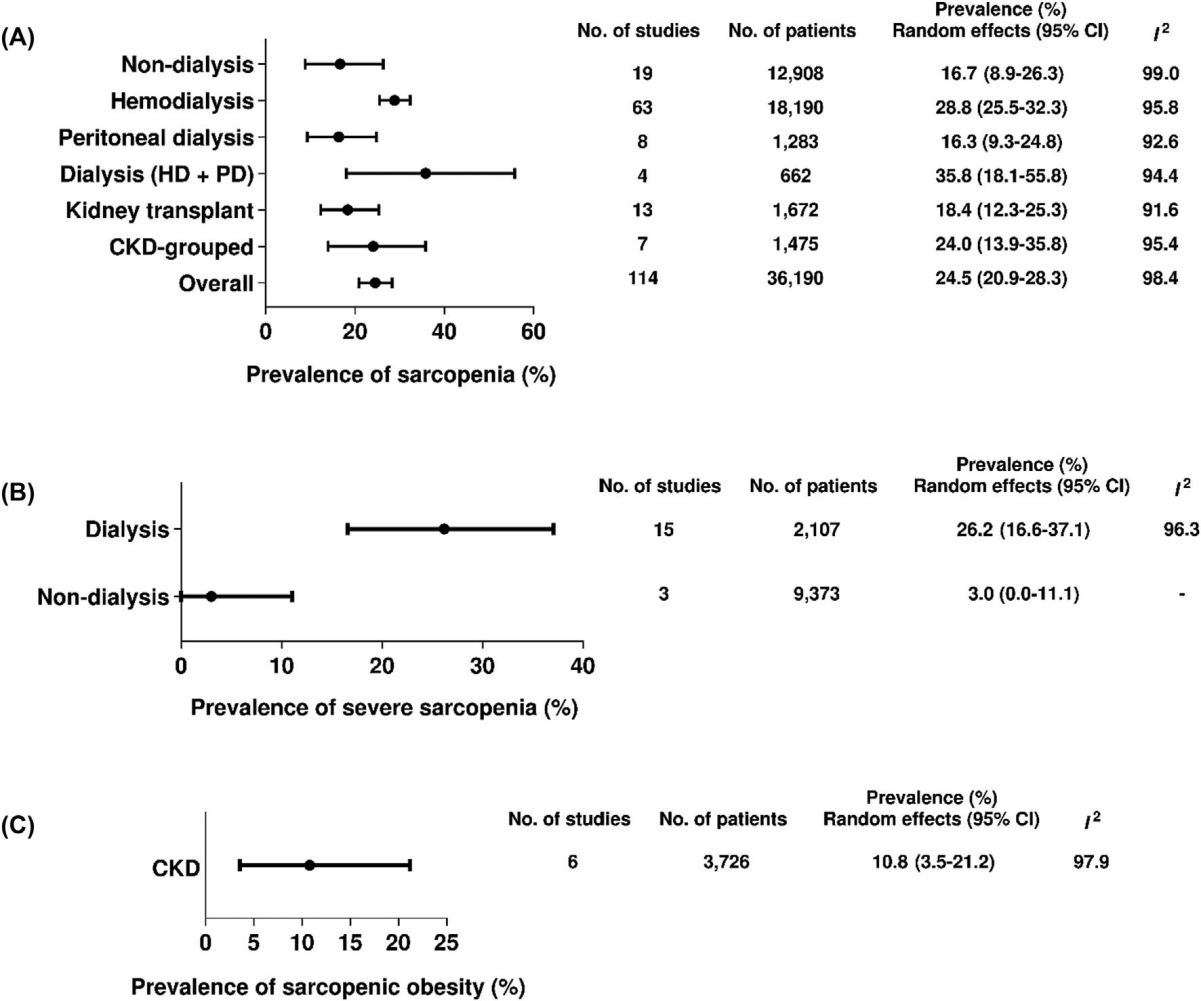
Prevalence of sarcopenia, severe sarcopenia, and sarcopenic obesity in the wide spectrum of CKD.

The overall and subgroup meta‐analyses exploring the prevalence of sarcopenia, severe sarcopenia, and sarcopenic obesity in the wide spectrum of CKD are summarized in Figure 2.

#### Prevalence of sarcopenia

The global prevalence of sarcopenia in CKD patients was 24.5% (95% CI: 20.9–28.3) and did not differ among CKD stages and kidney replacement therapies (subgroup difference: *P* = 0.33). Among individual studies, the prevalence of sarcopenia ranged from 0.8^24^ to 82.5%.^25^ The prevalence did not differ between non‐dialysis and dialysis (16.7, 95% CI: 8.9–26.3 vs. 27.7 95% CI: 24.7–30.9; *P* = 0.08; Table S4).

#### Prevalence of severe sarcopenia

Among the 15 included studies, the prevalence of severe sarcopenia ranged from 0.5^24^ to 75%.^26^ The overall prevalence was 21.0% (95% CI: 11.7–32.0, *I* ^2^: 98.7%), while in dialysis patients, it was 26.2% (95% CI: 16.6–37.1), and in non‐dialysis patients 3.0% (95% CI: 0–11.1; *P* < 0.01; Figure S3).

#### Prevalence of sarcopenic obesity

Five included studies reported the prevalence of sarcopenic obesity (Figure S4). The overall prevalence of sarcopenic obesity in patients with CKD was 10.8% (95% CI: 3.5–21.2) with high heterogeneity (*I* ^2^: 97.6%, *P* < 0.01).

#### Prevalence of sarcopenia traits

**Figure 3:**
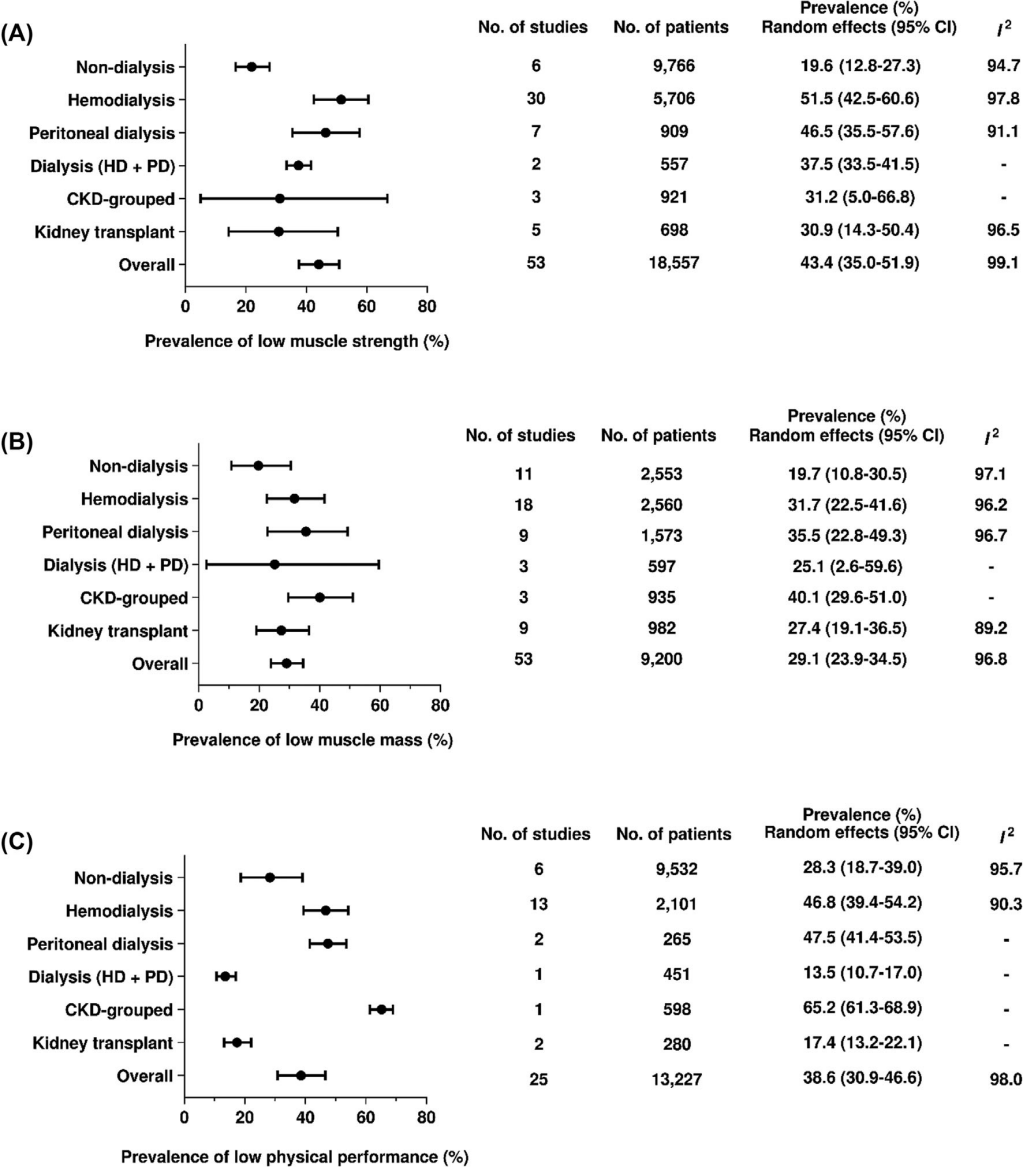
Prevalence of low muscle strength, low muscle mass, and low physical performance in the wide spectrum of CKD.

Figure 3 describes the prevalence of sarcopenia traits (i.e., low muscle strength, low muscle mass, and low physical performance) separately by CKD stages.

##### Low muscle strength

The overall prevalence of low muscle strength was 43.4% (95% CI: 35.0–51.9). A high prevalence was found among patients on kidney replacement therapies, especially haemodialysis (51.5%, 95% CI: 42.5–60.6). A significant difference was observed between dialysis (50.0%, 95% CI: 41.7–57.4, *I* ^2^: 97.4%) and non‐dialysis groups (19.6%, 95% CI: 12.8–27.3, *I* ^2^: 94.7%; subgroup difference: *P* < 0.01).

##### Low muscle mass

The overall prevalence of low muscle mass was 29.1% (95% CI: 23.9–34.5). The prevalence in dialysis was 32.2% (95% CI: 25.2–39.6, *I* ^2^: 96.4%) and the non‐dialysis 19.7% (95% CI: 10.8–30.5, *I* ^2^: 97.1%; subgroup difference: *P* = 0.07), with no significant difference.

##### Low physical performance

The overall prevalence of low physical performance was 38.6 (95% CI: 30.9–46.6). A high prevalence was found among patients on haemodialysis (46.8%, 95% CI: 39.4–54.2) and peritoneal dialysis (47.5%, 95% CI: 41.4–53.5). The prevalence did not differ between non‐dialysis (28.3%, 95% CI: 18.7–39.0, *I* ^2^: 95.7%) and dialysis groups (43.3%, 95% CI: 34.1–52.6, *I* ^2^: 95.7%; subgroup difference: *P* = 0.28).

#### Prevalence of sarcopenia according to the consensus definition

**Figure 4:**
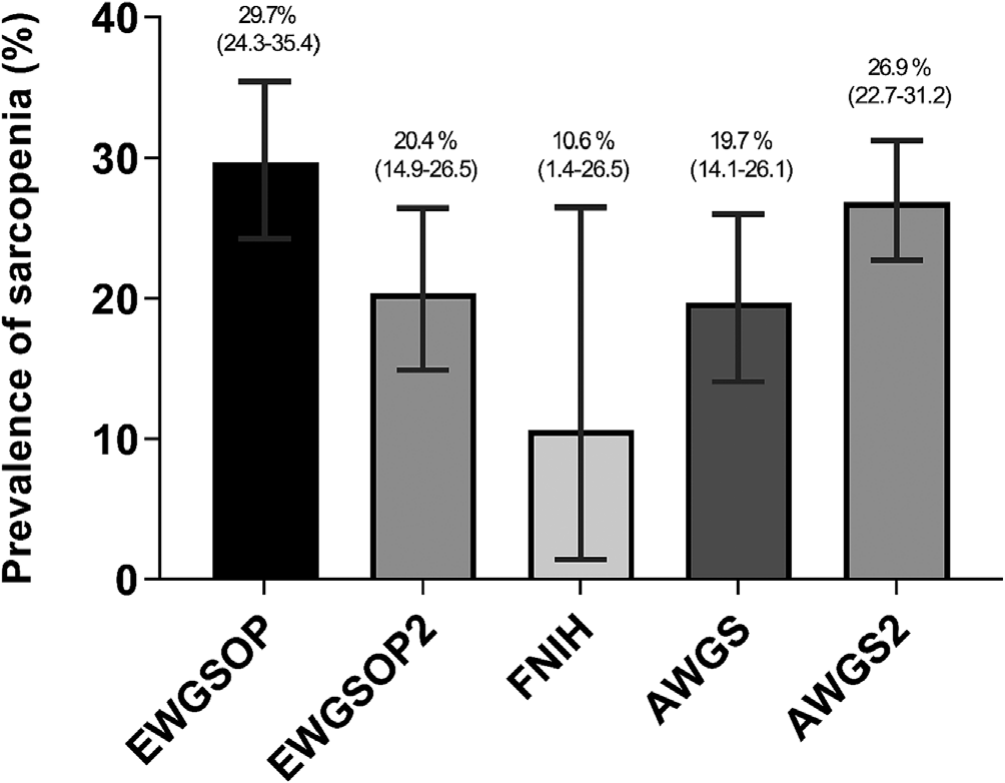
Prevalence of sarcopenia according to the consensus definition.

The prevalence of sarcopenia according to the consensus definition is shown in Figure 4. The estimates indicated no significant difference among the consensus definitions (*P* = 0.423). Virtually, EWGSOP (29.7%, 95% CI: 24.3–35.4) showed the highest prevalence, whereas FNIH was the lowest (10.6%, 95% CI: 1.4–26.5).

The prevalence of sarcopenia stratified by CKD stages and kidney replacement therapy is shown in Table 1 and Figures S5 to S10. There were no significant differences among the CKD stages and kidney replacement therapies within each consensus definitions. However, significant differences were found for the consensus definitions within each CKD stage and kidney replacement therapy, except for the dialysis analysis (haemodialysis + peritoneal dialysis; *P* = 0.061).

**Table 1:** Prevalence of sarcopenia according to the consensus definition stratified by CKD stages and KRT.

	Meta‐analysis		Heterogeneity		Inter‐group comparisons
	No. of studies	No. of patients	Prevalence (95% CI)	*I* ^2^	*P* value
**EWGSOP**					0.607
Non‐dialysis	2	153	39.2 (31.5–47.1)	‐	
Haemodialysis	15	2919	28.8 (21.9–36.1)	94.2%	
Peritoneal dialysis	2	129	10.8 (5.9–16.9)	‐	
Dialysis (HD + PD)	3	211	41.4 (21.4–62.9)	‐	
Kidney transplant	5	689	26.6 (16.1–38.7)	91.3%	
CKD‐grouped	2	285	39.2 (33.6–45.0)	‐	
Overall	29	4386	29.7 (24.3–35.4)	93.7%	
**EWGSOP2**					0.238
Non‐dialysis	4	975	8.4 (1.7–19.1)	95.3%	
Haemodialysis	12	1144	30.6 (22.3–39.5)	89.3%	
Peritoneal dialysis	1	368	11.1 (4.1–27.9)	‐	
Dialysis (HD + PD)	1	451	22.2 (18.6–26.2)	‐	
Kidney transplant	2	295	10.9 (7.5–14.7)	‐	
CKD‐grouped	3	534	13.8 (4.1–27.9)	‐	
Overall	23	3767	20.4 (14.9–26.5)	94.6%	
**FNIH**					0.213
Non‐dialysis	2	8840	0.6 (0.5–0.8)	‐	
Haemodialysis	1	440	17.0 (13.8–20.8)	‐	
Peritoneal dialysis	1	155	15.5 (10.6–22.0)	‐	
Kidney transplant	1	120	3.3 (1.3–8.3)	‐	
Overall	5	9555	10.6 (1.4–26.5)	98.8%	
**AWGS**					0.276
Non‐dialysis	1	260	25.0 (20.1–30.6)	‐	
Haemodialysis	9	7331	21.4 (13.7–30.2)	98.1%	
Peritoneal dialysis	3	445	15.9 (4.0–33.4)	‐	
Kidney transplant	2	268	13.1 (9.2–17.5)	‐	
Overall	15	8304	19.7 (14.1–26.1)	97.2%	
**AWGS2**					0.486
Non‐dialysis	10	2680	17.7 (11.6–24.9)	94.8%	
Haemodialysis	26	6356	31.6 (25.6–38.0)	96.3%	
Peritoneal dialysis	1	186	38.2 (31.5–45.3)	‐	
Kidney transplant	3	300	19.1 (9.0–31.7)	‐	
CKD‐grouped	2	656	28.8 (25.3–32.3)	‐	
Overall	42	10 178	26.9 (22.7–31.2)	95.6%	

CKD, chronic kidney disease; HD, haemodialysis; KRT, kidney replacement therapies; PD, peritoneal dialysis; EWGSOP, European Working Group on Sarcopenia in Older People; AWGS, Asian Working Group for Sarcopenia; FNIH, The Foundation for the National Institutes of Health Biomarkers Consortium Sarcopenia Project.

#### Gender

Prevalence of sarcopenia was 25.8% (95% CI: 22.4–29.3; 7890 participants) in men and 25.7% (95% CI: 21.3–30.4; 5120 participants) in women, with no significant difference (*P* = 0.96). More data according to the CKD stages may be seen in Table S6.

#### Geographical regions

**Figure 5:**
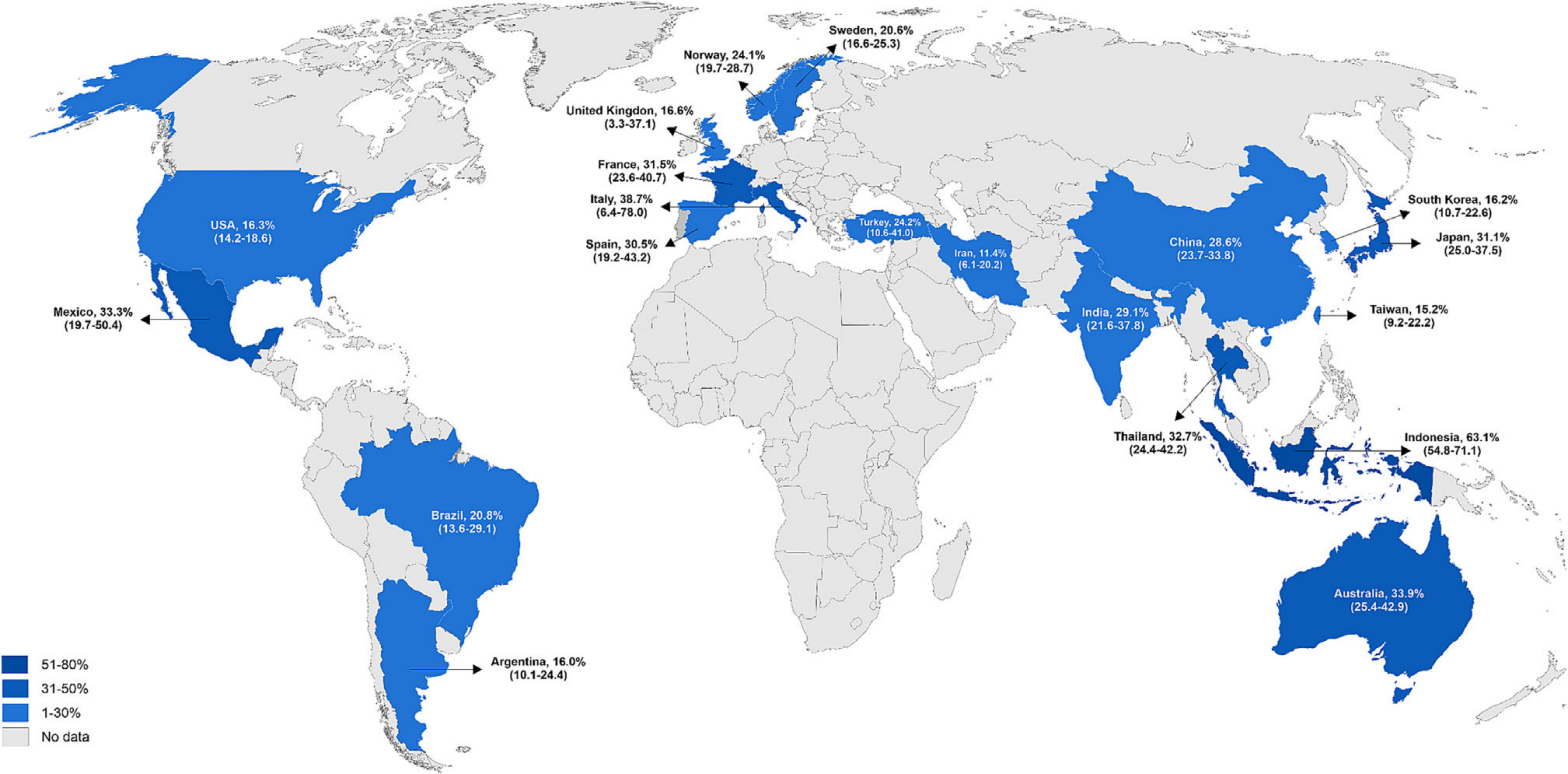
Prevalence of sarcopenia in patients with chronic kidney disease according to the countries.

Figure 5 shows the prevalence of sarcopenia according to the countries. In brief, high rates were found in Indonesia (two studies, 63.1%; 95% CI: 54.8–71.1), Italy (three studies, 38.7%; 95% CI: 6.4–78.0), and Australia (two studies, 33.9%; 95% CI: 25.4–42.9). When the analyses were stratified by Asian (70 studies, 26.0%; 95% CI: 22.9–29.2, *I* ^2^: 95.9%) and non‐Asian countries (43 studies, 22.3%; 95% CI: 15.9–29.4, *I* ^2^: 98.6%; Table S5), no significant difference was observed (*P* = 0.74).

### Meta‐regression

Meta‐regression analyses showed no significant difference for the prevalence of sarcopenia using BIA versus DEXA or comparing low vs. high methodological quality (score >5) (Figures S11 to S12). We found a negative association between the overall prevalence of sarcopenia and the percentage of women in the study sample (*P* = 0.045; residual heterogeneity: *I* ^2^ = 79.9%; *n* = 113 studies); whereas a positive association with the mean age (*P* = 0.006; residual heterogeneity: *I* ^2^ = 80.3%; *n* = 106 studies). On the other hand, percentage of males, BMI, and ethnicity were not statistically significant (Figures S13 to S18).

## Discussion

### Main findings

The main finding from our review is that the global prevalence of sarcopenia among the population with CKD was 25%, with no significant differences among the stages and kidney replacement therapies. Observed prevalence did not significantly vary according to employed operational definitions, but the lowest and highest rates were for the FNIH (11%) and EWGSOP (30%), respectively. Regarding the severity of sarcopenia, we found a higher prevalence of severe sarcopenia in patients on dialysis compared to non‐dialysis (26% vs. 3%). Of relevant note, low muscle strength, the paramount manifestation of sarcopenia, was found in 43% of patients with CKD. Finally, the meta‐regression results show that the higher the mean age of participants in the study, the higher the prevalence of sarcopenia. Interestingly, the percentage of female patients was negatively associated with the prevalence of sarcopenia, suggesting that the anabolic hormonal differences between male and female may not impact the prevalence of sarcopenia in CKD.

Previous systematic reviews have shown that sarcopenia in patients with CKD was associated with several adverse clinical outcomes such as falls, fractures, cardiovascular events, and mortality.^3^, ^27^, ^28^ Since the prevalence of CKD tends to increase globally due to population aging, action plans are needed for early sarcopenia screening and management in the CKD population.

### Prevalence of sarcopenia

As sarcopenia is currently considered a disease,^29^ determining severity stages is crucial to understand how this may affect clinical outcomes. In adults without CKD, a systematic review showed a global prevalence of sarcopenia varying from 10% to 27%, while severe sarcopenia ranged from 2% to 9%.^30^ As expected, our study showed an overall prevalence slightly higher of sarcopenia (1 to 83%) and severe sarcopenia (1% to 75%) than in the general population. Systematic reviews and meta‐analysis summarizing the prevalence of sarcopenia in CKD are scarce and mainly limited to dialysis patients and kidney transplantation recipients. Wathanavasin et al.^27^ reported a pooled sarcopenia prevalence of 25.6% (95% CI: 22.1 to 29.4) in dialysis patients, similar to our findings on dialysis in the present study (27%, 95% CI: 24 to 31) and in the study by Shu et al.^4^ (29%, 95% CI: 23 to 34). Zhang et al.^8^ observed a pooled prevalence of 26% (95% CI: 20 to 34) in kidney transplantation recipients; however, most studies diagnosed sarcopenia only in terms of low muscle mass, a definition that is not currently endorsed by expert committees (SDOC, FNIH, and EWGSOP). Interestingly, Zhang et al. reported a sub analysis with the studies defining sarcopenia using low muscle mass in combination with low muscle strength or low physical performance, and the observed prevalence was 21% (95% CI: 15 to 28), a rate that was like the subgroup of kidney recipients in the present study (21%, 95% CI: 14 to 28).

In patients with CKD, sarcopenia has been associated with and mainly explained by a high protein degradation due to chronic low‐grade inflammation, insulin resistance, metabolic acidosis, and increased oxidative stress.^6^ This negative protein imbalance is known to be worse in more advanced stages of the disease. Despite this, our findings showed that the prevalence of sarcopenia did not significantly differ between non‐dialysis and dialysis patients. Nevertheless, we identified a significantly higher prevalence of severe sarcopenia in patients on dialysis. These observations indicate that although the prevalence of sarcopenia might not differ according to dialysis status, the frequency of severe sarcopenia and related consequences is higher among patients requiring dialysis. Severe sarcopenia (i.e., low levels of muscle strength, muscle quantity, and physical performance) is associated with significantly worse outcomes than non‐severe sarcopenia.^24^ It is documented that the dialysis procedure itself commonly stimulates protein degradation and negatively impacts protein synthesis, which may lead to faster muscle wasting.^5^ Thus, declines in physical performance may occur earlier than in non‐dialysis patients, indicating that patients on dialysis should be assessed for sarcopenia severity more often, allowing early implementation of therapeutic strategies.

### Prevalence of sarcopenic obesity

Sarcopenic obesity is a clinical condition defined by the coexistence of sarcopenia and body fat excess and has been examined as an emerging public health problem worldwide.^18^ The adverse clinical consequences of sarcopenic obesity have been considered of paramount importance, and worse than sarcopenia or obesity alone.^18^, ^31^ As a secondary aim, we sought to identify the global prevalence of sarcopenic obesity among CKD individuals, which was found to be slightly higher than 10%. It should be noted, however, that only five studies have presented sarcopenic obesity prevalence in this population and a high heterogeneity in reported rates was noted.

In contrast to sarcopenia, there is a lack of consensual diagnostic criteria for sarcopenic obesity, which clearly affects its reliable evaluation of prevalence and may explain heterogeneous results. Despite this, the observed prevalence was virtually the same as that reported for older individuals in a recent meta‐analysis (11%)^32^ and supports the concept that CKD is associated with signs of premature aging. Our findings are in line with a previous report that described a high prevalence of central obesity and sarcopenia in CKD, irrespective of disease stage.^33^ Attention to recognizing the signs of sarcopenic obesity in nephrology patients is warranted, as well as early implementation of therapeutic strategies to improve prognosis.

### Prevalence of sarcopenia traits

We recognized low muscle strength, low muscle mass, and low physical performance as sarcopenia traits. Almost half of the patients with CKD had low muscle strength, while only one in four had low muscle mass. Given that low muscle strength has been established to be more strongly associated with adverse outcomes than low muscle mass,^3^, ^34^ this high prevalence of low muscle strength holds significant clinical implications for patients with CKD. In fact, the EWGSOP2 now defines low muscle strength as the key characteristic of sarcopenia, overtaking the role of low muscle mass as a principal determinant.^9^

The results comparing dialysis and non‐dialysis groups showed a higher prevalence of low muscle strength in those on dialysis, while muscle mass and physical performance did not. These findings suggest that the chronic catabolic state experienced by patients on dialysis has a detrimental impact on muscle strength, the primary landmark of sarcopenia.

### Prevalence of sarcopenia by consensus definitions

Our results identified that prevalence rates of sarcopenia in CKD were virtually different among the applied consensus definition, however, statistical analysis showed no significant differences. Virtually, the highest rate was for the EWGSOP, whereas the lowest for the FNIH. It should be noted that the IWGS consensus was employed in only two of the included studies, which may explain the highest prevalence. FNIH consensus, for instance, adjusts appendicular skeletal muscle mass based on BMI, which can potentially lead to an underestimation of the sarcopenia diagnosis in patients with suboptimal BMI.^35^ On the other hand, the most used consensus (EWGSOP, EWGSOP2, AWGS, and AWGS2) showed very similar rates.

These findings highlight that discussions on what consensus to adopt are relevant. Nevertheless, the perfect may be the enemy of the good; thereby, the existence of multiple tools and consensuses for assessing sarcopenia can facilitate easier screening and diagnosis in clinical practice. Clinicians and health professionals should have in mind that consistency and homogeneity are important for inter‐group comparisons over time.

### Prevalence of sarcopenia by gender and geographic region

In contrast with our results, the literature suggests that gender may potentially impact the severity of sarcopenia‐related traits such as low handgrip strength, and raises the hypothesis that men in a uremic state may exhibit increased susceptibility to appetite loss, inflammation, and subsequent musculoskeletal imbalances.^36^, ^37^ Regarding geographic regions, we found no significant differences between Asian and non‐Asian countries. Although lifestyle factors, such as food habits and physical activity behaviour, are significant predictors of sarcopenia and vary between countries, our results are in line with previous studies investigating the prevalence of sarcopenia in people without CKD.^30^

### Clinical applicability

The results from this review have clinical applicability to health professionals involved in the care of patients with CKD. As well‐reported in the literature, patients with CKD diagnosed with sarcopenia and/or its traits have an increased mortality risk.^3^, ^4^ Our findings showed that one in four patients with CKD have sarcopenia and that almost half of them present low muscle strength, the key trait of sarcopenia and of strong prognostic value. These compelling results strongly suggest that sarcopenia screening should be included in the clinical management of CKD. In this regard, while body composition assessment is often unavailable due to high equipment costs, physical function measures are cost‐effective and easy to incorporate into everyday clinical practice. Also, the SARC‐F questionnaire was developed as a rapid screening tool for sarcopenia, and updated versions have been proposed since then, such as the SARC‐CalF which by adding a calf circumference measurement has been shown to increase the sensibility to detect sarcopenia in patients on haemodialysis.^38^ Moreover, these findings highlight the critical need for clinicians to prescribe effective targeted interventions that may counterattack this phenomenon, such as resistance exercise training and adequate caloric and protein intake.^39^ Moreover, we encourage the use of a consensus definition based on the geographic location where it is available, for example, the AWGS for Asians, the EWGSOP2 for Europeans, and the FNIH for North Americans.

### Strengths and limitations

To the best of our knowledge, this was the first systematic review with meta‐analysis to identify the prevalence of sarcopenia across the entire spectrum of CKD. Only a handful of previous studies have reported the prevalence of sarcopenia in CKD, but data were limited to specific stages of the disease (i.e., dialysis patients or kidney transplantation recipients) and had the main purpose of examining the association between sarcopenia and clinical outcomes. The present review included 140 studies from 25 different countries across five continents, representing a global perspective. We applied multiple sub‐group analyses and meta‐regressions to identify potential sources of heterogeneity. Given the diverse consensuses of sarcopenia diagnosis available, we provided a comprehensive overview of the current scenario by analysing the most relevant consensuses and operational definitions.

Although this review has strengths, some limitations deserve to be mentioned. First, our results were based on observational findings, so cause‐and‐effect relationships cannot be established. Second, we identified high heterogeneity in sarcopenia prevalence estimates. Third, although the entire review process was performed by two reviewers, the different operational definitions of sarcopenia may lead to selection bias at this stage. Finally, although this review included studies from most continents, data from Africa was not found.

## Conclusions

Our review identified a high global prevalence of sarcopenia across the entire CKD spectrum, with no significant difference among stages and kidney replacement therapies. Also, sarcopenia prevalence rates did not significantly vary according to the adopted consensus definition. Of relevant note, low muscle strength, the primary manifestation of sarcopenia with important prognostic value, was present in almost half of the CKD population, whereas patients on dialysis were more prevalent to low muscle strength and severe sarcopenia.

The results presented in this systematic review provide support for the inclusion of sarcopenia screening in clinical settings and early implementation of targeted interventions (e.g., nutrition and exercise) to counteract the decline in muscle mass, strength, and performance in people with CKD, especially those dialysis‐dependent.

## Conflicts of interest

The authors declare they have no conflict of interest.

## Supporting information


**Supporting Information S1.** Search strategies.
**Supporting Information S2.** Reference list of the included studies.
**Table S1.** Cutoff values for the consensus definition of sarcopenia.
**Table S2.** Characteristics of the studies included in the systematic review.
**Table S3:** JBI tool for methodological quality assessment of the included studies.
**Table S4.** Pooled prevalence of sarcopenia in patients with chronic kidney disease.
**Table S5.** Prevalence of sarcopenia by Asia vs non‐Asian countries.
**Table S6.** Prevalence of sarcopenia stratified by gender according to the stages of CKD and KRT.
**Figure S1.** Funnel plot for sarcopenia prevalence in patients with chronic kidney disease.
**Figure S2.** Funnel plots of studies reporting sarcopenia prevalence stratified by CKD subgroups.
**Figure S3.** Pooled prevalence of severe sarcopenia in patients with chronic kidney disease.
**Figure S4.** Pooled prevalence of sarcopenic obesity in patients with chronic kidney disease. CI, confidence interval; CKD; chronic kidney disease.
**Figure S5.** Pooled prevalence of sarcopenia according to diagnosis consensus in non‐dialysis patients. AWGS, Asian Working Group for Sarcopenia; CI, confidence interval; EWGSOP, European Working Group on Sarcopenia in Older People; FNIH; Foundation for the National Institutes of Health Sarcopenia Project.
**Figure S6.** Pooled prevalence of sarcopenia according to diagnosis consensus in patients on haemodialysis.
**Figure S7.** Pooled prevalence of sarcopenia according to diagnosis consensus in patients on peritoneal dialysis. AWGS, Asian Working Group for Sarcopenia; CI, confidence interval; EWGSOP, European Working Group on Sarcopenia in Older People; FNIH; Foundation for the National Institutes of Health Sarcopenia Project.
**Figure S8.** Pooled prevalence of sarcopenia according to diagnosis consensus in patients on dialysis (haemodialysis + peritoneal dialysis).
**Figure S9.** Pooled prevalence of sarcopenia according to diagnosis consensus in kidney transplant patients.
**Figure S10.** Pooled prevalence of sarcopenia according to diagnosis consensus in CKD‐grouped patients.
**Figure S11.** Pooled prevalence of sarcopenia according to device mass tool.
**Figure S12.** Pooled prevalence of sarcopenia according to methodological quality.
**Figure S13.** Bubble plot of sarcopenia prevalence by the percentage of women in the study sample.
**Figure S14.** Bubble plot of sarcopenia prevalence by the percentage of men in the study sample.
**Figure S15.** Bubble plot of sarcopenia prevalence by the mean age of patients.
**Figure S16.** Bubble plot of sarcopenia prevalence by the percentage of body mass index of patients.
**Figure S17.** Bubble plot of sarcopenia prevalence by the percentage of white patients.
**Figure S18.** Bubble plot of sarcopenia prevalence by the percentage of black patients.
