# Supplementary material for: Prevalence of sarcopenia in patients with chronic kidney disease: a global systematic review and meta‐analysis
Source: J Cachexia Sarcopenia Muscle. 2024 Jan 24;15(2):501–12. doi: 10.1002/jcsm.13425 (PMC10995263; doi:10.1002/jcsm.13425)
Supplement: Supplementary file 1 — Supporting Information S1. Search strategies. Supporting Information S2. Reference list of the included studies. Table S1. Cutoff values for the consensus definition of sarcopenia. Table S2. Characteristics of the studies included in the systematic review. Table S3: JBI tool for methodological quality assessment of the included studies. Table S4. Pooled prevalence of sarcopenia in patients with chronic kidney disease. Table S5. Prevalence of sarcopenia by Asia vs non‐Asian countries. Table S6. Prevalence of sarcopenia stratified by gender according to the stages of CKD and KRT. Figure S1. Funnel plot for sarcopenia prevalence in patients with chronic kidney disease. Figure S2. Funnel plots of studies reporting sarcopenia prevalence stratified by CKD subgroups. Figure S3. Pooled prevalence of severe sarcopenia in patients with chronic kidney disease. Figure S4. Pooled prevalence of sarcopenic obesity in patients with chronic kidney disease. CI, confidence interval; CKD; chronic kidney disease. Figure S5. Pooled prevalence of sarcopenia according to diagnosis consensus in non‐dialysis patients. AWGS, Asian Working Group for Sarcopenia; CI, confidence interval; EWGSOP, European Working Group on Sarcopenia in Older People; FNIH; Foundation for the National Institutes of Health Sarcopenia Project. Figure S6. Pooled prevalence of sarcopenia according to diagnosis consensus in patients on haemodialysis. Figure S7. Pooled prevalence of sarcopenia according to diagnosis consensus in patients on peritoneal dialysis. AWGS, Asian Working Group for Sarcopenia; CI, confidence interval; EWGSOP, European Working Group on Sarcopenia in Older People; FNIH; Foundation for the National Institutes of Health Sarcopenia Project. Figure S8. Pooled prevalence of sarcopenia according to diagnosis consensus in patients on dialysis (haemodialysis + peritoneal dialysis). Figure S9. Pooled prevalence of sarcopenia according to diagnosis consensus in kidney transplant patients. Figure S10. Pooled [file JCSM-15-501-s001.docx]

**Supporting Information 1.** Search strategies.

**MEDLINE (OvidSP) – 903 references (Dated 11 March 2021), additional 203 references (Dated 15 May 2022), additional 400 (25 September 2023)**

1. exp Renal Insufficiency, Chronic/
2. (chronic kidney disease or chronic renal disease).tw,kf.
3. ((kidney or renal) adj5 (insufficienc* or failure*)).tw,kf.
4. exp Renal Dialysis/ or Kidney Transplantation/
5. ((renal or kidney) adj3 (transplant* or replacement*)).tw,kf.
6. (predialysis or pre-dialysis or dialysis or hemodialys* or haemodialysis or hemofiltration or haemofiltration or hemodiafiltration or haemodiafiltration or peritoneal dialys*).tw,kf.
7. or/1-6
8. muscular atrophy/ or sarcopenia/ or muscle weakness/ or muscle strength/
9. (sarcopeni* or dynapeni* or muscle weakness).tw,kf.
10. (EWGSOP or EWGSOP2 or AWGS or FNIH or IWGS).tw,kf.
11. or/8-10
12. 7 and 11
13. exp animals/ not humans/
14. 12 not 13
15. exp clinical trial/
16. 14 not 15
17. review.pt.
18. 16 not 17

**Embase (OvidSP) – 2513 references (Dated 11 March 2021), additional 432 references (Dated 15 May 2022) , additional 710 (25 September 2023)**

1. exp renal insufficiency, chronic/
2. (chronic kidney disease or chronic renal disease).tw.
3. ((kidney or renal) adj5 (insufficienc* or failure*)).tw.
4. exp renal dialysis/ or kidney transplantation/
5. ((renal or kidney) adj3 (transplant* or replacement*)).tw.
6. (predialysis or pre-dialysis or dialysis or hemodialys* or haemodialysis or hemofiltration or haemofiltration or hemodiafiltration or haemodiafiltration or peritoneal dialys*).tw.
7. or/1-6
8. muscular atrophy/ or sarcopenia/ or muscle weakness/ or muscle strength/
9. (sarcopeni* or dynapeni* or muscle weakness).tw
10. (EWGSOP or EWGSOP2 or AWGS or FNIH or IWGS).tw.
11. or/8-10
12. 7 and 11
13. (exp animals/ or nonhuman/) not exp human/
14. 12 not 13
15. exp clinical trial/
16. 14 not 15
17. (review or editorial or letter).pt.
18. 16 not 17

**CINAHL (Ebsco) – 377 references (Dated 11 March 2021), additional 72 references (Dated 15 May 2022) , additional 10 (2 October 2023)**

1. MH Renal Insufficiency, Chronic+
2. TI (renal insufficienc* OR kidney insufficienc* OR renal diseas* OR kidney diseas* OR kidney failure* OR renal failure*) OR AB (renal insufficienc* OR kidney insufficienc* OR renal diseas* OR kidney diseas* OR kidney failure* OR renal failure*)
3. TI (chronic kidney disease OR chronic renal disease) OR AB (chronic kidney disease OR chronic renal disease)
4. MH renal dialysis+ or MH kidney transplantation
5. TI (non-dialysis OR predialysis OR pre-dialysis OR dialysis OR hemodialys* OR haemodialysis OR hemofiltration OR haemofiltration OR hemodiafiltration OR haemodiafiltration OR peritoneal dialys* OR renal transplant* OR renal replacement* OR kidney transplant* OR kidney replacement*) OR AB (non-dialysis OR predialysis OR pre-dialysis OR dialysis OR hemodialys* OR haemodialysis OR hemofiltration OR haemofiltration OR hemodiafiltration OR haemodiafiltration OR peritoneal dialys* OR renal transplant* OR renal replacement* OR kidney transplant* OR kidney replacement*)
6. S1 OR S2 OR S3 OR S4 OR S5
7. MH muscular atrophy OR MH sarcopenia OR MH muscle weakness OR MH muscle strength
8. TI (sarcopeni* OR dynapeni* OR muscle weakness) OR AB (sarcopeni* OR dynapeni* OR muscle weakness)
9. TI (EWGSOP OR EWGSOP2 OR AWGS OR FNIH OR IWGS) OR AB (EWGSOP OR EWGSOP2 OR AWGS OR FNIH OR IWGS)
10. S7 OR S8 OR S9
11. S6 AND S10
12. MH animals+ not MH humans
13. S11 NOT S12
14. MH Clinical Trials+
15. S13 NOT S14
16. PT review OR PT systematic review
17. S15 NOT S16

**LILACS (BVS) – 27 references (Dated 11 March 2021), additional 01 references (Dated 15 May 2022) , additional 2 (25 September 2023)**

1. (Renal Insufficiency OR Renal Insufficiency, Chronic OR Renal failure OR Kidney Failure, Chronic OR Chronic kidney disease OR Kidney Insufficienc$ OR Renal disease OR Kidney diseas$ OR Chronic renal disease OR Renal replacement therapy OR Hemofiltration OR Haemofiltration OR Hemodiafiltration OR Haemodiafiltration OR Renal Dialysis OR Haemodialysis OR Hemodialys$ OR Dialysis OR Non-dialysis OR Predialysis OR Pre-dialysis OR Kidney transplant$ OR Kidney replacement$ OR Renal transplant$ OR Renal replacement$ OR Peritoneal dialys$) AND (Muscular atrophy OR Sarcopenia OR Muscle Weakness OR Muscle strength OR Sarcopeni$ OR Dynapeni$ OR EWGSOP OR EWGSOP2 OR AWGS OR FNIH OR IWGS)

**Web of Science –1807 references (Dated 11 March 2021), additional 327 references (Dated 15 May 2022) additional 643 (2 October 2023)**

1. TS=("chronic kidney disease" OR "chronic kidney insufficienc$" OR "chronic renal disease" OR "chronic renal insufficienc$" OR "kidney diseas$" OR "kidney failure" OR "kidney insufficienc$" OR "renal insufficienc$" OR "renal failure$" OR "renal diseas$")
2. TS=(renal dialysis OR renal replacement therapy OR renal transplant$ OR renal replacement$ OR kidney transplant$ OR kidney replacement$)
3. TS=(non-dialysis OR predialysis OR pre-dialysis OR hemodialys$ OR haemodialysis OR hemofiltration OR haemofiltration OR hemodiafiltration OR haemodiafiltration OR peritoneal dialys$)
4. OR/1-3
5. TS=(muscle atrophy OR sarcopenia OR muscle weakness OR muscle strength)
6. TS=(sarcopeni$ or dynapeni$ or muscle weakness)
7. TS=(EWGSOP OR EWGSOP2 OR AWGS OR FNIH OR IWGS)
8. OR/5-7
9. #4 and #8
10. TI=("controlled trial" OR "clinical trial")
11. #9 NOT #10
12. TI=review
13. #11 NOT #12

**Supporting Information 2.** Reference list of the included studies.

1. Fu C, Yan D, Wang L, Duan F, Gu D, Yao N, et al. High prevalence of sarcopenia and myosteatosis in patients undergoing hemodialysis. Front Endocrinol (Lausanne) [Internet]. 2023 Mar 23;14. Available from: https://www.frontiersin.org/articles/10.3389/fendo.2023.1117438/full

2. Hou YC, Liu YM, Liao MT, Zheng CM, Lu CL, Liu WC, et al. Indoxyl sulfate mediates low handgrip strength and is predictive of high hospitalization rates in patients with end-stage renal disease. Front Med (Lausanne) [Internet]. 2023 Feb 2;10. Available from: https://www.frontiersin.org/articles/10.3389/fmed.2023.1023383/full

3. Elder M, Moonen A, Crowther S, Aleksova J, Center J, Elder GJ. Chronic kidney disease-related sarcopenia as a prognostic indicator in elderly haemodialysis patients. BMC Nephrol [Internet]. 2023 May 19;24(1):138. Available from: https://bmcnephrol.biomedcentral.com/articles/10.1186/s12882-023-03175-5

4. Du W, Gao C, Wang X, Ma X, Xie J, Yu H, et al. Validity of the SARC-F questionnaire in assessing sarcopenia in patients with chronic kidney disease: a cross-sectional study. Front Med (Lausanne) [Internet]. 2023 Jul 18;10. Available from: https://www.frontiersin.org/articles/10.3389/fmed.2023.1188971/full

5. Sánchez-Tocino M, Miranda-Serrano B, López-González A, Villoria-González S, Pereira-García M, Gracia-Iguacel C, et al. Sarcopenia and Mortality in Older Hemodialysis Patients. Nutrients [Internet]. 2022 Jun 5;14(11):2354. Available from: https://www.mdpi.com/2072-6643/14/11/2354

6. Kang SH, Kim AY, Do JY. Association of sarcopenia and its components with clinical outcomes in patients undergoing peritoneal dialysis. Kidney Res Clin Pract [Internet]. 2022 Nov 30;41(6):741–52. Available from: http://krcp-ksn.org/journal/view.php?doi=10.23876/j.krcp.21.278

7. Cai G, Ying J, Pan M, lang X, Yu W, Zhang Q. Development of a risk prediction nomogram for sarcopenia in hemodialysis patients. BMC Nephrol [Internet]. 2022 Sep 23;23(1):319. Available from: https://bmcnephrol.biomedcentral.com/articles/10.1186/s12882-022-02942-0

8. Xiang T, Fu P, Zhou L. Sarcopenia and osteosarcopenia among patients undergoing hemodialysis. Front Endocrinol (Lausanne). 2023;14.

9. Lee SM, Han MY, Kim SH, Cha RH, Kang SH, Kim JC, et al. Indoxyl Sulfate Might Play a Role in Sarcopenia, While Myostatin Is an Indicator of Muscle Mass in Patients with Chronic Kidney Disease: Analysis from the RECOVERY Study. Toxins (Basel). 2022 Oct 1;14(10).

10. Wang Y, Ma W, Pu J, Chen F. Interrelationships between sarcopenia, bone turnover markers and low bone mineral density in patients on hemodialysis. Ren Fail. 2023;45(1).

11. Song P, Xu X, Zhao Y, Gu M, Chen X, Zhang H, et al. Different stages of chronic kidney disease are associated with physical performance in adults over 60 years. Front Public Health [Internet]. 2022 Sep 9;10. Available from: https://www.frontiersin.org/articles/10.3389/fpubh.2022.963913/full

12. Chen R, Zhang L, Zhang M, Wang Y, Liu D, Li Z, et al. The triglyceride-glucose index as a novel marker associated with sarcopenia in non-diabetic patients on maintenance hemodialysis. Ren Fail. 2022;44(1):1615–21.

13. Wu J, Lin S, Guan J, Wu X, Ding M, Shen S. Prediction of the sarcopenia in peritoneal dialysis using simple clinical information: A machine learning-based model. Semin Dial. 2023 Sep 1;36(5):390–8.

14. Yoshikoshi S, Yamamoto S, Suzuki Y, Imamura K, Harada M, Osada S, et al. Associations between dynapenia, cardiovascular hospitalizations, and all-cause mortality among patients on haemodialysis. J Cachexia Sarcopenia Muscle. 2022 Oct 1;13(5):2417–25.

15. Jauwerissa R, Marbun MBH, Nugroho P, Rinaldi I, Suhardjono S, Shatri H, et al. Factors Associated with Sarcopenia in Maintenance Hemodialysis Patients: A Cross-Sectional Study. Acta Med Indones [Internet]. 2023 Jan;55(1):26–32. Available from: http://www.ncbi.nlm.nih.gov/pubmed/36999268

16. Kosoku A, Iwai T, Kabei K, Nishide S, Machida Y, Uchida J. Frailty and sarcopenia in older kidney transplant recipients: a cross-sectional study. Eur Geriatr Med. 2023 Aug 1;14(4):861–8.

17. Chen X, Zhu X, Han P, Zhang Y, He M, Zhang Y, et al. Sarcopenia is associated with mild-to-moderate chronic kidney disease in Chinese community-dwelling older men but not in women. Journal of International Medical Research. 2022 Nov 1;50(11).

18. Xie D, Zhu Q, Lu J, Hu C, Niu J, Yu C, et al. Development and validation of a diagnostic nomogram for sarcopenia in Chinese hemodialysis patients. Nephrology Dialysis Transplantation. 2023 Apr 1;38(4):1017–26.

19. Zhou C, Zhan L, He PH, Yuan J, Zha Y. Associations of sarcopenic obesity vs either sarcopenia or obesity alone with cognitive impairment risk in patients requiring maintenance hemodialysis. Nutrition in Clinical Practice. 2023 Oct 1;38(5):1115–23.

20. Dahl H, Rosendahl-Riise H, Marti HP, Dierkes J. The Association of Sarcopenia and Central Obesity with Mortality Risk in Patients with Chronic Kidney Disease – a 2-Year Observational Study. Curr Dev Nutr. 2023 Jan 1;7(1).

21. Rao NS, Chandra A, Saran S, Lohiya A. Ultrasound for thigh muscle thickness is a valuable tool in the diagnosis of sarcopenia in Indian patients with predialysis chronic kidney disease. Osteoporos Sarcopenia. 2022 Jun;8(2):80–5.

22. Plytzanopoulou P, Papasotiriou M, Politis P, Papachrysanthou T, Andriopoulos C, Drakou A, et al. Cardiac valve calcification in patients on maintenance dialysis. The role of malnutrition-inflammation syndrome, adiposity andcomponents of sarcopenia. A cross-sectional study. Clin Nutr ESPEN. 2022 Dec 1;52:421–30.

23. Kakita D, Matsuzawa R, Yamamoto S, Suzuki Y, Harada M, Imamura K, et al. Simplified discriminant parameters for sarcopenia among patients undergoing haemodialysis. J Cachexia Sarcopenia Muscle. 2022 Dec 1;13(6):2898–907.

24. Shin J, Hwang JH, Han M, Cha RH, Kang SH, An WS, et al. Phase angle as a marker for muscle health and quality of life in patients with chronic kidney disease. Clinical Nutrition. 2022 Aug 1;41(8):1651–9.

25. de Luca Corrêa H, Gadelha AB, Vainshelboim B, Dutra MT, Ferreira-Júnior JB, Deus LA, et al. Could sarcopenia-related mortality in end-stage renal disease be underpinned by the number of hospitalizations and cardiovascular diseases? Int Urol Nephrol. 2023 Jan 1;55(1):157–63.

26. Costa MS da, Pontes KS da S, Guedes MR, Barreto Silva MI, Klein MRST. Association of habitual coffee consumption with obesity, sarcopenia, bone mineral density and cardiovascular risk factors: A two-year follow-up study in kidney transplant recipients. Clinical Nutrition. 2023 Oct 1;42(10):1889–900.

27. Yajima T, Yajima K. Serum creatinine-to-cystatin C ratio as an indicator of sarcopenia in hemodialysis patients. Clin Nutr ESPEN. 2023 Aug 1;56:200–6.

28. Baltacı MA, Atmis V, Metin Y, Aktar M, Eren SA, Sengul S, et al. Sarcopenia and cardiovascular risk indices: Its impact on cardiovascular events and mortality in dialysis patients. Semin Dial. 2023 May 1;36(3):221–30.

29. Zhang M, Zhang L, Hu Y, Wang Y, Xu S, Xie X, et al. Sarcopenia and echocardiographic parameters for prediction of cardiovascular events and mortality in patients undergoing maintenance hemodialysis. PeerJ. 2022 Nov 23;10.

30. Du X, Chen G, Zhang H, Liu Y, Gu F, Wang Y, et al. Development of a Practical Screening Tool to Predict Sarcopenia in Patients on Maintenance Hemodialysis. Medical Science Monitor. 2022;28.

31. Fan Z, Guo Y, Zhong XY. Circulating Cell-Free Mitochondrial DNA: A Potential Blood-Based Biomarker for Sarcopenia in Patients Undergoing Maintenance Hemodialysis. Medical Science Monitor. 2021;28.

32. Yang Y, Da J, Yuan J, Zha Y. One-year change in sarcopenia was associated with cognitive impairment among haemodialysis patients. J Cachexia Sarcopenia Muscle. 2023 Oct 1;

33. Zhang XT, Xiao FL, Xuan F, Gao DM, Zhao J, Li MX. Relationship between skeletal muscle reduction and quality of life and mental status in maintenance dialysis patients. Academic Journal of Second Military Medical University. 2019;40(5):577–82.

34. Montenegro J, Klein MRST, Bregman R, Prado CM, Barreto Silva MI. Osteosarcopenia in patients with non-dialysis dependent chronic kidney disease. Clinical Nutrition. 2022 Jun 1;41(6):1218–27.

35. Xavier JS, Góes CR de, Borges MCC, Caramori JCT, Vogt BP. Handgrip Strength Thresholds are Associated With Malnutrition Inflammation Score (MIS) in Maintenance Hemodialysis Patients. Journal of Renal Nutrition. 2022 Nov 1;32(6):739–43.

36. Chiang JM, Kaysen GA, Segal M, Chertow GM, Delgado C, Johansen KL. Low testosterone is associated with frailty, muscle wasting and physical dysfunction among men receiving hemodialysis: A longitudinal analysis. Nephrology Dialysis Transplantation. 2019 May 1;34(5):802–10.

37. Zhou Q, Zhang H, Yin L, Li G, Liang W, Chen G. Characterization of the gut microbiota in hemodialysis patients with sarcopenia. Int Urol Nephrol. 2022 Aug 1;54(8):1899–906.

38. Zhou C, Lin X, Ma G, Yuan J, Zha Y. Increased Predialysis Extracellular to Intracellular Water Ratio Is Associated With Sarcopenia in Hemodialysis Patients. Journal of Renal Nutrition. 2023 Jan 1;33(1):157–64.

39. Yuenyongchaiwat K, Jongritthiporn S, Somsamarn K, Sukkho O, Pairojkittrakul S, Traitanon O. Depression and low physical activity are related to sarcopenia in hemodialysis: A single-center study. PeerJ. 2021 Jun 1;9.

40. Tsai PH, Yang HC, Lin C, Sung CC, Chu P, Hsu YJ. Association of serum phosphate with low handgrip strength in patients with advanced chronic kidney disease. Nutrients. 2021 Oct 1;13(10).

41. De Souza VA, Oliveira D, Barbosa SR, Corrêa JODA, Colugnati FAB, Mansur HN, et al. Sarcopenia in patients with chronic kidney disease not yet on dialysis: Analysis of the prevalence and associated factors. PLoS One. 2017 Apr 1;12(4).

42. Yoshimura Y, Wakabayashi H, Nagano F, Bise T, Shimazu S, Shiraishi A. Elevated Creatinine-Based Estimated Glomerular Filtration Rate is Associated with Increased Risk of Sarcopenia, Dysphagia, and Reduced Functional Recovery after Stroke. Journal of Stroke and Cerebrovascular Diseases. 2021 Feb 1;30(2).

43. Yanishi M, Tsukaguchi H, Kimura Y, Koito Y, Yoshida K, Seo M, et al. Evaluation of physical activity in sarcopenic conditions of kidney transplantation recipients. Int Urol Nephrol. 2017 Oct 1;49(10):1779–84.

44. Yoowannakul S, Tangvoraphonkchai K, Vongsanim S, Mohamed A, Davenport A. Differences in the prevalence of sarcopenia in haemodialysis patients: the effects of gender and ethnicity. Journal of Human Nutrition and Dietetics. 2018 Oct 1;31(5):689–96.

45. Yildirim S, Colak T, Bayraktar N, Sezer S. Evaluation of Dynapenia and Sarcopenia and Their Associations With Serum Insulin-Like Growth Factor-1 Levels in Renal Transplant Recipients. Journal of Renal Nutrition. 2022 May 1;32(3):354–62.

46. Yoowannakul S, Tangvoraphonkchai K, Davenport A. The prevalence of muscle wasting (sarcopenia) in peritoneal dialysis patients varies with ethnicity due to differences in muscle mass measured by bioimpedance. Eur J Clin Nutr. 2018 Mar 1;72(3):381–7.

47. Sánchez-Tocino ML, González-Parra E, Miranda Serrano B, Gracia-Iguacel C, De-Alba-Penãranda AM, López-González A, et al. Evaluation of the impact of an intradialytic exercise programme on sarcopaenia in very elderly haemodialysis patients. Clin Kidney J. 2022 Aug 1;15(8):1514–23.

48. Yasar E, Tek NA, Tekbudak MY, Yurtdaş G, Gülbahar Ö, Uyar GÖ, et al. The Relationship Between Myostatin, Inflammatory Markers, and Sarcopenia in Patients With Chronic Kidney Disease. Journal of Renal Nutrition. 2022 Nov 1;32(6):677–84.

49. Wilkinson T, Miksza J, Baker L, Lightfoot C, Watson E, Yates T, et al. MO023SARCOPENIA, CHRONIC KIDNEY DISEASE AND RISK OF MORTALITY: FINDINGS FROM 426,839 INDIVIDUALS IN THE UK BIOBANK. Nephrology Dialysis Transplantation. 2020 Jun 1;35(Supplement_3).

50. Widajanti N, Soelistijo S, Hadi U, Thaha M, Aditiawardana, Widodo, et al. Association between Sarcopenia and Insulin-Like Growth Factor-1, Myostatin, and Insulin Resistance in Elderly Patients Undergoing Hemodialysis. J Aging Res. 2022;2022.

51. Wilkinson TJ, Nixon DGD, Richler-Potts D, Neale J, Song Y, Smith AC. Identification of the most clinically useful skeletal muscle mass indices pertinent to sarcopenia and physical performance in chronic kidney disease. Nephrology. 2020 Jun 1;25(6):467–74.

52. Wilkinson TJ, Gore EF, Vadaszy N, Nixon DGD, Watson EL, Smith AC. Utility of Ultrasound as a Valid and Accurate Diagnostic Tool for Sarcopenia. Journal of Ultrasound in Medicine. 2021 Mar 1;40(3):457–67.

53. Wang M, Liu L, Shen X, Li Y, He Q. Assessing lean tissue by bioelectrical impedance analysis pre hemodialysis underestimates the prevalence of sarcopenia in maintenance hemodialysis patients. Eur J Clin Nutr. 2021 Sep 1;75(9):1407–13.

54. Wang L, Luo Q, Zhu B, Zhou F. Relation of Serum 25-Hydroxyvitamin D Status with Skeletal Muscle Mass and Grip Strength in Patients on Peritoneal Dialysis [Internet]. Vol. 65, J Nutr Sci Vitaminol. 2019. Available from: www.wma.net/en/30publications/10policies/b3/index.

55. Umakanthan M, Li JW, Sud K, Duque G, Guilfoyle D, Cho K, et al. Prevalence and factors associated with sarcopenia in patients on maintenance dialysis in australia—a single centre, cross-sectional study. Nutrients. 2021 Sep 1;13(9).

56. Ulgen C, Ozturk I, Sahin M, Guzel FB, Oguz A, Altunoren O, et al. The amount of skeletal muscle mass is associated with arterial stiffness in hemodialysis patients. Therapeutic Apheresis and Dialysis. 2023 Feb 1;27(1):24–30.

57. Song YR, Kim JK, Lee HS, Kim SG, Choi EK. Serum levels of protein carbonyl, a marker of oxidative stress, are associated with overhydration, sarcopenia and mortality in hemodialysis patients. BMC Nephrol. 2020 Jul 16;21(1).

58. Son HE, Ryu JY, Lee K, Choi Y Il, Kim MS, Park I, et al. The importance of muscle mass in predicting intradialytic hypotension in patients undergoing maintenance hemodialysis. Kidney Res Clin Pract. 2022 Sep 1;41(5):611–22.

59. Slee A, McKeaveney C, Adamson G, Davenport A, Farrington K, Fouque D, et al. Estimating the Prevalence of Muscle Wasting, Weakness, and Sarcopenia in Hemodialysis Patients. Journal of Renal Nutrition. 2020 Jul 1;30(4):313–21.

60. da Silva MZC, Vogt BP, Reis NS do C, Caramori JCT. Update of the European consensus on sarcopenia: what has changed in diagnosis and prevalence in peritoneal dialysis? Eur J Clin Nutr. 2019 Aug 1;73(8):1209–11.

61. Saitoh M, Ogawa M, Kondo H, Suga K, Takahashi T, Itoh H, et al. Sarcopenic obesity and its association with frailty and protein-energy wasting in hemodialysis patients: Preliminary data from a single center in Japan. Ren Replace Ther. 2019 Nov 27;5(1).

62. Rosa CSC, Ribeiro HS, Vogt BP, Sakkas GK, Monteiro HL. Sarcopenia diagnosis in patients receiving hemodialysis: Agreement among different consensuses. Nutrition in Clinical Practice. 2022 Dec 1;37(6):1348–55.

63. Pérez-Sáez MJ, Dávalos-Yerovi V, Redondo-Pachón D, Arias-Cabrales CE, Faura A, Bach A, et al. Frailty in kidney transplant candidates: a comparison between physical frailty phenotype and FRAIL scales. J Nephrol. 2022;

64. Ren H, Gong D, Jia F, Xu B, Liu Z. Sarcopenia in patients undergoing maintenance hemodialysis: Incidence rate, risk factors and its effect on survival risk. Ren Fail. 2016 Mar 15;38(3):364–71.

65. Reis JMS, Alves LS, Vogt BP. According to Revised EWGSOP Sarcopenia Consensus Cut-Off Points, Low Physical Function Is Associated With Nutritional Status and Quality of Life in Maintenance Hemodialysis Patients. Journal of Renal Nutrition. 2022 Jul 1;32(4):469–75.

66. Ozkayar N, Altun B, Halil M, Kuyumcu ME, Arik G, Yesil Y, et al. Evaluation of sarcopenia in renal transplant recipients. Nephrourol Mon. 2014;6(4).

67. Nanmoku K, Kawabata N, Kinoshita Y, Shinzato T, Kubo T, Shimizu T, et al. Deterioration of presarcopenia and its risk factors following kidney transplantation. Clin Exp Nephrol. 2020 Apr 1;24(4):379–83.

68. Mori K, Nishide K, Okuno S, Shoji T, Emoto M, Tsuda A, et al. Impact of diabetes on sarcopenia and mortality in patients undergoing hemodialysis. BMC Nephrol. 2019 Mar 28;20(1).

69. Moreno-Gonzalez R, Corbella X, Mattace-Raso F, Tap L, Sieber C, Freiberger E, et al. Prevalence of sarcopenia in community-dwelling older adults using the updated EWGSOP2 definition according to kidney function and albuminuria. BMC Geriatr. 2020 Oct 2;20.

70. Miyazaki S, Iino N, Koda R, Narita I, Kaneko Y. Brain-derived neurotrophic factor is associated with sarcopenia and frailty in Japanese hemodialysis patients. Geriatr Gerontol Int. 2021 Jan 1;21(1):27–33.

71. Menna Barreto APM, Barreto Silva MI, Pontes KSDS, Costa MS Da, Rosina KTDC, Souza E, et al. Sarcopenia and its components in adult renal transplant recipients: prevalence and association with body adiposity. British Journal of Nutrition. 2019 Dec 28;122(12):1386–97.

72. Mattera M, Veronese N, Aucella F, Tegola L La, Testini V, De Guio F, et al. Prevalence and Risk Factors for Sarcopenia in Chronic Kidney Disease Patients Undergoing Dialysis: A Cross-Sectional Study.

73. Medeiros MC, Rocha N, Bandeira E, Dantas I, Chaves C, Oliveira M, et al. Serum Sclerostin, Body Composition, and Sarcopenia in Hemodialysis Patients with Diabetes. Int J Nephrol. 2020;2020.

74. Matsuzawa R, Yamamoto S, Suzuki Y, Imamura K, Harada M, Matsunaga A, et al. The clinical applicability of ultrasound technique for diagnosis of sarcopenia in hemodialysis patients. Clinical Nutrition. 2021 Mar 1;40(3):1161–7.

75. Matei A, Bilha SC, Constantinescu D, Pavel-Tanasa M, Cianga P, Covic A, et al. Body composition, adipokines, FGF23-Klotho and bone in kidney transplantation: Is there a link? J Nephrol. 2022 Jan 1;35(1):293–304.

76. Martins CA, da Cunha França AKT, Dias RSC, de Oliveira Costa RC, Lemos APL, dos Santos AM, et al. Prevalence of sarcopenia in kidney transplants and their association with determinant factors of muscle homeostasis. Rev Assoc Med Bras. 2020 Sep 1;66(9):1235–40.

77. Marini ACB, Perez DRS, Fleuri JA, Pimentel GD. SARC-F Is Better Correlated with Muscle Function Indicators Than Muscle Mass in Older Hemodialysis Patients. Journal of Nutrition, Health and Aging. 2020;

78. de Oliveira EM, da Silva RP, de Lemos M da CC, Burgos MGP de A, Costa DMN, Maio R. Frequency of sarcopenia, cachexia, and associated factors in patients with chronic kidney disease in dialysis treatment. Nutr Hosp. 2020 Nov 1;37(6):1157–65.

79. Lin YL, Wang CH, Tsai JP, Chen CT, Chen YH, Hung SC, et al. A Comparison of SARC-F, Calf Circumference, and Their Combination for Sarcopenia Screening among Patients Undergoing Peritoneal Dialysis. Nutrients. 2022 Mar 1;14(5).

80. Lin YL, Liou HH, Wang CH, Lai YH, Kuo CH, Chen SY, et al. Impact of sarcopenia and its diagnostic criteria on hospitalization and mortality in chronic hemodialysis patients: A 3-year longitudinal study. Journal of the Formosan Medical Association. 2020 Jul 1;119(7):1219–29.

81. Lin YL, Wang CH, Lai YH, Kuo CH, Syu RJ, Hsu BG. Negative correlation between leptin serum levels and sarcopenia in hemodialysis patients. Int J Clin Exp Pathol [Internet]. 2018;11(3):1715–23. Available from: http://www.ncbi.nlm.nih.gov/pubmed/31938275

82. Lin YL, Wang CH, Chang IC, Hsu BG. A Novel Application of Serum Creatinine and Cystatin C to Predict Sarcopenia in Advanced CKD. Front Nutr. 2022 Feb 25;9.

83. Lin TY, Wu MY, Chen HS, Hung SC, Lim PS. Development and validation of a multifrequency bioimpedance spectroscopy equation to predict appendicular skeletal muscle mass in hemodialysis patients. Clinical Nutrition. 2021 May 1;40(5):3288–95.

84. Lin YL, Liou HH, Lai YH, Wang CH, Kuo CH, Chen SY, et al. Decreased serum fatty acid binding protein 4 concentrations are associated with sarcopenia in chronic hemodialysis patients. Clinica Chimica Acta. 2018 Oct 1;485:113–8.

85. Lee H, Kim K, Ahn J, Lee DR, Lee JH, Hwang SD. Association of nutritional status with osteoporosis, sarcopenia, and cognitive impairment in patients on hemodialysis. Asia Pac J Clin Nutr. 2020;29(4):712–23.

86. Limirio LS, Santos HO, dos Reis AS, de Oliveira EP. (Dis) Agreement between the first and the recent European consensus on definition and diagnosis for sarcopenia in kidney transplant patients. Eur J Clin Nutr [Internet]. 2020 Jul 25;74(7):1104–8. Available from: https://www.nature.com/articles/s41430-019-0535-5

87. Li C, Chen L, He L, Zhang Y, Chen H, Liu Y, et al. Study on the relationship between sarcopenia and its components and anorexia in elderly maintenance haemodialysis patients. Nurs Open. 2022 Mar 1;9(2):1096–104.

88. Lee YH, Kim JS, Jung SW, Hwang HS, Moon JY, Jeong KH, et al. Gait speed and handgrip strength as predictors of all-cause mortality and cardiovascular events in hemodialysis patients. BMC Nephrol. 2020 May 6;21(1).

89. Kusunoki H, Tsuji S, Kusukawa T, Wada Y, Tamaki K, Nagai K, et al. Relationships between cystatin C- and creatinine-based eGFR in Japanese rural community- dwelling older adults with sarcopenia. Clin Exp Nephrol. 2021 Mar 1;25(3):231–9.

90. Lamarca F, Carrero JJ, Rodrigues JCD, Bigogno FG, Fetter RL, Avesani CM. Prevalence of sarcopenia in elderly maintenance hemodialysis patients: The impact of different diagnostic criteria. J Nutr Health Aging [Internet]. 2014 Aug 28;18(7):710–7. Available from: http://link.springer.com/10.1007/s12603-014-0505-5

91. Lai S, Muscaritoli M, Andreozzi P, Sgreccia A, De Leo S, Mazzaferro S, et al. Sarcopenia and cardiovascular risk indices in patients with chronic kidney disease on conservative and replacement therapy. Nutrition. 2019 Jun 1;62:108–14.

92. Kosoku A, Uchida J, Nishide S, Kabei K, Shimada H, Iwai T, et al. Association of sarcopenia with phase angle and body mass index in kidney transplant recipients. Sci Rep. 2020 Dec 1;10(1).

93. Kobayashi H, Takahashi M, Fukutomi M, Oba Y, Funayama H, Kario K. The long-term prognostic factors in hemodialysis patients with acute coronary syndrome: perspectives from sarcopenia and malnutrition. Heart Vessels. 2021 Sep 1;36(9):1275–82.

94. Kono K, Moriyama Y, Yabe H, Hara A, Ishida T, Yamada T, et al. Relationship between malnutrition and possible sarcopenia in the AWGS 2019 consensus affecting mortality in hemodialysis patients: a prospective cohort study. BMC Nephrol. 2021 Dec 1;22(1).

95. Kurajoh M, Mori K, Miyabe M, Matsufuji S, Kizu A, Tsujimoto Y, et al. Xanthine Oxidoreductase Inhibitor Use Associated With Reduced Risk of Sarcopenia and Severe Sarcopenia in Patients Undergoing Hemodialysis. Front Med (Lausanne). 2022 Feb 7;9.

96. Koito Y, Yanishi M, Kimura Y, Tsukaguchi H, Kinoshita H, Matsuda T. Serum Brain-Derived Neurotrophic Factor and Myostatin Levels Are Associated With Skeletal Muscle Mass in Kidney Transplant Recipients. Transplant Proc. 2021 Jul 1;53(6):1939–44.

97. Kittiskulnam P, Nitesnoppakul M, Metta K, Suteparuk S, Praditpornsilpa K, Eiam-Ong S. Alterations of body composition patterns in pre-dialysis chronic kidney disease patients. Int Urol Nephrol. 2021 Jan 1;53(1):137–45.

98. Kittiskulnam P, Chertow GM, Carrero JJ, Delgado C, Kaysen GA, Johansen KL. Sarcopenia and its individual criteria are associated, in part, with mortality among patients on hemodialysis. Kidney Int. 2017 Jul 1;92(1):238–47.

99. Kim JK, Choi SR, Choi MJ, Kim SG, Lee YK, Noh JW, et al. Prevalence of and factors associated with sarcopenia in elderly patients with end-stage renal disease. Clinical Nutrition. 2014 Feb;33(1):64–8.

100. Kim JC, Do JY, Cho JH, Kang SH. Comparison of appendicular lean mass indices for predicting physical performance in Korean hemodialysis patients: A cross-sectional study. Medicine (United States). 2021 Dec 10;100(49).

101. Kim JK, Kim SG, Oh JE, Lee YK, Noh JW, Kim HJ, et al. Impact of sarcopenia on long-term mortality and cardiovascular events in patients undergoing hemodialysis. Korean Journal of Internal Medicine. 2019 May 1;34(3):599–607.

102. Kamijo Y, Kanda E, Ishibashi Y, Yoshida M. Sarcopenia and frailty in PD: Impact on mortality, malnutrition, and inflammation. Peritoneal Dialysis International. 2018 Nov 1;38(6):447–54.

103. Khoo S Bin, Lin YL, Ho GJ, Lee MC, Hsu BG. Association of endothelial dysfunction with sarcopenia and muscle function in a relatively young cohort of kidney transplant recipients. PeerJ. 2021 Nov 22;9.

104. Isoyama N, Qureshi AR, Avesani CM, Lindholm B, Bárány P, Heimbürger O, et al. Comparative associations of muscle mass and muscle strength with mortality in dialysis patients. Clinical Journal of the American Society of Nephrology. 2014;9(10):1720–8.

105. Ishimura E, Okuno S, Nakatani S, Mori K, Miyawaki J, Okazaki H, et al. Significant Association of Diabetes With Mortality of Chronic Hemodialysis Patients, Independent of the Presence of Obesity, Sarcopenia, and Sarcopenic Obesity. Journal of Renal Nutrition. 2022 Jan 1;32(1):94–101.

106. Ishikawa S, Naito S, Iimori S, Takahashi D, Zeniya M, Sato H, et al. Loop diuretics are associated with greater risk of sarcopenia in patients with non-dialysis-dependent chronic kidney disease. PLoS One. 2018 Feb 1;13(2).

107. Imamura K, Yamamoto S, Suzuki Y, Matsuzawa R, Harada M, Yoshikoshi S, et al. Limitations of SARC-F as a Screening Tool for Sarcopenia in Patients on Hemodialysis. Nephron. 2022 Jan 1;146(1):32–9.

108. Hyun YY, Lee KB, Rhee EJ, Park CY, Chang Y, Ryu S. Chronic kidney disease and high eGFR according to body composition phenotype in adults with normal BMI. Nutrition, Metabolism and Cardiovascular Diseases. 2016 Dec 1;26(12):1088–95.

109. Tangvoraphonkchai K, Hung R, Sadeghi-Alavijeh O, Davenport A. Differences in Prevalence of Muscle Weakness (Sarcopenia) in Haemodialysis Patients Determined by Hand Grip Strength Due to Variation in Guideline Definitions of Sarcopenia. Nutrition in Clinical Practice. 2018 Apr 1;33(2):255–60.

110. Furtado EVH, Alves JDA, Santos EJF, Nunes LCR, Galvão JC, Nunes RF, et al. Sarcopenia and inflammation in patients undergoing hemodialysis. Nutr Hosp. 2020 Jul 1;37(4):855–62.

111. Hayashi H, Izumiya Y, Hayashi O, Ichii M, Tsujimoto Y, Yoshiyama M. Dynapenia is an independent predictor of cardio-cerebrovascular events in patients undergoing hemodialysis. Heart Vessels. 2022 Jun 1;37(6):1066–74.

112. M. B. Bustamante Hernández, Garduño García JDJ, Montenegro Morales LP, Camarillo Romero EDS, Huitrón Bravo G, Camarillo Romero MDS. Sarcopenia, neuropatía periférica y enfermedad arterial periférica en pacientes con diabetes mellitus sometidos a hemodiálisis. Revista de nefrologia, dialisis y trasplante [Internet]. 2021;41(3):151–8. Available from: www.renal.org.ar

113. Guida B, Maro M Di, Lauro M Di, Lauro T Di, Trio R, Santillo M, et al. Identification of sarcopenia and dynapenia in CKD predialysis patients with EGWSOP2 criteria: An observational, cross-sectional study. Nutrition. 2020 Oct 1;78.

114. Giglio J, Kamimura MA, Lamarca F, Rodrigues J, Santin F, Avesani CM. Association of Sarcopenia With Nutritional Parameters, Quality of Life, Hospitalization, and Mortality Rates of Elderly Patients on Hemodialysis. Journal of Renal Nutrition. 2018 May 1;28(3):197–207.

115. P. Duarte M, Ribeiro HS, Almeida LS, Baião VM, Inda‐Filho A, Avesani CM, et al. SARC‐F and SARC‐CalF are associated with sarcopenia traits in hemodialysis patients. Nutrition in Clinical Practice [Internet]. 2022 Dec 7;37(6):1356–65. Available from: https://aspenjournals.onlinelibrary.wiley.com/doi/10.1002/ncp.10819

116. Ferreira MF, Böhlke M, Pauletto MB, Frühauf IR, Gonzalez MC. Sarcopenia diagnosis using different criteria as a predictor of early mortality in patients undergoing hemodialysis. Nutrition. 2022 Mar 1;95.

117. Fernandes JFR, Barreto Silva MI, Loivos CP, Menna Barreto APM, Meira V da S, Kaiser SE, et al. Obstructive sleep apnea in non-dialyzed chronic kidney disease patients: Association with body adiposity and sarcopenia. Nutrition. 2019 Jan 1;57:282–9.

118. Fan Z, Guo Y, Zhong XY. Circulating Cell-Free Mitochondrial DNA: A Potential Blood-Based Biomarker for Sarcopenia in Patients Undergoing Maintenance Hemodialysis. Medical Science Monitor. 2021;28.

119. Ding Y, Chang L, Zhang H, Wang S. Predictive value of phase angle in sarcopenia in patients on maintenance hemodialysis. Nutrition. 2022 Feb 1;94.

120. Dubey AK, Sahoo J, Vairappan B, Parameswaran S, PS P. Prevalence and determinants of sarcopenia in Indian patients with chronic kidney disease stage 3 & 4. Osteoporos Sarcopenia. 2021 Dec;7(4):153–8.

121. dos Reis AS, Limirio LS, Santos HO, de Oliveira EP. Intake of polyunsaturated fatty acids and ω-3 are protective factors for sarcopenia in kidney transplant patients. Nutrition. 2021 Jan 1;81.

122. Do JY, Seo JH, Kang SH. Validation of the SARC-F for Assessing Sarcopenia in Patients on Peritoneal Dialysis. Journal of Renal Nutrition. 2022 May 1;32(3):341–6.

123. Do JY, Kang SH. Association Between Peritonitis and Low Muscle Mass in Peritoneal Dialysis Patients. Journal of Renal Nutrition. 2020 Jul 1;30(4):341–6.

124. Dierkes J, Dahl H, Lervaag Welland N, Sandnes K, Sæle K, Sekse I, et al. High rates of central obesity and sarcopenia in CKD irrespective of renal replacement therapy - An observational cross-sectional study. BMC Nephrol. 2018 Oct 11;19(1).

125. de Oliveira Matos B, da Costa Rosa CS, Ribeiro HS, Marcos NM, Losilla MPR, Monteiro HL, et al. Obesity phenotypes are, in part, associated with physical activity in diabetic hemodialysis patients. Int Urol Nephrol. 2022 Jul 1;54(7):1751–9.

126. de Amorim GJ, Calado CKM, Souza de Oliveira BC, Araujo RPO, Filgueira TO, de Sousa Fernandes MS, et al. Sarcopenia in Non-Dialysis Chronic Kidney Disease Patients: Prevalence and Associated Factors. Front Med (Lausanne). 2022 Apr 7;9.

127. D’alessandro C, Piccoli GB, Barsotti M, Tassi S, Giannese D, Morganti R, et al. Prevalence and correlates of sarcopenia among elderly CKD outpatients on tertiary care. Nutrients. 2018 Dec 10;10(12).

128. Davenport A. Comparison of frailty, sarcopenia and protein energy wasting in a contemporary peritoneal dialysis cohort. Peritoneal Dialysis International. 2022 Nov 1;42(6):571–7.

129. Cheng D, Zhang Q, Wang Z, Li J, Jian G, Wang N. Association Between Sarcopenia and Its Components and Dependency in Activities of Daily Living in Patients on Hemodialysis. Journal of Renal Nutrition. 2021 Jul 1;31(4):397–402.

130. Cha R hui, Kang SH, Han MY, An WS, Kim SH, Kim JC. Effects of AST-120 on muscle health and quality of life in chronic kidney disease patients: results of RECOVERY study. J Cachexia Sarcopenia Muscle. 2022 Feb 1;13(1):397–408.

131. Caldiroli L, Armelloni S, Eskander A, Messa P, Rizzo V, Margiotta E, et al. Association between the uremic toxins indoxyl-sulfate and p-cresyl-sulfate with sarcopenia and malnutrition in elderly patients with advanced chronic kidney disease. Exp Gerontol. 2021 May 1;147.

132. Chan W, Chin SH, Whittaker AC, Jones D, Kaur O, Bosch JA, et al. The Associations of Muscle Strength, Muscle Mass, and Adiposity With Clinical Outcomes and Quality of Life in Prevalent Kidney Transplant Recipients. Journal of Renal Nutrition. 2019 Nov 1;29(6):536–47.

133. Bellafronte NT, Sizoto GR, Vega-Piris L, Chiarello PG, Cuadrado GB. Bed-side measures for diagnosis of low muscle mass, sarcopenia, obesity, and sarcopenic obesity in patients with chronic kidney disease under non-dialysis-dependent, dialysis dependent and kidney transplant therapy. PLoS One. 2020 Nov 1;15(11 November).

134. An JN, Kim JK, Lee HS, Kim SG, Kim HJ, Song YR. Late stage 3 chronic kidney disease is an independent risk factor for sarcopenia, but not proteinuria. Sci Rep. 2021 Dec 1;11(1).

135. Bataille S, Serveaux M, Carreno E, Pedinielli N, Darmon P, Robert A. The diagnosis of sarcopenia is mainly driven by muscle mass in hemodialysis patients. Clinical Nutrition. 2017 Dec 1;36(6):1654–60.

136. As’habi A, Najafi I, Tabibi H, Hedayati M. Prevalence of Sarcopenia and Dynapenia and Their Determinants in Iranian Peritoneal Dialysis Patients [Internet]. Vol. 12, Iranian Journal of Kidney Diseases |. 2018. Available from: www.ijkd.org

137. Abro A, Delicata LA, Vongsanim S, Davenport A. Differences in the prevalence of sarcopenia in peritoneal dialysis patients using hand grip strength and appendicular lean mass: Depends upon guideline definitions. Eur J Clin Nutr. 2018 Jul 1;72(7):993–9.

138. Androga L, Sharma D, Amodu A, Abramowitz MK. Sarcopenia, Obesity, and Mortality in US Adults With and Without Chronic Kidney Disease. Kidney Int Rep. 2017;2(2):201–11.

139. Abdala R, Elena del Valle E, Negri AL, Bridoux P, Paganti LG, Bravo M, et al. Sarcopenia in hemodialysis patients from Buenos Aires, Argentina. Osteoporos Sarcopenia. 2021 Jun;7(2):75–80.

140. Alston H, Burns A, Davenport A. Loss of appendicular muscle mass in haemodialysis patients is associated with increased self-reported depression, anxiety and lower general health scores. Nephrology. 2018 Jun 1;23(6):546–51.

**Supporting Table 1**. Cutoff values for the consensus definition of sarcopenia.

| **Consensus** | **Appendicular skeletal muscle mass** | | | | **Muscle strength^#^** | | **Physical performance** | **Operational definition** |
| --- | --- | --- | --- | --- | --- | --- | --- | --- |
|  | **DXA** | | **Bioimpedance analysis** | |  | |  |  |
|  | **Absolute** | **Adjusted** | **Absolute** | **Adjusted** | **Men** | **Women** |  |  |
| **EWGSOP** | N/A | Women: <5.5 kg/m^2^  Men: <7.26 kg/m^2^ | Women  Severe sarcopenia ≤5.75kg/m^2^  Moderate sarcopenia 5.76−6.75kg/m^2^  Men  Severe Sarcopenia ≤8.50kg/m^2^  Moderate sarcopenia 8.51−10.75kg/m^2^ | Women 6.42 kg/m^2^  Men 8.87 kg/m^2^ | <30 kg | <20 kg | Gait speed ≤0.8 m/s;  SPPB  score ≤8 | ↓ muscle  +  ↓ strength  or  ↓ performance |
| **IWGS** | N/A | Women: <5.67 kg/m^2^  Men: <7.23 kg/m^2^ | N/A | Women: <5.67 kg/m^2^  Men: <7.23 kg/m^2^ | N/A | | Gait speed <1.0 m/s | ↓ performance  +  ↓ muscle |
| **FNIH** | Women: <15.02 kg  Men: <19.75 kg | Women: <0.512 kg/BMI  Men: <0.789 kg/BMI | N/A | N/A | <26 kg  <1.0 kg/BMI | <16 kg  <0.56 kg/BMI | Gait speed ≤0.8 m/s | ↓ performance  +  ↓ strength  or  ↓ muscle |
| **AWGS** | N/A | Women: <5.4 kg/m^2^  Men: <7.0 kg/m^2^ | N/A | Women: <5.7 kg/m^2^  Men: <7.0  kg/m^2^ | <26 kg | <18 kg | Gait speed ≤0.8 m/s | ↓ strength  or  ↓ performance  +  ↓ muscle |
| **EWGSOP2** | Women: <15.0 kg  Men: <20.0 kg | Women: <5.5 kg/m^2^  Men: <7.0 kg/m^2^ | Women <15 kg  Men <20kg | Women <5.5 kg/m^2^  Men <7.0 kg/m^2^ | <27 kg | <16 kg | Gait speed ≤0.8 m/s;  SPPB  score ≤8;  TUG  ≥20 seg;  400 m ≥6 min | ↓ strength  +  ↓ muscle  +  ↓ performance (for severity) |
|  |  |  |  |  | >15 seg 5-rep sit-to-stand | |  |  |
| **AWGS 2019** | N/A | Women: <5.4 kg/m^2^  Men: <7.0 kg/m^2^ | N/A | Women: <5.7 kg/m^2^  Men: <7.0  kg/m^2^ | <28 kg | <18 kg | Gait speed <1.0 m/s;  SPPB  score ≤9  5-rep sit-to-stand  ≥12 seg | ↓ strength  or  ↓ performance  +  ↓ muscle |

* DXA, dual-energy x-ray absorptiometry values; ^#^ handgrip strength values.

N/A, not applicable; BMI, body mass index; SPPB, short physical performance battery; TUG, timed up and go; EWGSOP, European Working Group on Sarcopenia in Older People; EWGSOP2, revised EWGSOP; FNIH, Foundation for the National Institutes of Health Sarcopenia Project; AWGS, Asian Work Group on Sarcopenia; AWGS 2019, revised AWGS; IWGS, International Working Group on Sarcopenia.

**Supporting Table 2.** Characteristics of the studies included in the systematic review.

| **First author** | **Year** | **Country** | **Study Design** | **Sample size**  **(n)** | **Men (n)** | **Mean age (years)** | **CKD stage** | **Prevalence of Sarcopenia*** | | **Consensus** | **Muscle mass device** | **Muscle strength device** | **Performance method** |
| --- | --- | --- | --- | --- | --- | --- | --- | --- | --- | --- | --- | --- | --- |
|  |  |  |  |  |  |  |  | **Absolute (n)** | **Relative (%)** |  |  |  |  |
| Abdala et al. | 2021 | Argentina | Cross-sectional | 100 | 60 | 55.6 | Hemodialysis | 16 | 16,0 | EWGSOP2 | DEXA | Handgrip | Gait speed |
| Abro et al. | 2018 | United Kingdom | Cross-sectional | 155 | 95 | 63 | Peritoneal dialysis | 24 | 15,5 | FNIH | BIA | Handgrip | Gait speed |
| Adrian Slee et al. | 2019 | United Kingdom | Cross-sectional | 87 | 63 | 65.9 | Hemodialysis | 38 | 43,7 | EWGSOP | BIA | Handgrip | − |
| Aline S. dos Reis et al. | 2021 | Brazil | Cross-sectional | 125 | 85 | 48 | Kidney transplant | 23 | 18,4 | EWGSOP2 | BIA | Handgrip | Gait speed |
| Alston et al | 2018 | United Kingdom | Cross-sectional | 113 | 84 | 64.9 | Hemodialysis | − | − | EWGSOP | BIA | − | − |
| Amorim et al. | 2022 | Brazil | Cross-sectional | 139 | 65 | 57 | Non-dialysis | 29 | 20,9 | EWGSOP2 | BIA | Handgrip | Gait speed |
| Androga et al. | 2017 | United State of America | Cross-sectional | 1101 | 514 | − | Non-dialysis | − | − | EWGSOP | DEXA | − | − |
| As'habi et al. | 2018 | Iran | Cross-sectional | 79 | 35 |  | Peritoneal dialysis | 9 | 11,4 | EWGSOP | BIA | Handgrip | Gait speed |
| Baltac et al. | 2022 | Turkey | Prospective cohort | 106 | 53 | 57.4 | Dialysis (HD + PD) | 50 | 47,2 | EWGSOP | BIA | Handgrip | − |
| Barreto et al. | 2019 | Brazil | Cross-sectional | 185 | 106 | 50 | Kidney transplant | 32 | 17,3 | EWGSOP | DEXA | Handgrip | Gait speed |
| Bataille et al. | 2017 | France | Cross-sectional | 111 | 65 | 77.5 | Hemodialysis | 35 | 31,5 | EWGSOP | BIA | Handgrip | − |
| Bellafronte et al. | 2020 | Brazil | Cross-sectional | 265 | 136 | 48 | CKD-grouped | 18 | 6,8 | EWGSOP2 | DEXA | Handgrip | − |
| Cai et al. | 2022 | China | Cross-sectional | 615 | 381 | 60.07 | Hemodialysis | 102 | 16,6 | AWGS2 | BIA | Handgrip | Gait speed |
| Caldiroli et al. | 2021 | Italy | Cross-sectional | 99 | 66 | 81 | Non-dialysis | 23 | 23,2 | EWGSOP2 | − | Handgrip | Gait speed |
| Chan et al. | 2019 | United Kingdom | Prospective cohort | 128 | 72 | 49 | Kidney transplant | 37 | 28,9 | EWGSOP | BIA | Handgrip | − |
| Chao li et al. | 2021 | China | Cross-sectional | 112 | 68 | 70 | Hemodialysis | 59 | 52,7 | AWGS2 | DEXA | Handgrip | Gait speed |
| Chaomin Zhou et al. | 2022 | China | Cross-sectional | 3196 | 1277 | 55 | Hemodialysis | 1156 | 36,2 | AWGS | BIA | Handgrip | − |
| Chen et al. | 2022 | China | Cross-sectional | 233 | 82 | 69.32 | Non-dialysis | 43 | 18,5 | AWGS2 | BIA | Handgrip | Gait speed |
| Chen et al. b | 2022 | China | Cross-sectional | 142 | 75 | 54.05 | Hemodialysis | 40 | 28,2 | AWGS2 | BIA | Handgrip | Gait speed |
| Cheng et al. | 2020 | China | Cross-sectional | 238 | 161 | 60.9 | Hemodialysis | 117 | 49,2 | AWGS2 | BIA | Handgrip | Gait speed |
| Côrrea et al. | 2023 | Brazil | Prospective cohort | 247 | 150 | 66.6 | Hemodialysis | 65 | 26,3 | EWGSOP2 | DEXA | Handgrip | − |
| Costa et al. | 2023 | Brazil | Prospective cohort | 170 | 100 | 49.5 | Kidney transplant | 11 | 6,5 | EWGSOP2 | DEXA | Handgrip | − |
| D’Alessandro et al. | 2018 | Italy | Cross-sectional | 80 | 80 | 73.7 | Non-dialysis | 54 | 67,5 | EWGSOP | BIA | Handgrip | TUG |
| Dahl et al. | 2023 | Norway | Prospective cohort | 139 | 100 | 61 | CKD-grouped | 14 | 10,1 | EWGSOP2 | BIA | Handgrip | − |
| Davenport. | 2020 | United Kingdom | Retrospective cohort | 368 | 226 | 60.9 | Peritoneal dialysis | 41 | 11,1 | EWGSOP2 | BIA | Handgrip | − |
| Dierkes et al. | 2018 | Norway | Cross-sectional | 208 | 147 | − | CKD-grouped | 74 | 35,6 | EWGSOP | BIA | Handgrip | − |
| Du et al. | 2022 | China | Cross-sectional | 589 | 385 | 53.8 | Hemodialysis | 101 | 17,1 | AWGS | BIA | Handgrip | Gait speed |
| Duarte et al. | 2022 | Brazil | Cross-sectional | 30 | 17 | 57.2 | Hemodialysis | 2 | 6,7 | EWGSOP2 | BIA | Handgrip | Gait speed |
| Dubey et al. | 2021 | India | Cross-sectional | 188 | 134 | 50.2 | Non-dialysis | − | − | AWGS | DEXA | − | − |
| Elder et al. | 2023 | Australia | Prospective cohort | 77 | 44 | − | Hemodialysis | 33 | 42,9 | EWGSOP2 | BIA | Handgrip | TUG |
| Fan et al. | 2022 | China | Cross-sectional | 105 | 59 | 59.78 | Hemodialysis | 23 | 21,9 | AWGS2 | DEXA | Handgrip | Gait speed |
| Fernandes et al. | 2018 | Brazil | Cross-sectional | 73 | 42 | 62.88 | Non-dialysis | 9 | 12,3 | EWGSOP | DEXA | Handgrip | Gait speed |
| Ferreira et al. | 2022 | Brazil | Cross-sectional | 127 | 72 | − | Hemodialysis | − | − | EWGSOP | − | Handgrip | Gait speed |
| Fu et al. | 2023 | China | Cross-sectional | 76 | 51 | 61.8 | Hemodialysis | 39 | 51,3 | AWGS2 | DEXA | Handgrip | SPPB |
| Giglio et al. | 2018 | Brazil | Cross-sectional | 170 | 111 | 70.6 | Hemodialysis | 62 | 36,5 | EWGSOP | DEXA | Handgrip | − |
| Guida et al. | 2020 | Italy | Cross-sectional | 85 | 55 | 65 | Non-dialysis | 6 | 7,1 | EWGSOP2 | BIA | Handgrip | − |
| Heeryong Lee et al. | 2020 | Republic of Korea | Cross-sectional | 131 | 71 | 66.2 | Hemodialysis | 13 | 9,9 | AWGS | BIA | Handgrip | − |
| Hernández et al. | 2021 | Mexico | Cross-sectional | 33 | 20 | 60 | Hemodialysis | 11 | 33,3 | EWGSOP2 | BIA | Handgrip | Gait speed |
| Hiroya Hayashi et al. | 2022 | Japan | Retrospective cohort | 244 | 172 | − | Hemodialysis | 94 | 38,5 | AWGS2 | DEXA | Handgrip | Gait speed |
| Hortegal et al. | 2020 | Brazil | Cross-sectional | 209 | 124 | 51.9 | Hemodialysis | 61 | 29,2 | EWGSOP2 | BIA | Handgrip | Gait speed |
| Hou et al. | 2023 | Taiwan | Retrospective cohort | 58 | 40 | 65.64 | CKD-grouped | 11 | 19,0 | AWGS2 | DEXA | Handgrip | − |
| Hyun et al. | 2016 | Republic of Korea | Cross-sectional | 249 | 132 | 72.2 | Non-dialysis | − | − | IWGS | DEXA | − | − |
| Hyung Eun Son et al. | 2022 | Republic of Korea | Cross-sectional | 177 | 87 | 59.5 | Hemodialysis | 4 | 2,3 | AWGS2 | BIA | Handgrip | − |
| Imamura et al. | 2021 | Japan | Cross-sectional | 179 | 104 | 66.5 | Hemodialysis | 49 | 27,4 | AWGS2 | BIA | Handgrip | Gait speed |
| Ishikawa et al. | 2018 | Japan | Cross-sectional | 260 | 169 | 76 | Non-dialysis | 65 | 25,0 | AWGS | DEXA | Handgrip | Gait speed |
| Ishimura et al. | 2022 | Japan | Retrospective cohort | 308 | 185 | 58 | Hemodialysis | 83 | 26,9 | AWGS2 | DEXA | Handgrip | − |
| Isoyama et al. | 2014 | Sweden | Cross-sectional | 330 | 203 | 53 | Hemodialysis | 68 | 20,6 | EWGSOP | DEXA | Handgrip | − |
| Janet M. Chiang et al. | 2019 | United State of America | Prospective cohort | 440 | 440 | 56.1 | Hemodialysis | 75 | 17,0 | FNIH | BIA | Handgrip | Gait speed |
| Jauwerissa et al. | 2023 | Indonesia | Cross-sectional | 96 | 48 | 50.82 | Hemodialysis | 52 | 54,2 | AWGS2 | BIA | Handgrip | Gait speed |
| Joao Marcos Soares Reis et al. | 2022 | Brazil | Cross-sectional | 77 | 50 | 55.3 | Hemodialysis | − | − | EWGSOP2 |  | Handgrip | SPPB |
| Jun Chul Kim et al. | 2021 | Republic of Korea | Cross-sectional | 84 | 44 | 55.6 | Hemodialysis | 13 | 15,5 | AWGS | DEXA | − | − |
| Jun Young Do et al. a | 2019 | Republic of Korea | Retrospective cohort | 230 | 109 | − | Peritoneal dialysis | − | − | FNIH | BIA | − | − |
| Jun Young Do et al. b | 2021 | Republic of Korea | Cross-sectional | 127 | 67 | 55.5 | Peritoneal dialysis | 10 | 7,9 | AWGS | BIA | Handgrip | − |
| Jung Nam An et al. | 2021 | Republic of Korea | Prospective cohort | 892 | 523 | 66 | Non-dialysis | 189 | 21,2 | AWGS2 | BIA | Handgrip | − |
| Kakita et al. | 2022 | Japan | Cross-sectional | 356 | 227 | 71.3 | Hemodialysis | 142 | 39,9 | AWGS2 | BIA | Handgrip | SPPB |
| Kamijo et al. | 2018 | Japan | Prospective cohort | 119 | 84 | 66.8 | Peritoneal dialysis | 13 | 10,9 | AWGS | BIA | Handgrip | Gait speed |
| Kang et al. | 2022 | Republic of Korea | Retrospective cohort | 199 | 113 | 55.7 | Peritoneal dialysis | 64 | 32,2 | AWGS | DEXA | Handgrip | − |
| Kim et al. | 2014 | Republic of Korea | Cross-sectional | 95 | 54 | 63.9 | Hemodialysis | 32 | 33,7 | EWGSOP | BIA | Handgrip | − |
| Kim et al. b | 2019 | Republic of Korea | Prospective cohort | 142 | 81 | 59.8 | Hemodialysis | 47 | 33,1 | EWGSOP | BIA | Handgrip | − |
| Kittiskulnam et al. | 2021 | Thailand | Cross-sectional | 103 | 62 | 61.3 | Non-dialysis | − | − | AWGS2 | BIA | − | − |
| Kittiskulnam et al. b | 2017 | United State of America | Prospective cohort | 645 | 378 | 56.7 | Hemodialysis | 102 | 15,8 | EWGSOP | Ultrasound | Handgrip | Gait speed |
| Kobayashi et al. | 2021 | Japan | Cross-sectional | 58 | 45 | 64.5 | Hemodialysis | − | − | AWGS | BIA | − | − |
| Kono et al. | 2021 | Japan | Prospective cohort | 635 | 355 | 70.5 | Hemodialysis | − | − | AWGS2 | − | Handgrip | − |
| Kosoku et al. | 2020 | Japan | Cross-sectional | 210 | 122 | 55 | Kidney transplant | 24 | 11,4 | AWGS | BIA | Handgrip | Gait speed |
| Kosoku et al. b | 2023 | Japan | Cross-sectional | 100 | 63 | 67 | Kidney transplant | 16 | 16,0 | AWGS2 | BIA | Handgrip | Gait speed |
| Kurajoh et al | 2022 | Japan | Cross-sectional | 296 | 203 | 68 | Hemodialysis | 125 | 42,2 | AWGS2 | DEXA | Handgrip | STS (5 times) |
| Kusunoki et al. | 2020 | Japan | Cross-sectional | 225 | 79 | 75.8 | Non-dialysis | 24 | 10,7 | AWGS2 | BIA | Handgrip | Gait speed |
| Lai et al. | 2019 | Italy | Prospective cohort | 77 | 43 | 69.6 | CKD-grouped | 38 | 49,4 | EWGSOP | BIA | Handgrip | − |
| Lamarca et al. | 2014 | Brazil | Cross-sectional | 102 | 75 | 70.7 | Hemodialysis | 65 | 63,7 | EWGSOP | DEXA | Handgrip | − |
| Lee et al. | 2020 | Republic of Korea | Cross-sectional | 150 | 96 | 65 | Non-dialysis | 14 | 9,3 | AWGS2 | BIA | Handgrip | Gait speed |
| Limirio et al. | 2019 | Brazil | Cross-sectional | 127 | 87 | 47.7 | Kidney transplant | 64 | 50,4 | EWGSOP | BIA | Handgrip | SPPB |
| Marini et al. | 2020 | Brazil | Cross-sectional | 95 | 59 | 60.9 | Hemodialysis | − | − | EWGSOP2 | BIA | Handgrip | Gait speed |
| Martins et al. | 2020 | Brazil | Cross-sectional | 83 | 48 | 48.8 | Kidney transplant | 16 | 19,3 | EWGSOP | DEXA | Handgrip | Gait speed |
| Matei et al. | 2021 | Romania | Cross-sectional | 59 | 30 | 44 | Kidney transplant | − | − | IWGS | DEXA | − | − |
| Matos et al. | 2022 | Brazil | Cross-sectional | 84 | 46 | 63.5 | Hemodialysis | 8 | 9,5 | EWGSOP2 | BIA | Handgrip | − |
| Matsuzawa et al. | 2020 | Japan | Cross-sectional | 58 | 36 | 77.5 | Hemodialysis | 34 | 58,6 | AWGS2 | BIA | Handgrip | Gait speed |
| Mattera el al. | 2021 | Italy | Cross-sectional | 77 | 49 | 62.7 | Hemodialysis | − | − | EWGSOP | DEXA | − | − |
| Medeiros et al. | 2020 | Brazil | Cross-sectional | 92 | 58 | 63.3 | Hemodialysis | 22 | 23,9 | EWGSOP | BIA | Handgrip | Gait speed |
| Minmin Wang et al. | 2021 | China | Cross-sectional | 87 | 61 | 66.6 | Hemodialysis | 20 | 23,0 | AWGS | BIA | Handgrip | Gait speed |
| Miyazaki et al. | 2020 | Japan | Cross-sectional | 20 | 14 | 76.5 | Hemodialysis | 11 | 55,0 | AWGS2 | DEXA | Handgrip | Gait speed |
| Montenegro et al. | 2022 | Brazil | Cross-sectional | 257 | 148 | 64.8 | Non-dialysis | 2 | 0,8 | EWGSOP2 | DEXA | Handgrip |  |
| Moreno-Gonzalez et al. | 2020 | Multinational | Cross-sectional | 494 | − | − | Non-dialysis | 56 | 11,3 | EWGSOP2 | BIA | Handgrip | SPPB |
| Mori et al. | 2019 | Japan | Prospective cohort | 308 | 185 | − | Hemodialysis | 124 | 40,3 | AWGS | DEXA | Handgrip | − |
| Nanmoku et al. | 2019 | Japan | Prospective cohort | 80 | 50 | 44.6 | Dialysis (HD + PD) | − | − | AWGS | BIA | − | − |
| Oliveira et al. | 2020 | Brazil | Cross-sectional | 66 | 28 | 53.15 | Dialysis (HD + PD) | 39 | 59,1 | EWGSOP | BIA | Handgrip | TUG |
| Ozkayar et al. | 2014 | Turkey | Cross-sectional | 166 | 98 | 37.9 | Kidney transplant | 34 | 20,5 | EWGSOP | BIA | Handgrip | − |
| Pérez-Sáez et al. | 2021 | Spain | Cross-sectional | 451 | 308 | 60.9 | Dialysis (HD + PD) | 100 | 22,2 | EWGSOP2 | BIA | Handgrip | Gait speed |
| Ping-Huang Tsai et al. | 2021 | Taiwan | Cross-sectional | 134 | 93 | 65.34 | Non-dialysis | 9 | 6,7 | AWGS2 | BIA | Handgrip | Gait speed |
| Plytzanopoulou et al. | 2022 | Greece | Cross-sectional | 130 | 89 | 66.1 | Hemodialysis | − | − | EWGSOP2 | − | Handgrip | − |
| Qifan Zhou et al. | 2021 | China | Cross-sectional | 60 | 34 | − | Hemodialysis | 30 | 50,0 | AWGS2 | DEXA | Handgrip | Gait speed |
| Ran-hui Cha. | 2021 | Republic of Korea | Cross-sectional | 150 | 97 | 65 | Non-dialysis | 24 | 16,0 | AWGS2 | BIA | Handgrip | Gait speed |
| Rao et al. | 2022 | India | Cross-sectional | 117 | 88 | 55.7 | Non-dialysis | 34 | 29,1 | AWGS2 | BIA | Handgrip | Gait speed |
| Ren et al. | 2016 | China | Cross-sectional | 131 | 80 | 49.4 | Hemodialysis | 18 | 13,7 | EWGSOP | BIA | Handgrip | − |
| Rosa et al. | 2021 | Brazil | Cross-sectional | 67 | 43 | 54.6 | Hemodialysis | 8 | 11,9 | EWGSOP | DEXA | Handgrip | − |
| Saitoh et al. | 2019 | Japan | Cross-sectional | 117 | 76 | 64 | Hemodialysis | 35 | 29,9 | AWGS | BIA | Handgrip | Gait speed |
| Saliha Yildirim et al. | 2022 | Turkey | Cross-sectional | 120 | 60 | 40.4 | Kidney transplant | 4 | 3,3 | FNIH | BIA | Handgrip | Gait speed |
| Sánchez-Tocino et al. a | 2022 | Spain | Cross-sectional | 60 | 41 | 81.85 | Hemodialysis | 20 | 33,3 | EWGSOP2 | BIA | Handgrip | Gait speed |
| Sánchez-Tocino et al. b | 2022 | Spain | Prospective cohort | 60 | 41 | 81.85 | Hemodialysis | 24 | 40,0 | EWGSOP2 | BIA | Handgrip | SPPB |
| Shin et al. | 2022 | Republic of Korea | Cross-sectional | 149 | 97 | 65 | Non-dialysis | 14 | 9,4 | AWGS2 | BIA | Handgrip | Gait speed |
| Silva et al. | 2019 | Brazil | Cross-sectional | 50 | 24 | 55.74 | Peritoneal dialysis | 5 | 10,0 | EWGSOP | DEXA | Handgrip | Gait speed |
| Siok-Bin Khoo et al. | 2021 | Taiwan | Cross-sectional | 95 | 46 | 45.2 | Kidney transplant | 11 | 11,6 | AWGS2 | BIA | Handgrip | Gait speed |
| Song et al. | 2022 | China | Cross-sectional | 598 | 243 | 72.4 | CKD-grouped | 179 | 29,9 | AWGS2 | BIA | Handgrip | Gait speed |
| Souza et al. | 2017 | Brazil | Cross-sectional | 100 | 41 | 73.59 | Non-dialysis | 29 | 29,0 | FNIH | DEXA | Handgrip | Gait speed |
| Tangvoraphonkchai et al. | 2018 | United Kingdom | Cross-sectional | 459 | 282 | 66.9 | Hemodialysis | − | − | EWGSOP | − | Handgrip | − |
| Ting-Yun Lin et al. | 2020 | Taiwan | Cross-sectional | 263 | 144 | 60 | Hemodialysis | 19 | 7,2 | AWGS2 | BIA | Handgrip | − |
| Ulgen et al. | 2022 | Turkey | Cross-sectional | 79 | 41 | 52.5 | Hemodialysis | 25 | 31,6 | EWGSOP2 | BIA | Handgrip | − |
| Umakanthan et al. | 2021 | Australia | Cross-sectional | 39 | 28 | 69 | Dialysis (HD + PD) | 7 | 17,9 | EWGSOP | BIA | Handgrip | TUG |
| Wang et al. | 2023 | China | Cross-sectional | 130 | 72 | 54.12 | Hemodialysis | 36 | 27,7 | AWGS2 | BIA | Handgrip | Gait speed |
| Wang et al. | 2019 | China | Cross-sectional | 113 | 65 | 58.7 | Peritoneal dialysis | − | − | AWGS | BIA | Handgrip | − |
| Wen Du et al. | 2023 | China | Cross-sectional | 125 | 68 | 59.4 | Hemodialysis | 39 | 31,2 | EWGSOP2 | DEXA | Handgrip | Gait speed |
| Widajanti et al. | 2022 | Indonesia | Cross-sectional | 40 | 25 | 64 | Hemodialysis | 33 | 82,5 | EWGSOP2 | BIA | Handgrip | Gait speed |
| Wilkinson et al. a | 2021 | United Kingdom | Prospective cohort | 8740 | 4055 | 62.8 | Non-dialysis | 66 | 0,8 | FNIH | BIA | Handgrip | − |
| Wilkinson et al. b | 2021 | United Kingdom | Cross-sectional | 113 | 54 | 62 | Non-dialysis | − | − | FNIH | BIA | − | − |
| Wilkinson et al. c | 2019 | United Kingdom | Cross-sectional | 72 | 46 | 55.7 | CKD-grouped | − | − | EWGSOP2 | DEXA | Handgrip | − |
| Wu et al. | 2023 | China | Cross-sectional | 105 | 43 | 54.2 | Kidney transplant | 33 | 31,4 | AWGS2 | BIA | Handgrip | STS (5 times) |
| Xavier et al. | 2022 | Brazil | Cross-sectional | 218 | 124 | 58.3 | Hemodialysis | − | − | EWGSOP |  | Handgrip | − |
| Xiang et al. | 2023 | China | Prospective cohort | 209 | 99 | 58.45 | Hemodialysis | 78 | 37,3 | AWGS2 | BIA | Handgrip | − |
| Xiao-tong et al. | 2019 | China | Cross-sectional | 101 | 58 | 64.48 | Hemodialysis | 51 | 50,5 | EWGSOP | BIA | Handgrip | Gait speed |
| Xie et al. | 2023 | China | Cross-sectional | 757 | 453 | 60.4 | Hemodialysis | 124 | 16,4 | AWGS2 | BIA | Handgrip | Gait speed |
| Yajima et al. | 2023 | Japan | Cross-sectional | 85 | 58 | 67.8 | Hemodialysis | 33 | 38,8 | AWGS2 | BIA | Handgrip | − |
| Yan Ding et al | 2022 | China | Cross-sectional | 346 | 213 | 58.17 | Hemodialysis | 113 | 32,7 | AWGS2 | BIA | Handgrip | Gait speed |
| Yang et al. | 2023 | China | Prospective cohort | 1117 | 654 | 56.8 | Hemodialysis | 141 | 12,6 | AWGS2 | BIA | Handgrip | − |
| Yanishi et al. | 2017 | Republic of Korea | Cross-sectional | 58 | 42 | 46.6 | Kidney transplant | 12 | 20,7 | AWGS | DEXA | Handgrip | Gait speed |
| Yasar et al. | 2022 | Turkey | Cross-sectional | 130 | 72 | 46.8 | CKD-grouped | 37 | 28,5 | EWGSOP2 | BIA | Handgrip | − |
| Yoowannakul et al. a | 2017 | United Kingdom | Cross-sectional | 434 | 239 | 55.3 | Peritoneal dialysis | − | − | EWGSOP | BIA | − | − |
| Yoowannakul et al. b | 2018 | Japan | Cross-sectional | 600 | 373 | 66.3 | Hemodialysis | 228 | 38,0 | EWGSOP | BIA | Handgrip | − |
| Yoshikoshi et al. | 2022 | Japan | Retrospective cohort | 616 | 375 | 65.4 | Hemodialysis | − | − | AWGS2 | − | Handgrip | − |
| Yoshimura et al. | 2021 | Japan | Retrospective cohort | 333 | 203 | 73.5 | Non-dialysis | 152 | 45,6 | AWGS2 | BIA | Handgrip | − |
| Young Rim Song et al. | 2020 | Republic of Korea | Cross-sectional | 88 | 50 | 60.6 | Hemodialysis | − | − | EWGSOP | DEXA | Handgrip | − |
| Yu Ho Lee et al. | 2020 | Republic of Korea | Prospective cohort | 277 | 183 | − | Hemodialysis | − | − | AWGS | − | Handgrip | Gait speed |
| Yuenyongchaiwat et al. | 2021 | Thailand | Cross-sectional | 104 | 54 | 59.74 | Hemodialysis | 34 | 32,7 | AWGS2 | BIA | Handgrip | Gait speed |
| Yu-Li Lin et al. a | 2018 | Taiwan | Cross-sectional | 120 | 63 | 63.3 | Hemodialysis | 20 | 16,7 | EWGSOP | BIA | Handgrip | Gait speed |
| Yu-Li Lin et al. b | 2018 | Taiwan | Cross-sectional | 76 | 43 | 60.39 | Hemodialysis | 8 | 10,5 | AWGS | BIA | Handgrip | Gait speed |
| Yu-Li Lin et al. c | 2020 | Taiwan | Cross-sectional | 126 | 65 | 63.2 | Hemodialysis | 17 | 13,5 | EWGSOP | BIA | Handgrip | Gait speed |
| Yu-Li Lin et al. d | 2022 | Taiwan | Cross-sectional | 186 | 86 | 57.5 | Peritoneal dialysis | 71 | 38,2 | AWGS2 | BIA | Handgrip | Gait speed |
| Yu-Li Lin et al. e | 2022 | Taiwan | Cross-sectional | 297 | 169 | 68.8 | Non-dialysis | 60 | 20,2 | AWGS2 | DEXA | Handgrip | SPPB |
| Yuya Koito et al. | 2021 | Japan | Cross-sectional | 40 | 29 | 52.1 | Kidney transplant |  |  | AWGS2 | DEXA | − | − |
| Zhang et al. | 2022 | China | Prospective cohort | 158 | 78 | 56 | Hemodialysis | 46 | 29,1 | AWGS2 | BIA | Handgrip | Gait speed |
| Zhen Fan et al. | 2022 | China | Cross-sectional | 105 | 59 | − | Hemodialysis | 23 | 21,9 | AWGS2 | DEXA | Handgrip | Gait speed |
| Zhou et al. | 2023 | China | Cross-sectional | 2743 | 1603 | 55 | Hemodialysis | 429 | 15,6 | AWGS | BIA | Handgrip | − |

* sarcopenia was defined as recommended by the consensuses as low physical function + low muscle mass.

6MTW, 6-minute walking test; AWGS, Asian Working Group for Sarcopenia; BIA, bioelectrical impedance analysis, CKD, chronic kidney disease; DEXA, dual-energy X-ray absorptiometry; EWGSOP, European Working Group on Sarcopenia in Older People; FNIH, Foundation for the National Institutes of Health Sarcopenia; GS, gait speed; HD, hemodialysis; HGS, handgrip strength; IWGS, International Working Group on Sarcopenia; ND, non-dialysis; PD, peritoneal dialysis; SPPB, short physical performance battery; STS, sit-to-stand test; TUG, Timed Up and Go test; Tx, kidney transplant.

**Supporting Table 3:** JBI tool for methodological quality assessment of the included studies.

| **First author** | **Year** | **Item 1** | **Item 2** | **Item 3** | **Item 4** | **Item 5** | **Item 6** | **Item 7** | **Item 8** | **Item 9** | **Total** |
| --- | --- | --- | --- | --- | --- | --- | --- | --- | --- | --- | --- |
| Abdala et al. | 2021 | 1 | 0 | 1 | 1 | 1 | 1 | 1 | 0 | NA | 6 |
| Abro et al. | 2018 | 1 | 0 | 1 | 0 | 1 | 1 | 1 | 0 | NA | 5 |
| Adrian Slee et al. | 2019 | 1 | 0 | 0 | 1 | 1 | 1 | 1 | 0 | NA | 5 |
| Aline S. dos Reis et al. | 2021 | 1 | 0 | 1 | 1 | 1 | 1 | 1 | 0 | NA | 6 |
| Alston et al. | 2018 | 1 | 0 | 1 | 1 | 1 | 1 | 1 | 0 | NA | 6 |
| Amorim et al. | 2022 | 1 | 0 | 1 | 1 | 1 | 1 | 1 | 0 | NA | 6 |
| Androga et al. | 2017 | 1 | 1 | 1 | 1 | 1 | 1 | 1 | 0 | NA | 7 |
| As'habi et al. | 2018 | 1 | 0 | 0 | 0 | 1 | 1 | 1 | 0 | NA | 4 |
| Baltac et al. | 2022 | 1 | 0 | 1 | 1 | 1 | 1 | 1 | 0 | NA | 6 |
| Barreto et al. | 2019 | 1 | 0 | 1 | 1 | 1 | 1 | 1 | 0 | NA | 6 |
| Bataille et al. | 2017 | 1 | 0 | 1 | 1 | 1 | 1 | 1 | 0 | NA | 6 |
| Bellafronte et al. | 2020 | 1 | 0 | 1 | 1 | 1 | 1 | 1 | 0 | NA | 6 |
| Cai et al. | 2022 | 1 | 0 | 1 | 1 | 1 | 1 | 1 | 0 | NA | 6 |
| Caldiroli et al. | 2021 | 1 | 0 | 0 | 1 | 1 | 1 | 1 | 0 | NA | 5 |
| Chan et al. | 2019 | 1 | 0 | 1 | 1 | 1 | 1 | 1 | 0 | NA | 6 |
| Chao li et al. | 2021 | 1 | 0 | 1 | 1 | 1 | 1 | 1 | 0 | NA | 6 |
| Chaomin Zhou et al. | 2022 | 1 | 0 | 1 | 1 | 1 | 1 | 1 | 0 | NA | 6 |
| Chen et al. | 2022 | 1 | 1 | 1 | 1 | 1 | 1 | 1 | 0 | NA | 7 |
| Chen et al. b | 2022 | 1 | 0 | 1 | 1 | 1 | 1 | 1 | 0 | NA | 6 |
| Cheng et al. | 2020 | 1 | 0 | 1 | 1 | 1 | 1 | 1 | 0 | NA | 6 |
| Côrrea et al. | 2023 | 1 | 0 | 1 | 1 | 1 | 1 | 1 | 0 | NA | 6 |
| Costa et al. | 2023 | 1 | 0 | 1 | 1 | 1 | 1 | 1 | 1 | NA | 7 |
| D’Alessandro et al. | 2018 | 1 | 0 | 0 | 1 | 1 | 1 | 1 | 0 | NA | 5 |
| Dahl et al. | 2023 | 1 | 0 | 1 | 1 | 1 | 1 | 1 | 0 | NA | 6 |
| Davenport. | 2020 | 1 | 0 | 1 | 0 | 1 | 1 | 1 | 0 | NA | 5 |
| Dierkes et al. | 2018 | 1 | 0 | 1 | 0 | 1 | 1 | 1 | 0 | NA | 5 |
| Du et al. | 2022 | 1 | 0 | 1 | 1 | 1 | 1 | 1 | 0 | NA | 6 |
| Duarte et al. | 2022 | 1 | 0 | 0 | 1 | 1 | 1 | 1 | 0 | NA | 5 |
| Dubey et al. | 2021 | 1 | 0 | 1 | 1 | 1 | 1 | 1 | 0 | NA | 6 |
| Elder et al. | 2023 | 1 | 0 | 0 | 1 | 1 | 1 | 1 | 0 | NA | 5 |
| Fan et al. | 2022 | 1 | 0 | 1 | 1 | 1 | 1 | 1 | 0 | NA | 6 |
| Fernandes et al. | 2018 | 1 | 0 | 0 | 1 | 1 | 1 | 1 | 0 | NA | 5 |
| Ferreira et al. | 2022 | 1 | 0 | 1 | 0 | 1 | 1 | 1 | 0 | NA | 5 |
| Fu et al. | 2023 | 1 | 0 | 1 | 1 | 1 | 1 | 1 | 0 | NA | 6 |
| Giglio et al. | 2018 | 1 | 1 | 1 | 1 | 1 | 1 | 1 | 0 | NA | 7 |
| Guida et al. | 2020 | 1 | 0 | 0 | 1 | 1 | 1 | 1 | 0 | NA | 5 |
| Heeryong Lee et al. | 2020 | 1 | 0 | 1 | 1 | 1 | 1 | 1 | 0 | NA | 6 |
| Hernández et al. | 2021 | 1 | 0 | 0 | 1 | 1 | 1 | 1 | 0 | NA | 5 |
| Hiroya Hayashi et al. | 2022 | 1 | 0 | 1 | 1 | 1 | 1 | 1 | 0 | NA | 6 |
| Hortegal et al. | 2020 | 1 | 0 | 1 | 1 | 1 | 1 | 1 | 0 | NA | 6 |
| Hou et al. | 2023 | 1 | 0 | 1 | 1 | 1 | 1 | 1 | 0 | NA | 6 |
| Hyun et al. | 2016 | 1 | 1 | 1 | 1 | 1 | 1 | 1 | 0 | NA | 7 |
| Hyung Eun Son et al. | 2022 | 1 | 0 | 1 | 1 | 1 | 1 | 1 | 0 | NA | 6 |
| Imamura et al. | 2021 | 1 | 0 | 1 | 1 | 1 | 1 | 1 | 0 | NA | 6 |
| Ishikawa et al. | 2018 | 1 | 0 | 1 | 1 | 1 | 1 | 1 | 0 | NA | 6 |
| Ishimura et al. | 2022 | 1 | 0 | 1 | 1 | 1 | 1 | 1 | 0 | NA | 6 |
| Isoyama et al. | 2014 | 1 | 0 | 1 | 1 | 1 | 1 | 1 | 0 | NA | 6 |
| Janet M. Chiang et al. | 2019 | 1 | 0 | 1 | 1 | 1 | 1 | 1 | 0 | NA | 6 |
| Jauwerissa et al. | 2023 | 1 | 0 | 0 | 1 | 1 | 1 | 1 | 0 | NA | 5 |
| Joao Marcos Soares Reis et al. | 2022 | 1 | 0 | 0 | 1 | 1 | 1 | 1 | 0 | NA | 5 |
| Jun Chul Kim et al. | 2021 | 1 | 0 | 0 | 1 | 1 | 1 | 1 | 0 | NA | 5 |
| Jun Young Do et al. a | 2019 | 1 | 0 | 1 | 0 | 1 | 1 | 1 | 0 | NA | 5 |
| Jun Young Do et al. b | 2021 | 1 | 0 | 1 | 1 | 1 | 1 | 1 | 0 | NA | 6 |
| Jung Nam An et al. | 2021 | 1 | 0 | 1 | 1 | 1 | 1 | 1 | 0 | NA | 6 |
| Kakita et al. | 2022 | 1 | 0 | 1 | 1 | 1 | 1 | 1 | 0 | NA | 6 |
| Kamijo et al. | 2018 | 1 | 0 | 1 | 0 | 1 | 1 | 1 | 0 | NA | 5 |
| Kang et al. | 2022 | 1 | 0 | 1 | 1 | 1 | 1 | 1 | 0 | NA | 6 |
| Kim et al. | 2014 | 1 | 0 | 0 | 1 | 1 | 1 | 1 | 0 | NA | 5 |
| Kim et al. b | 2019 | 1 | 0 | 1 | 1 | 1 | 1 | 1 | 0 | NA | 6 |
| Kittiskulnam et al. | 2021 | 1 | 0 | 1 | 1 | 1 | 1 | 1 | 0 | NA | 6 |
| Kittiskulnam et al. b | 2017 | 1 | 1 | 1 | 1 | 1 | 1 | 1 | 0 | NA | 7 |
| Kobayashi et al. | 2021 | 1 | 0 | 0 | 0 | 1 | 1 | 1 | 0 | NA | 4 |
| Kono et al. | 2021 | 1 | 0 | 1 | 0 | 1 | 1 | 1 | 0 | NA | 5 |
| Kosoku et al. | 2020 | 1 | 0 | 1 | 1 | 1 | 1 | 1 | 0 | NA | 6 |
| Kosoku et al. b | 2023 | 1 | 0 | 1 | 1 | 1 | 1 | 1 | 0 | NA | 6 |
| Kurajoh et al | 2022 | 1 | 0 | 1 | 1 | 1 | 1 | 1 | 0 | NA | 6 |
| Kusunoki et al. | 2020 | 1 | 0 | 1 | 1 | 1 | 1 | 1 | 0 | NA | 6 |
| Lai et al. | 2019 | 1 | 0 | 0 | 1 | 1 | 1 | 1 | 0 | NA | 5 |
| Lamarca et al. | 2014 | 1 | 0 | 1 | 1 | 1 | 1 | 1 | 0 | NA | 6 |
| Lee et al. | 2020 | 1 | 0 | 1 | 1 | 1 | 1 | 1 | 0 | NA | 6 |
| Limirio et al. | 2019 | 1 | 0 | 1 | 1 | 1 | 1 | 1 | 0 | NA | 6 |
| Marini et al. | 2020 | 1 | 0 | 0 | 1 | 1 | 1 | 1 | 0 | NA | 5 |
| Martins et al. | 2020 | 1 | 0 | 0 | 0 | 1 | 1 | 1 | 0 | NA | 4 |
| Matei et al. | 2021 | 1 | 0 | 0 | 1 | 1 | 1 | 1 | 0 | NA | 5 |
| Matos et al. | 2022 | 1 | 0 | 0 | 1 | 1 | 1 | 1 | 0 | NA | 5 |
| Matsuzawa et al. | 2020 | 1 | 0 | 1 | 1 | 1 | 1 | 1 | 0 | NA | 6 |
| Mattera el al. | 2021 | 1 | 0 | 0 | 1 | 1 | 1 | 1 | 0 | NA | 5 |
| Medeiros et al. | 2020 | 1 | 0 | 0 | 0 | 1 | 1 | 1 | 0 | NA | 4 |
| Minmin Wang et al. | 2021 | 1 | 0 | 0 | 1 | 1 | 1 | 1 | 0 | NA | 5 |
| Miyazaki et al. | 2020 | 1 | 0 | 0 | 1 | 1 | 1 | 1 | 0 | NA | 5 |
| Montenegro et al. | 2022 | 1 | 0 | 1 | 1 | 1 | 1 | 1 | 0 | NA | 6 |
| Moreno-Gonzalez et al. | 2020 | 0 | 1 | 1 | 0 | 1 | 1 | 1 | 0 | NA | 5 |
| Mori et al. | 2019 | 1 | 0 | 1 | 1 | 1 | 1 | 1 | 0 | NA | 6 |
| Nanmoku et al. | 2019 | 1 | 0 | 0 | 1 | 1 | 1 | 1 | 0 | NA | 5 |
| Oliveira et al. | 2020 | 1 | 0 | 0 | 0 | 1 | 1 | 1 | 0 | NA | 4 |
| Ozkayar et al. | 2014 | 1 | 0 | 1 | 1 | 1 | 1 | 1 | 0 | NA | 6 |
| Pérez-Sáez et al. | 2021 | 1 | 0 | 1 | 1 | 1 | 1 | 1 | 0 | NA | 6 |
| Ping-Huang Tsai et al. | 2021 | 1 | 0 | 1 | 1 | 1 | 1 | 1 | 0 | NA | 6 |
| Plytzanopoulou et al. | 2022 | 1 | 0 | 1 | 1 | 1 | 1 | 1 | 0 | NA | 6 |
| Qifan Zhou et al. | 2021 | 1 | 0 | 1 | 1 | 1 | 1 | 1 | 0 | NA | 6 |
| Ran-hui Cha. | 2021 | 1 | 0 | 1 | 1 | 1 | 1 | 1 | 0 | NA | 6 |
| Rao et al. | 2022 | 1 | 0 | 1 | 1 | 1 | 1 | 1 | 0 | NA | 6 |
| Ren et al. | 2016 | 1 | 0 | 1 | 1 | 1 | 1 | 1 | 0 | NA | 6 |
| Rosa et al. | 2021 | 1 | 0 | 0 | 0 | 1 | 1 | 1 | 0 | NA | 4 |
| Saitoh et al. | 2019 | 1 | 0 | 1 | 1 | 1 | 1 | 1 | 0 | NA | 6 |
| Saliha Yildirim et al. | 2022 | 1 | 0 | 1 | 1 | 1 | 1 | 1 | 0 | NA | 6 |
| Sánchez-Tocino et al. a | 2022 | 1 | 0 | 0 | 0 | 1 | 1 | 1 | 0 | NA | 4 |
| Sánchez-Tocino et al. b | 2022 | 1 | 0 | 0 | 1 | 1 | 1 | 1 | 0 | NA | 5 |
| Shin et al. | 2022 | 1 | 0 | 1 | 1 | 1 | 1 | 1 | 0 | NA | 6 |
| Silva et al. | 2019 | 1 | 0 | 0 | 1 | 1 | 1 | 1 | 0 | NA | 5 |
| Siok-Bin Khoo et al. | 2021 | 1 | 0 | 0 | 1 | 1 | 1 | 1 | 0 | NA | 5 |
| Song et al. | 2022 | 1 | 0 | 1 | 1 | 1 | 1 | 1 | 0 | NA | 6 |
| Souza et al. | 2017 | 1 | 0 | 1 | 1 | 1 | 1 | 1 | 0 | NA | 6 |
| Tangvoraphonkchai et al. | 2018 | 1 | 1 | 1 | 0 | 1 | 1 | 1 | 0 | NA | 6 |
| Ting-Yun Lin et al. | 2020 | 1 | 0 | 1 | 1 | 1 | 1 | 1 | 0 | NA | 6 |
| Ulgen et al. | 2022 | 1 | 0 | 0 | 1 | 1 | 1 | 1 | 0 | NA | 5 |
| Umakanthan et al. | 2021 | 1 | 0 | 0 | 1 | 1 | 1 | 1 | 0 | NA | 5 |
| Wang et al. | 2023 | 1 | 0 | 1 | 1 | 1 | 1 | 1 | 0 | NA | 6 |
| Wang et al. | 2019 | 1 | 0 | 1 | 1 | 1 | 1 | 1 | 0 | NA | 6 |
| Wen Du et al. | 2023 | 1 | 0 | 1 | 1 | 1 | 1 | 1 | 0 | NA | 6 |
| Widajanti et al. | 2022 | 1 | 0 | 0 | 1 | 1 | 1 | 1 | 0 | NA | 5 |
| Wilkinson et al. a | 2021 | 1 | 1 | 1 | 1 | 1 | 1 | 1 | 0 | NA | 7 |
| Wilkinson et al. b | 2021 | 1 | 0 | 1 | 1 | 1 | 1 | 1 | 0 | NA | 6 |
| Wilkinson et al. c | 2019 | 1 | 0 | 0 | 0 | 1 | 1 | 1 | 0 | NA | 4 |
| Wu et al. | 2023 | 1 | 0 | 1 | 1 | 1 | 1 | 1 | 0 | NA | 6 |
| Xavier et al. | 2022 | 1 | 0 | 1 | 1 | 1 | 1 | 1 | 0 | NA | 6 |
| Xiang et al. | 2023 | 1 | 0 | 1 | 1 | 1 | 1 | 1 | 0 | NA | 6 |
| Xiao-tong et al. | 2019 | 1 | 0 | 1 | 1 | 1 | 1 | 1 | 0 | NA | 6 |
| Xie et al. | 2023 | 1 | 0 | 1 | 1 | 1 | 1 | 1 | 0 | NA | 6 |
| Yajima et al. | 2023 | 1 | 0 | 0 | 1 | 1 | 1 | 1 | 0 | NA | 5 |
| Yan Ding et al | 2022 | 1 | 0 | 1 | 1 | 1 | 1 | 1 | 0 | NA | 6 |
| Yang et al. | 2023 | 1 | 1 | 1 | 1 | 1 | 1 | 1 | 0 | NA | 7 |
| Yanishi et al. | 2017 | 1 | 0 | 0 | 1 | 1 | 1 | 1 | 0 | NA | 5 |
| Yasar et al. | 2022 | 1 | 0 | 1 | 1 | 1 | 1 | 1 | 0 | NA | 6 |
| Yoowannakul et al. a | 2017 | 1 | 0 | 1 | 1 | 1 | 1 | 1 | 0 | NA | 6 |
| Yoowannakul et al. b | 2018 | 1 | 0 | 1 | 1 | 1 | 1 | 1 | 0 | NA | 6 |
| Yoshikoshi et al. | 2022 | 1 | 0 | 1 | 1 | 1 | 1 | 1 | 0 | NA | 6 |
| Yoshimura et al. | 2021 | 1 | 0 | 1 | 0 | 1 | 1 | 1 | 0 | NA | 5 |
| Young Rim Song et al. | 2020 | 1 | 0 | 0 | 1 | 1 | 1 | 1 | 0 | NA | 5 |
| Yu Ho Lee et al. | 2020 | 1 | 0 | 1 | 1 | 1 | 1 | 1 | 0 | NA | 6 |
| Yuenyongchaiwat et al. | 2021 | 1 | 0 | 1 | 1 | 1 | 1 | 1 | 0 | NA | 6 |
| Yu-Li Lin et al. a | 2018 | 1 | 0 | 1 | 0 | 1 | 1 | 1 | 0 | NA | 5 |
| Yu-Li Lin et al. b | 2018 | 1 | 0 | 0 | 1 | 1 | 1 | 1 | 0 | NA | 5 |
| Yu-Li Lin et al. c | 2020 | 1 | 0 | 1 | 1 | 1 | 1 | 1 | 0 | NA | 6 |
| Yu-Li Lin et al. d | 2022 | 1 | 0 | 1 | 1 | 1 | 1 | 1 | 0 | NA | 6 |
| Yu-Li Lin et al. e | 2022 | 1 | 0 | 1 | 1 | 1 | 1 | 1 | 0 | NA | 6 |
| Yuya Koito et al. | 2021 | 1 | 0 | 0 | 1 | 1 | 1 | 1 | 0 | NA | 5 |
| Zhang et al. | 2022 | 1 | 0 | 1 | 1 | 1 | 1 | 1 | 0 | NA | 6 |
| Zhen Fan et al. | 2022 | 1 | 0 | 1 | 1 | 1 | 1 | 1 | 0 | NA | 6 |
| Zhou et al. | 2023 | 1 | 0 | 1 | 1 | 1 | 1 | 1 | 0 | NA | 6 |

Item 1. Was the sample frame appropriate to address the target population?; Item 2. Were study participants recruited in an appropriate way?; Item 3. Was the sample size adequate?; Item 4. Were the study subjects and setting described in detail?; Item 5. Was data analysis conducted with sufficient coverage of the identified sample?; Item 6. Were valid methods used for the identification of the condition?; Item 7. Was the condition measured in a standard, reliable way for all participants?; Item 8. Was there appropriate statistical analysis?; Item 9. Was the response rate adequate, and if not, was the low response rate managed appropriately?

**Supporting Table 4.** Pooled prevalence of sarcopenia in patients with chronic kidney disease.

|  | Meta-analysis | | | | Heterogeneity | |
| --- | --- | --- | --- | --- | --- | --- |
|  | No. of studies | No. of events | No. of patients | Prevalence of sarcopenia, % (95% CI) | *I*^2^ (%) | *P*-value for subgroup difference |
| **Stages of CKD and KRT** | | | |  |  |  |
| Non-dialysis | 19 | 879 | 12,908 | 16.7 (8.9−26.3) | 99.0 | 0.33 |
| Hemodialysis | 63 | 4,775 | 18,190 | 28.8 (25.5−32.3) | 95.8 |  |
| Peritoneal dialysis | 8 | 237 | 1,283 | 16.3 (9.3−24.8) | 92.6 |  |
| Dialysis (HD + PD) | 4 | 196 | 662 | 35.8 (18.1−55.8) | 94.4 |  |
| Kidney transplant | 13 | 317 | 1,672 | 18.4 (12.3−25.3) | 91.6 |  |
| CKD-grouped (≥ 2 stages) | 7 | 371 | 1,475 | 24.0 (13.9−35.8) | 95.4 |  |
| Dialysis (all studies) | 75 | 5,208 | 20,135 | 27.7 (24.7−30.9) | 95.6 | 0.08 |
| Non-dialysis | 19 | 879 | 12,908 | 16.7 (8.9−26.3) | 99.0 |  |
| **Overall prevalence** | 114 | 6,775 | 36,190 | 24.5 (20.9−28.3) | 98.4 | <0.001 |

CKD, chronic kidney disease; HD, hemodialysis; PD, peritoneal dialysis.

**Supporting Table 5.** Prevalence of sarcopenia by Asia vs non-Asian countries.

|  | No. of studies | No. of patients | No. of cases | Prevalence of sarcopenia, % (95% CI) | *I*^2^ (%) | *P* |
| --- | --- | --- | --- | --- | --- | --- |
| Asian | 70 | 20,349 | 5,222 | 26.0 (22.9−29.2) | 95.9 | 0.74 |
| Non-Asian | 43 | 15,347 | 1,497 | 22.3 (15.9−29.4) | 98.6 |  |

**Supporting Table 6.** Prevalence of sarcopenia stratified by gender according to the stages of CKD and KRT.

| Stages of CKD and dialysis modality | No. of studies | No. of patients | Cases | Prevalence of sarcopenia, % (95% CI) | *I*^2^ (%) |
| --- | --- | --- | --- | --- | --- |
| ***Men*** |  |  |  |  |  |
| Non-dialysis | 7 | 618 | 150 | 20.7 (8.6−36.3) | 94.6 |
| Hemodialysis | 43 | 5,955 | 1,471 | 28.4 (24.4−32.5) | 91.0 |
| Peritoneal dialysis | 2 | 119 | 19 | 15.6 (9.5−22.8) | − |
| Dialysis (HD + PD) | 4 | 417 | 100 | 31.3 (13.2−52.8) | 91.1 |
| Kidney transplant | 8 | 602 | 96 | 15.4 (10.4−21.2) | 68.7 |
| CKD-grouped (≥ 2 stages) | 2 | 179 | 37 | 14.8 (9.9−20.5) | − |
| **Total** | 66 | 7,890 | 1,873 | 25.8 (22.4−29.3) | 91.5 |
| ***Women*** |  |  |  |  |  |
| Non-dialysis | 6 | 521 | 105 | 27.0 (11.5−45.9) | 94.7 |
| Hemodialysis | 42 | 3,710 | 952 | 27.4 (21.8−33.4) | 93.1 |
| Peritoneal dialysis | 2 | 79 | 3 | 3.6 (0.3−9.4) | − |
| Dialysis (HD + PD) | 4 | 245 | 96 | 43.6 (28.3−59.5) | 77.7 |
| Kidney transplant | 7 | 402 | 77 | 18.3 (11.4−26.3) | 71.0 |
| CKD-grouped (≥ 2 stages) | 2 | 163 | 19 | 11.2 (6.7−16.7) | − |
| **Total** | 63 | 5,120 | 1,252 | 25.7 (21.3−30.4) | 92.3 |

CKD, chronic kidney disease; HD, hemodialysis; PD, peritoneal dialysis.


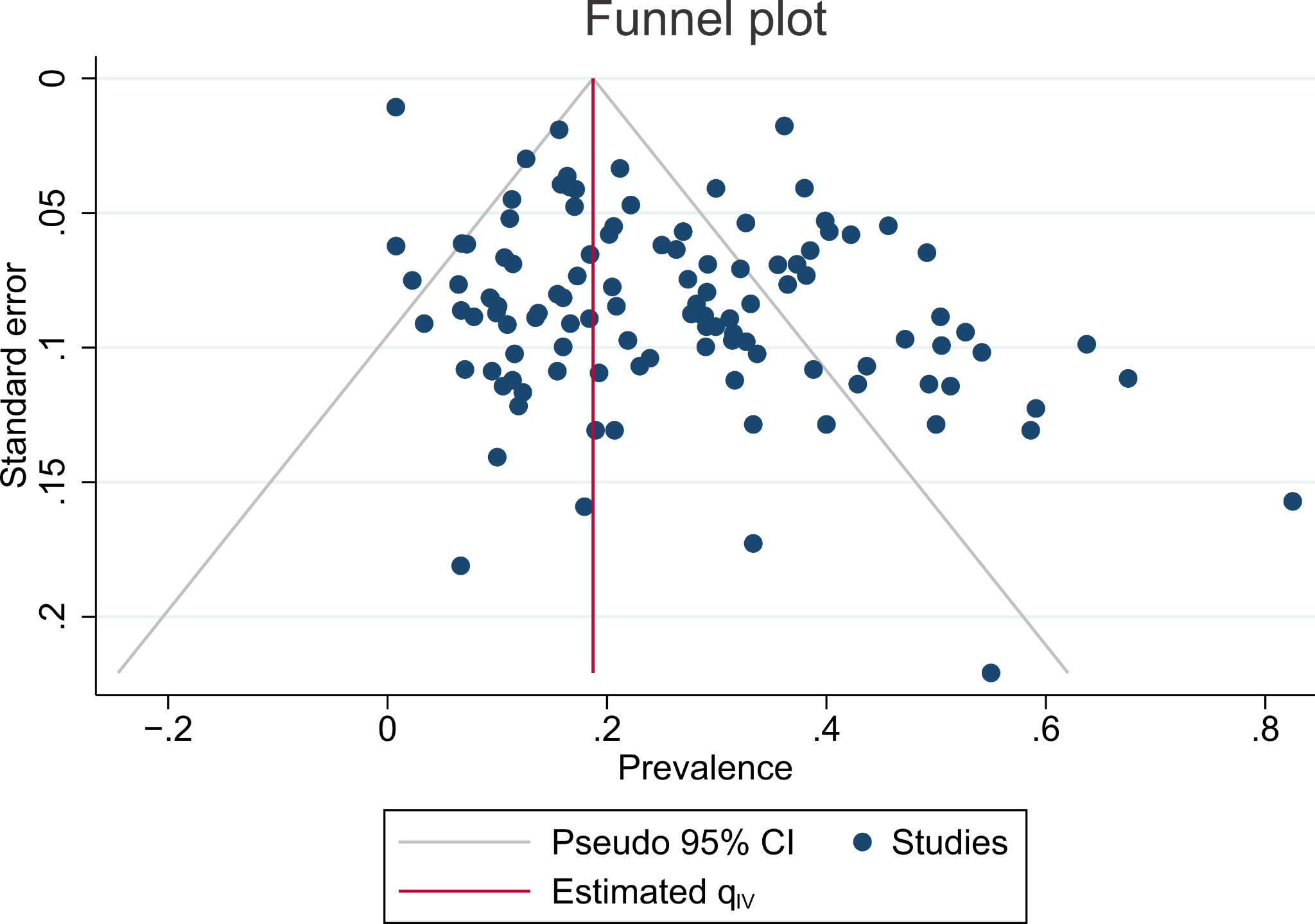


**Supporting Figure 1.** Funnel plot for sarcopenia prevalence in patients with chronic kidney disease.

Begg’s test p=0.174 and Egger’s test p=0.006.


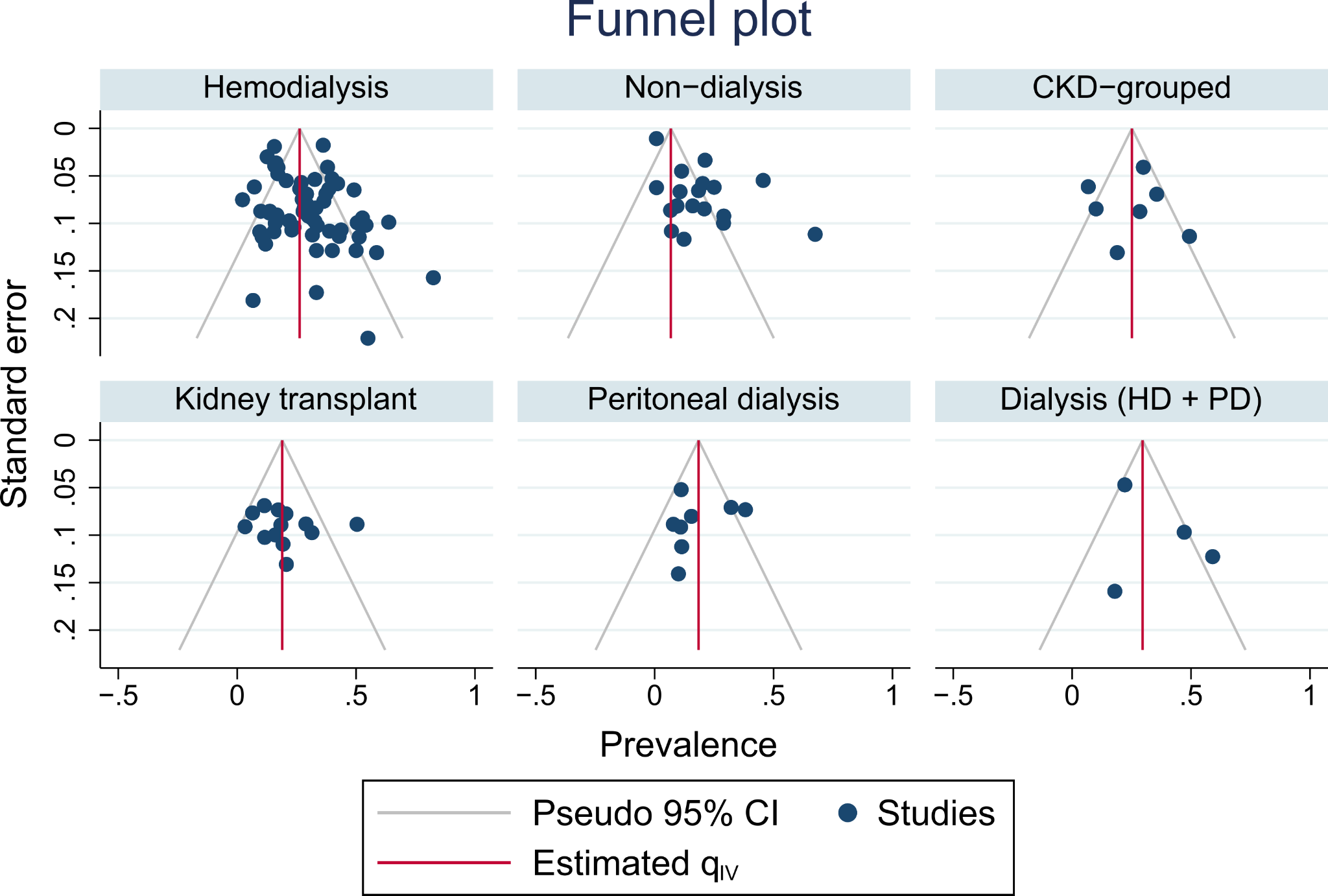


**Supporting Figure 2.** Funnel plots of studies reporting sarcopenia prevalence stratified by CKD subgroups.

P-values according to Egger’s test: p=0.07 for hemodialysis; p=0.20 for non-dialysis; p=0.71 for CKD-grouped; p=0.61 for kidney transplant; p=0.45 for peritoneal dialysis; p=0.78 for dialysis.

**
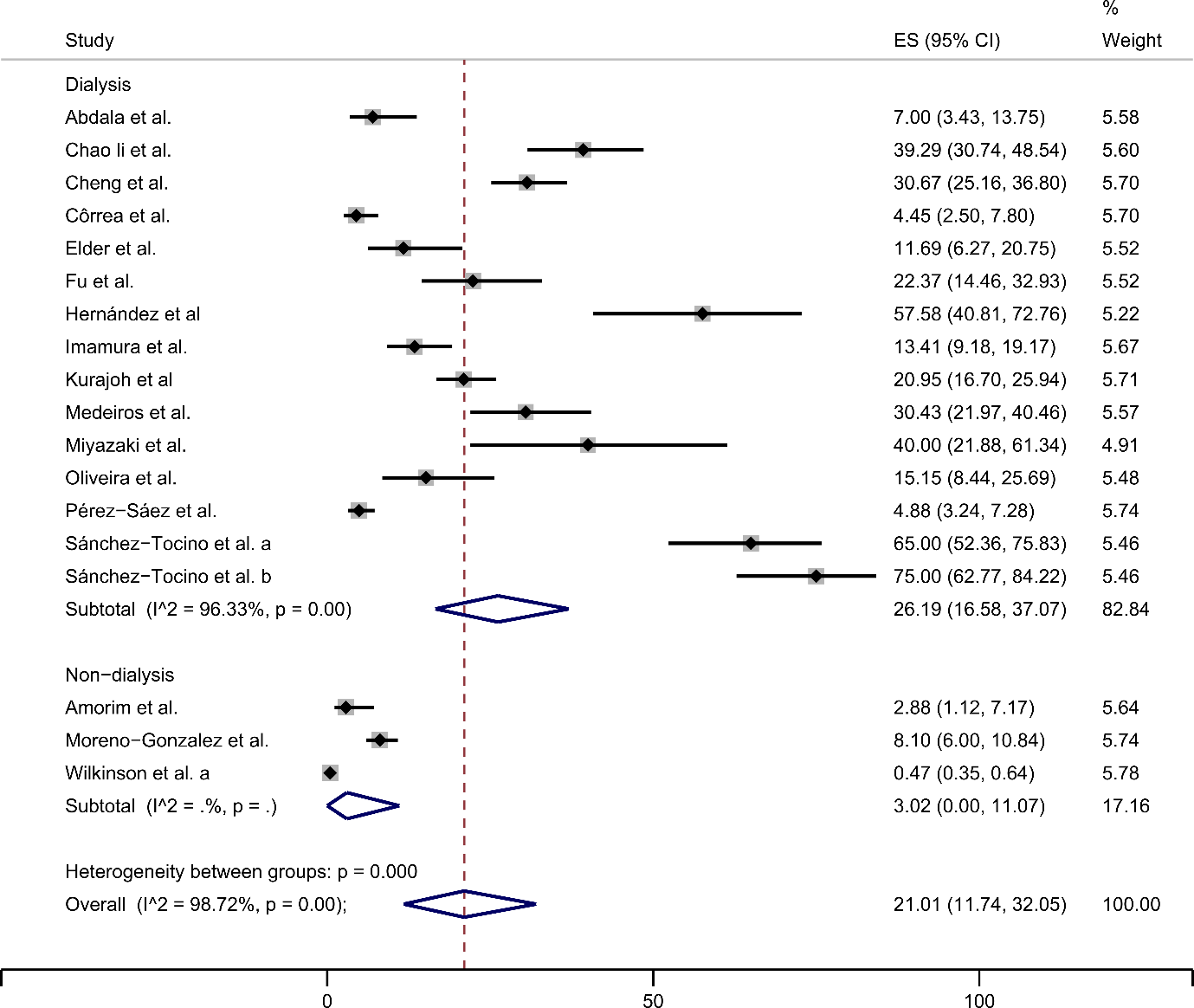
**

**Supporting Figure 3.** Pooled prevalence of severe sarcopenia in patients with chronic kidney disease.

CI, confidence interval.


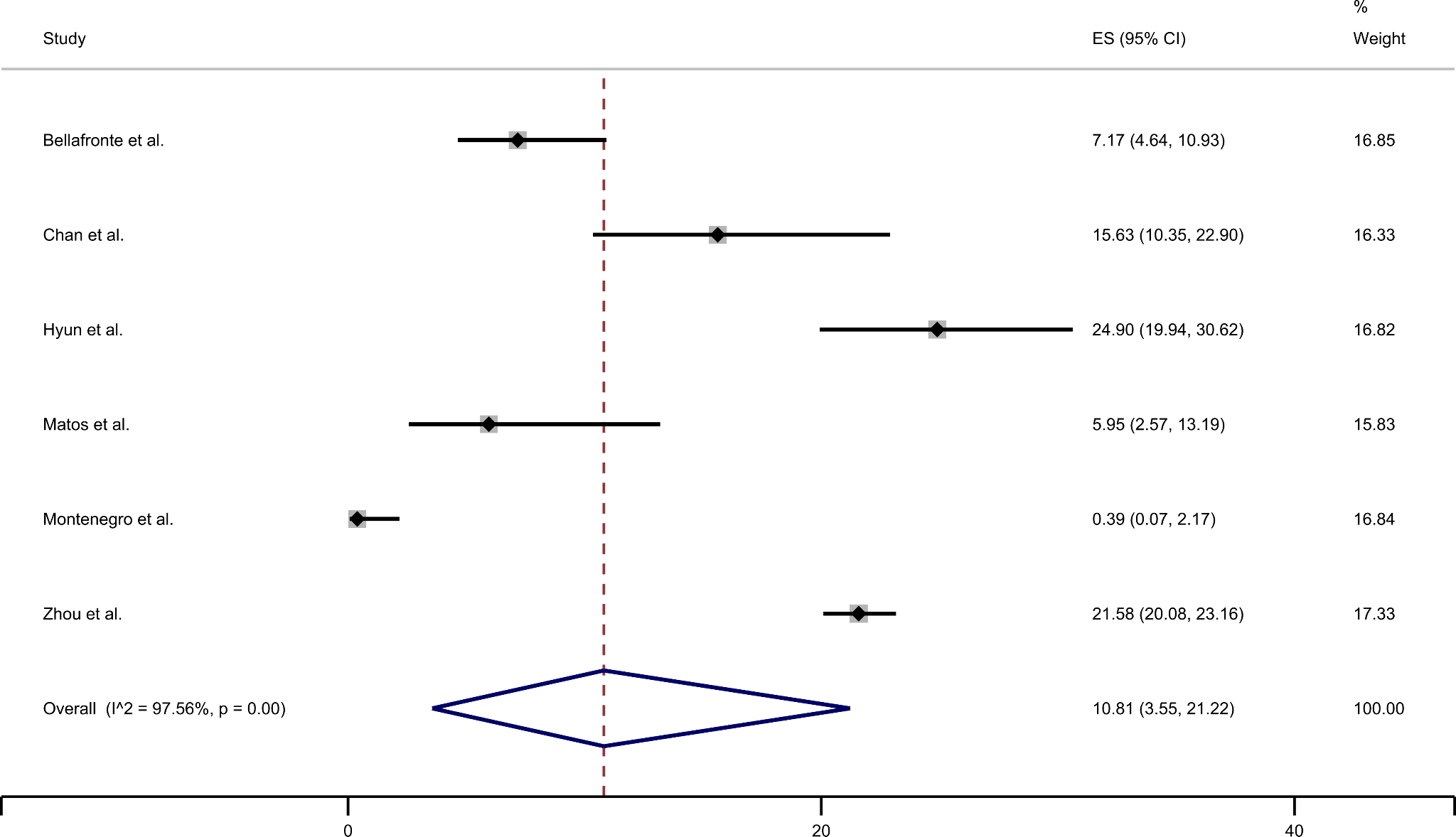


**Supporting Figure 4.** Pooled prevalence of sarcopenic obesity in patients with chronic kidney disease.

CI, confidence interval; CKD; chronic kidney disease.


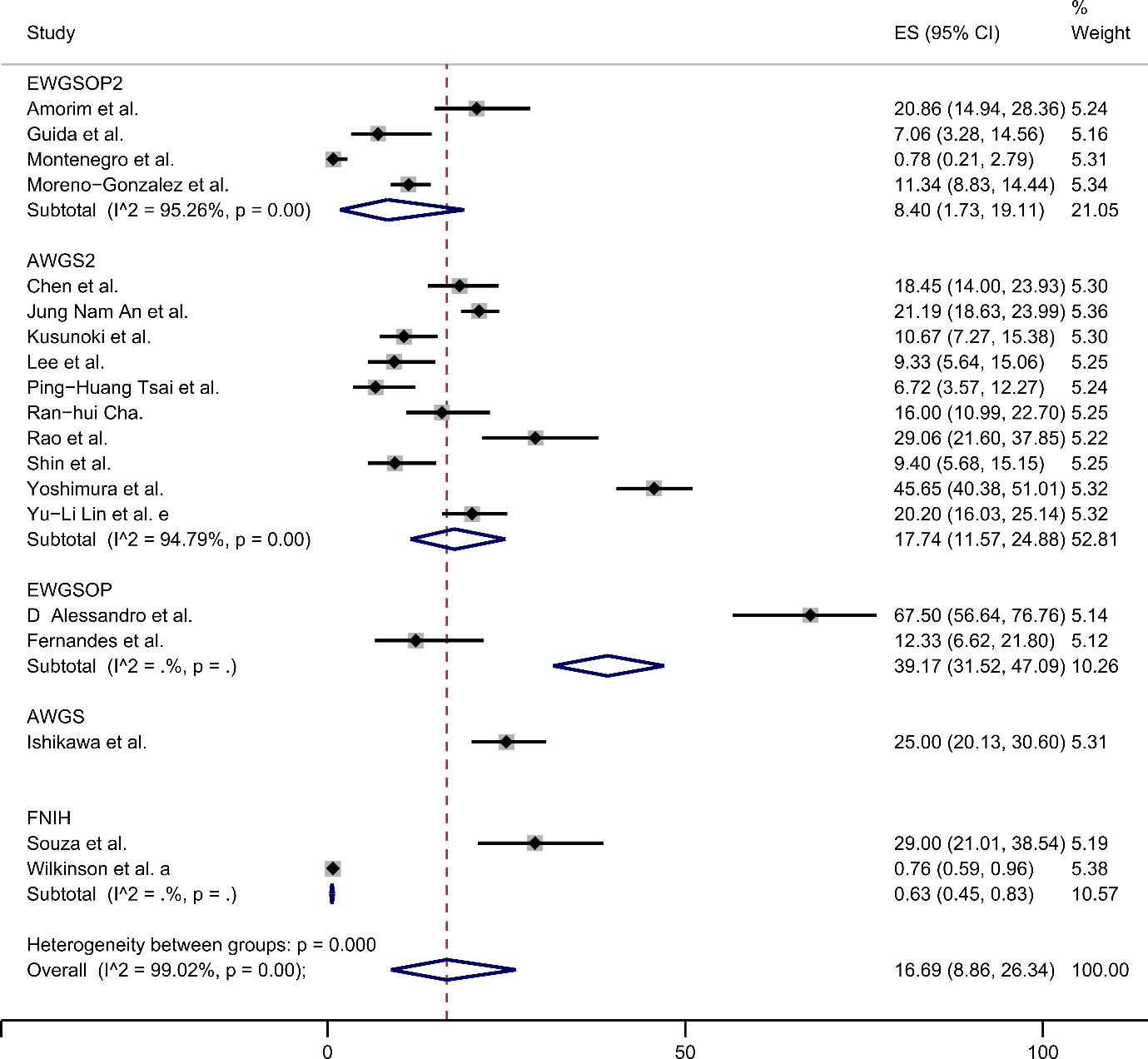


**Supporting Figure 5.** Pooled prevalence of sarcopenia according to diagnosis consensus in non-dialysis patients.

AWGS, Asian Working Group for Sarcopenia; CI, confidence interval; EWGSOP, European Working Group on Sarcopenia in Older People; FNIH; Foundation for the National Institutes of Health Sarcopenia Project.


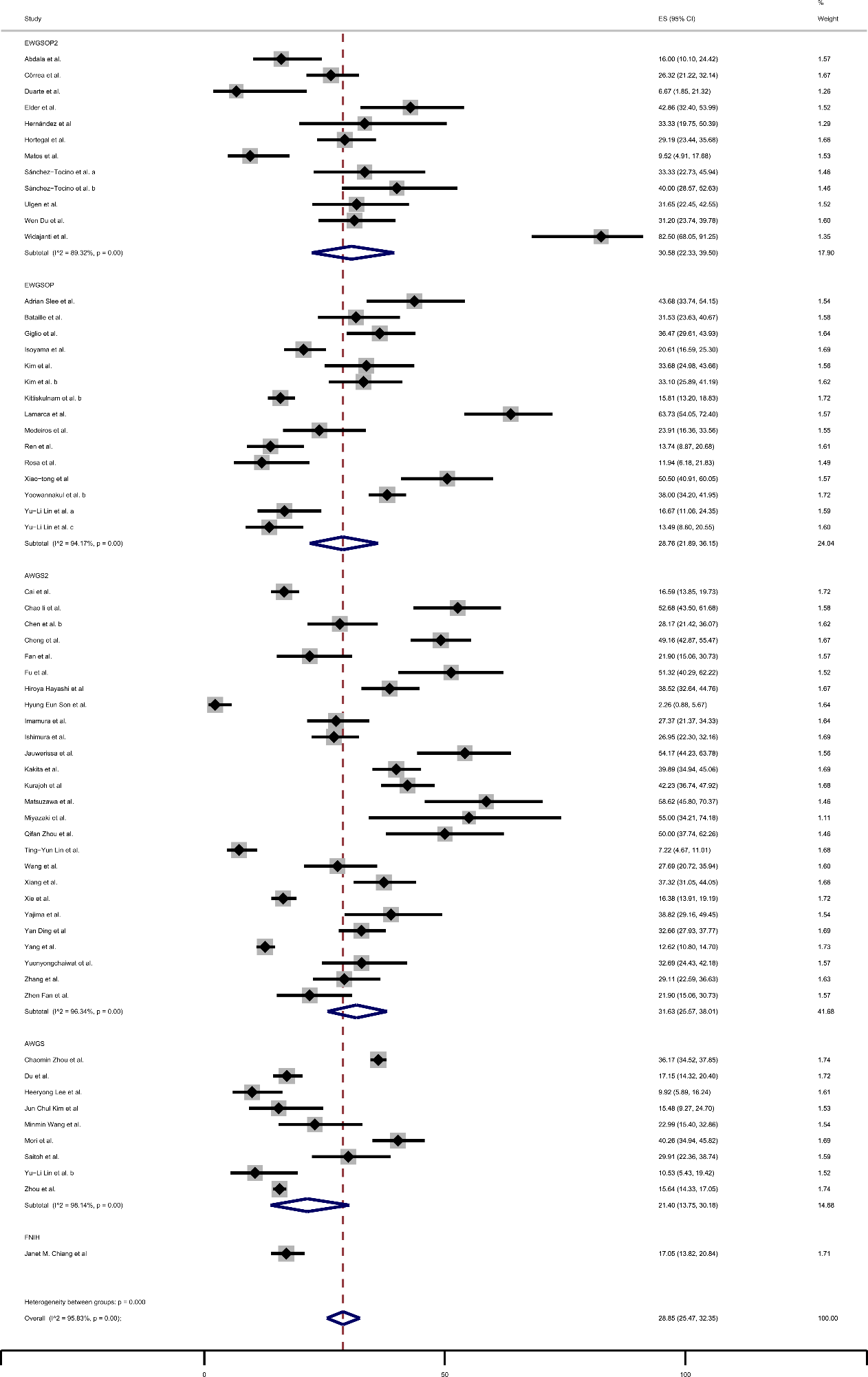


**Supporting Figure 6.** Pooled prevalence of sarcopenia according to diagnosis consensus in hemodialysis patients. AWGS, Asian Working Group for Sarcopenia; CI, confidence interval; EWGSOP, European Working Group on Sarcopenia in Older People; FNIH, Foundation for the National Institutes of Health Sarcopenia.


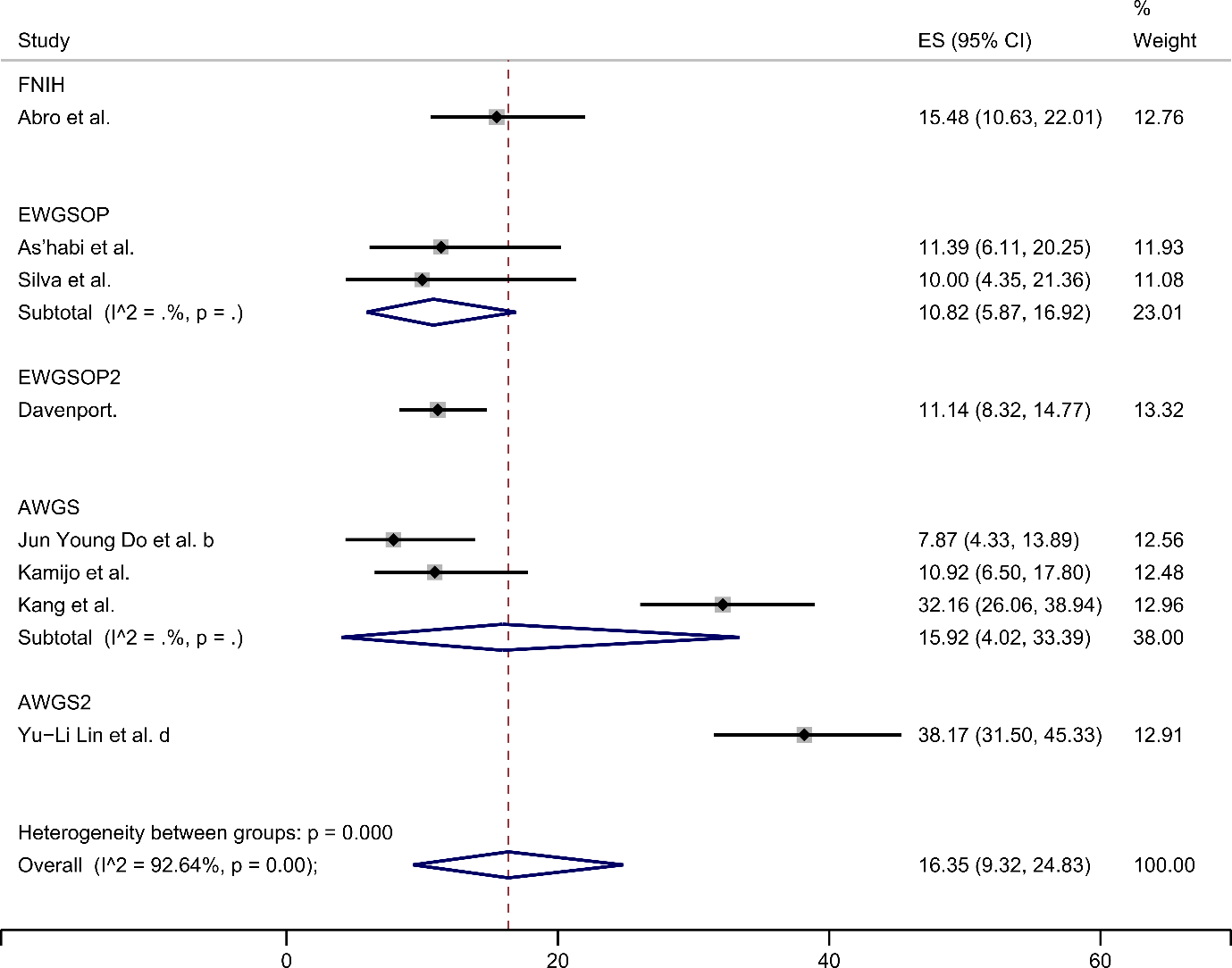


**Supporting Figure 7.** Pooled prevalence of sarcopenia according to diagnosis consensus in patients on peritoneal dialysis. AWGS, Asian Working Group for Sarcopenia; CI, confidence interval; EWGSOP, European Working Group on Sarcopenia in Older People; FNIH; Foundation for the National Institutes of Health Sarcopenia Project.

**Supporting Figure 7.** Pooled prevalence of sarcopenia according to diagnosis consensus in peritoneal dialysis patients. AWGS, Asian Working Group for Sarcopenia; CI, confidence interval; EWGSOP, European Working Group on Sarcopenia in Older People; FNIH; Foundation for the National Institutes of Health Sarcopenia Project.


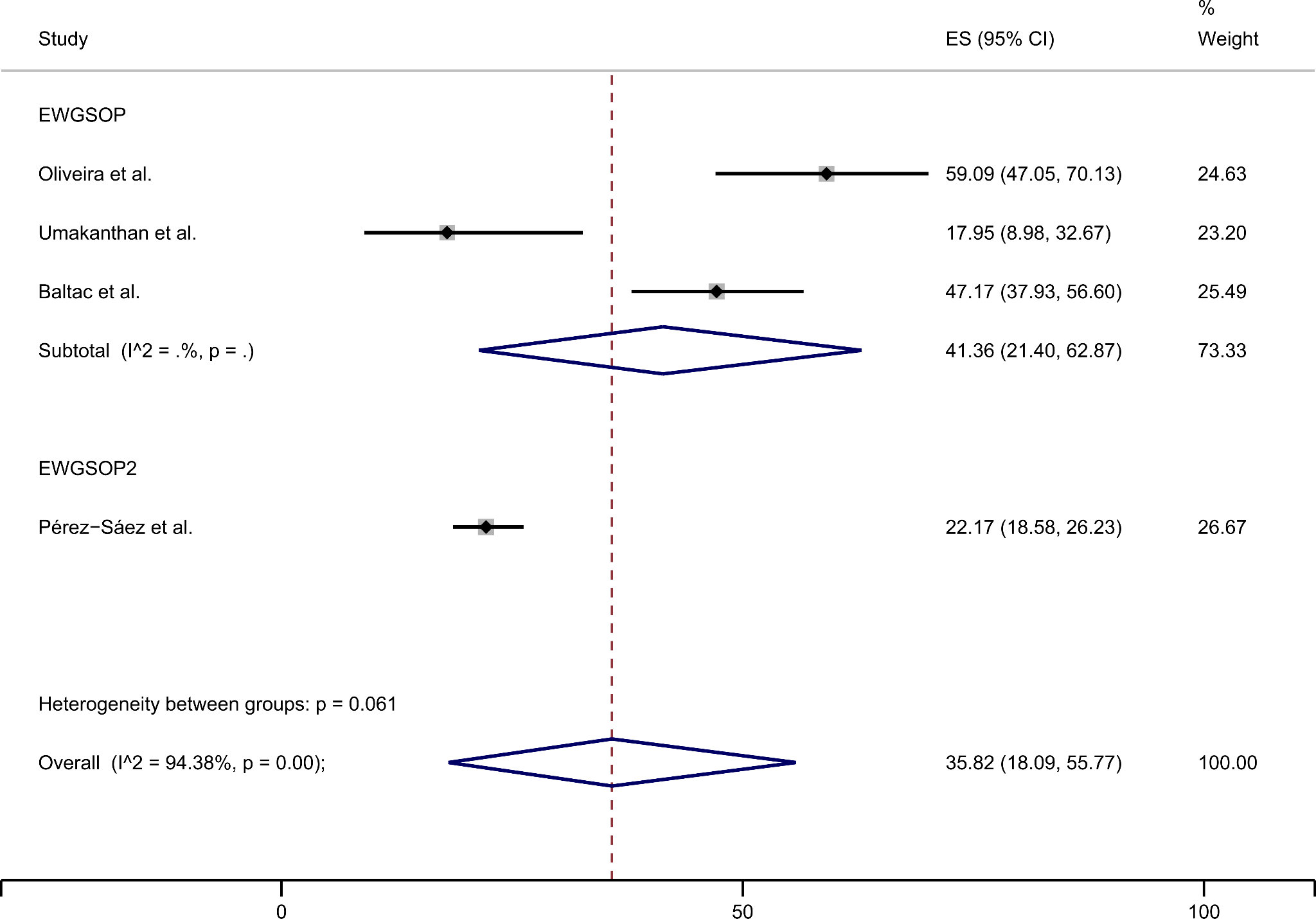


**Supporting Figure 8.** Pooled prevalence of sarcopenia according to diagnosis consensus in dialysis patients (hemodialysis + peritoneal dialysis). AWGS, Asian Working Group for Sarcopenia; CI, confidence interval; EWGSOP, European Working Group on Sarcopenia in Older People.

**Supporting Figure 9.** Pooled prevalence of sarcopenia according to diagnosis consensus in kidney transplant patients. AWGS, Asian Working Group for Sarcopenia; CI, confidence interval; EWGSOP, European Working Group on Sarcopenia in Older People; IWGS, International Working Group on Sarcopenia.

**Supporting Figure 8.** Pooled prevalence of sarcopenia according to diagnosis consensus in patients on dialysis (hemodialysis + peritoneal dialysis).

EWGSOP, European Working Group on Sarcopenia in Older People.
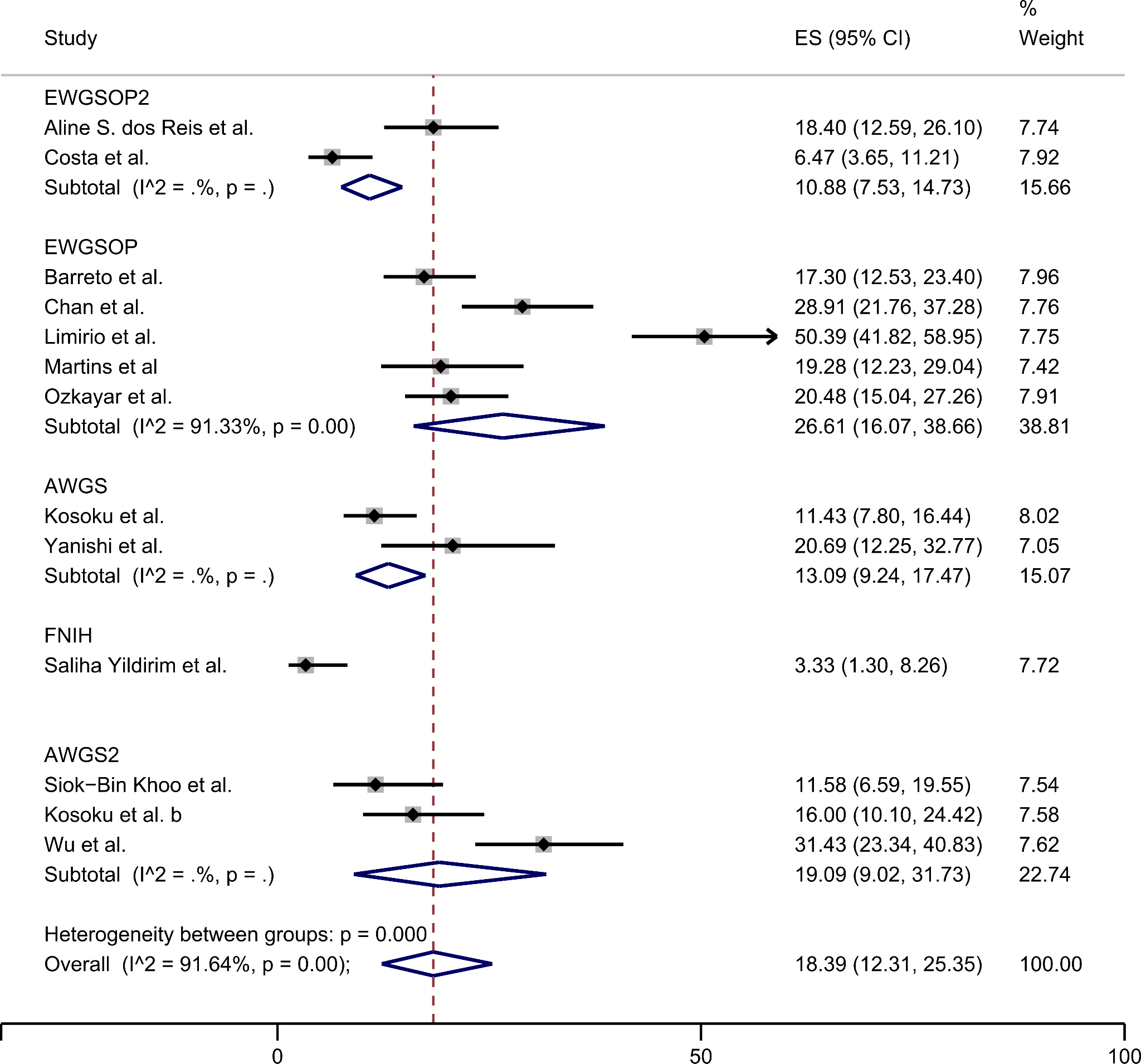


**Supporting Figure 9.** Pooled prevalence of sarcopenia according to diagnosis consensus in kidney transplant patients. AWGS, Asian Working Group for Sarcopenia; CI, confidence interval; EWGSOP, European Working Group on Sarcopenia in Older People; FNIH; Foundation for the National Institutes of Health Sarcopenia Project.

**Supporting Figure 10.** Pooled prevalence of sarcopenia according to diagnosis consensus in CKD-grouped patients. CI, confidence interval; EWGSOP, European Working Group on Sarcopenia in Older People; FNIH, Foundation for the National Institutes of Healt Sarcopenia Project.


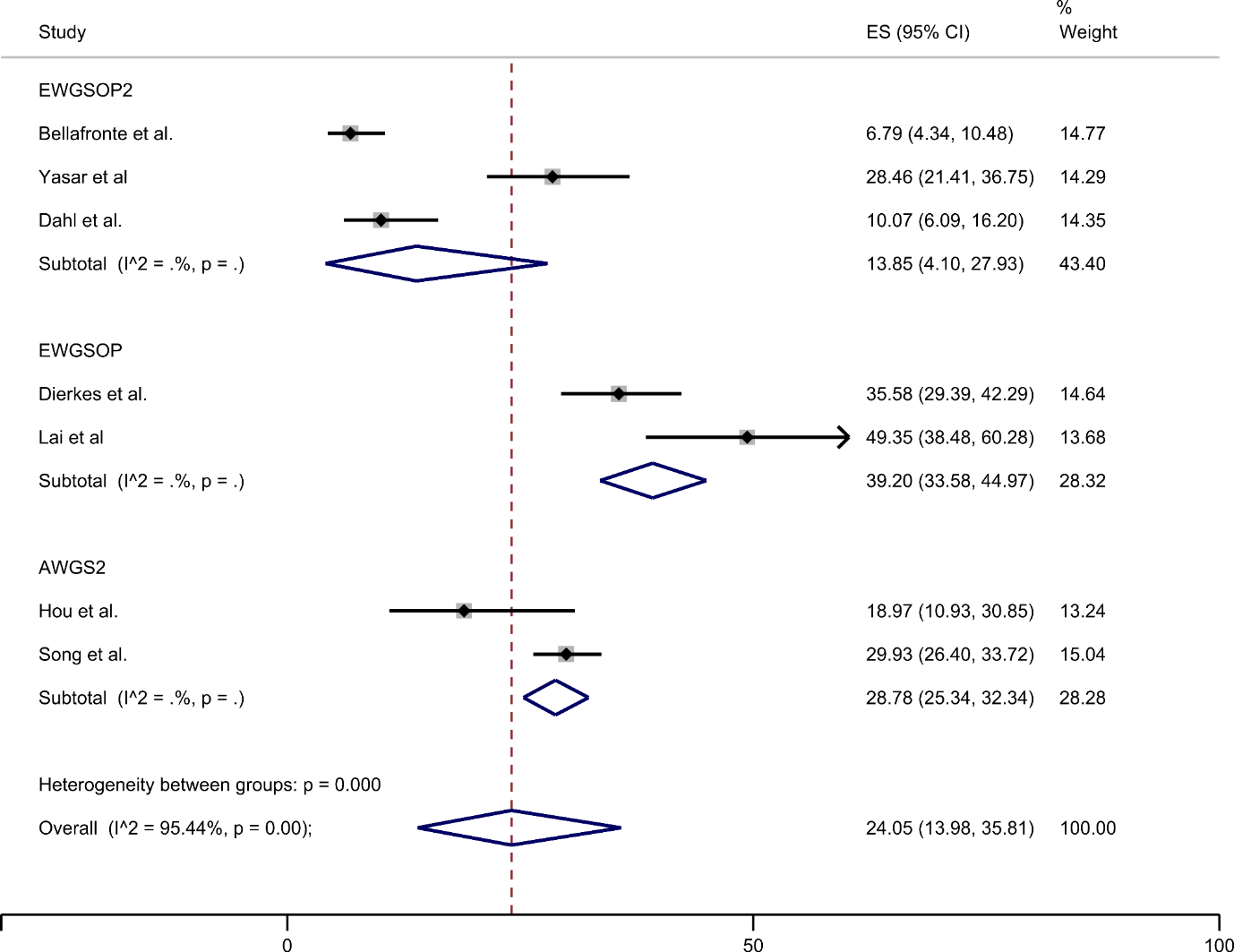
**Supporting Figure 10.** Pooled prevalence of sarcopenia according to diagnosis consensus in CKD-grouped patients.

AWGS, Asian Working Group for Sarcopenia; CI, confidence interval; CI, confidence interval; EWGSOP, European Working Group on Sarcopenia in Older People.


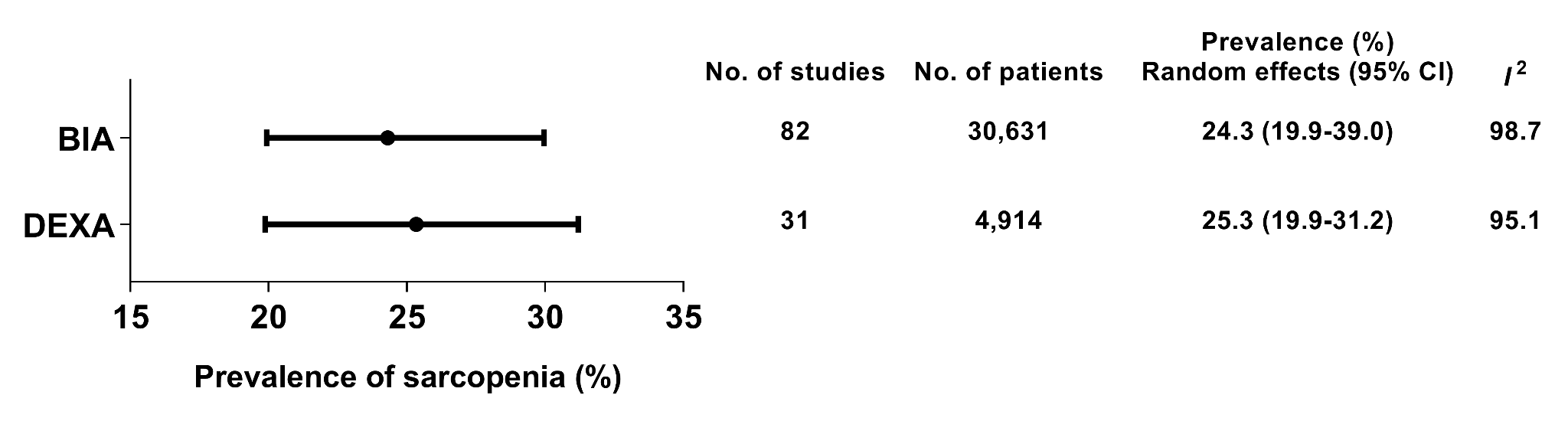


**Supporting Figure 11.** Pooled prevalence of sarcopenia according to device mass tool.

P-value=0.710; overall heterogeneity: I2=98.4%; n=113 studies were included in this analysis.


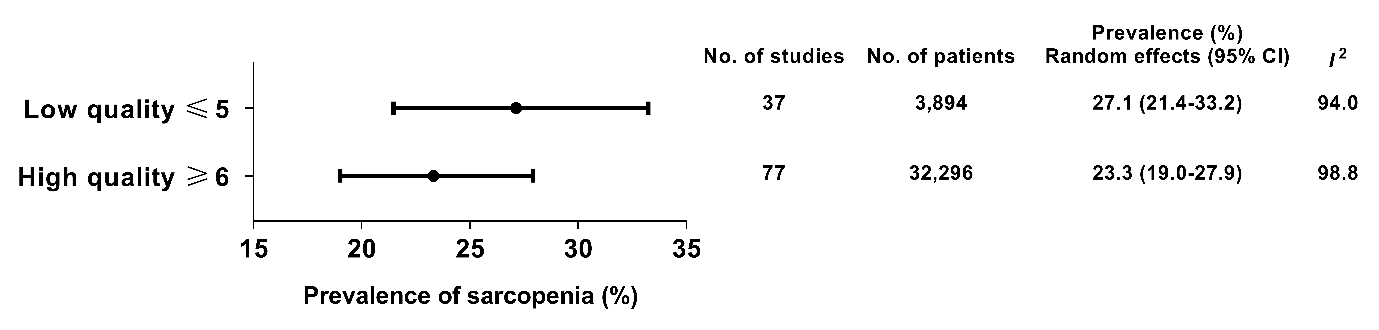


**Supporting Figure 12.** Pooled prevalence of sarcopenia according to methodological quality.

P-value=0.139; overall heterogeneity: I2=98.4%; n=114 studies were included in this analysis.


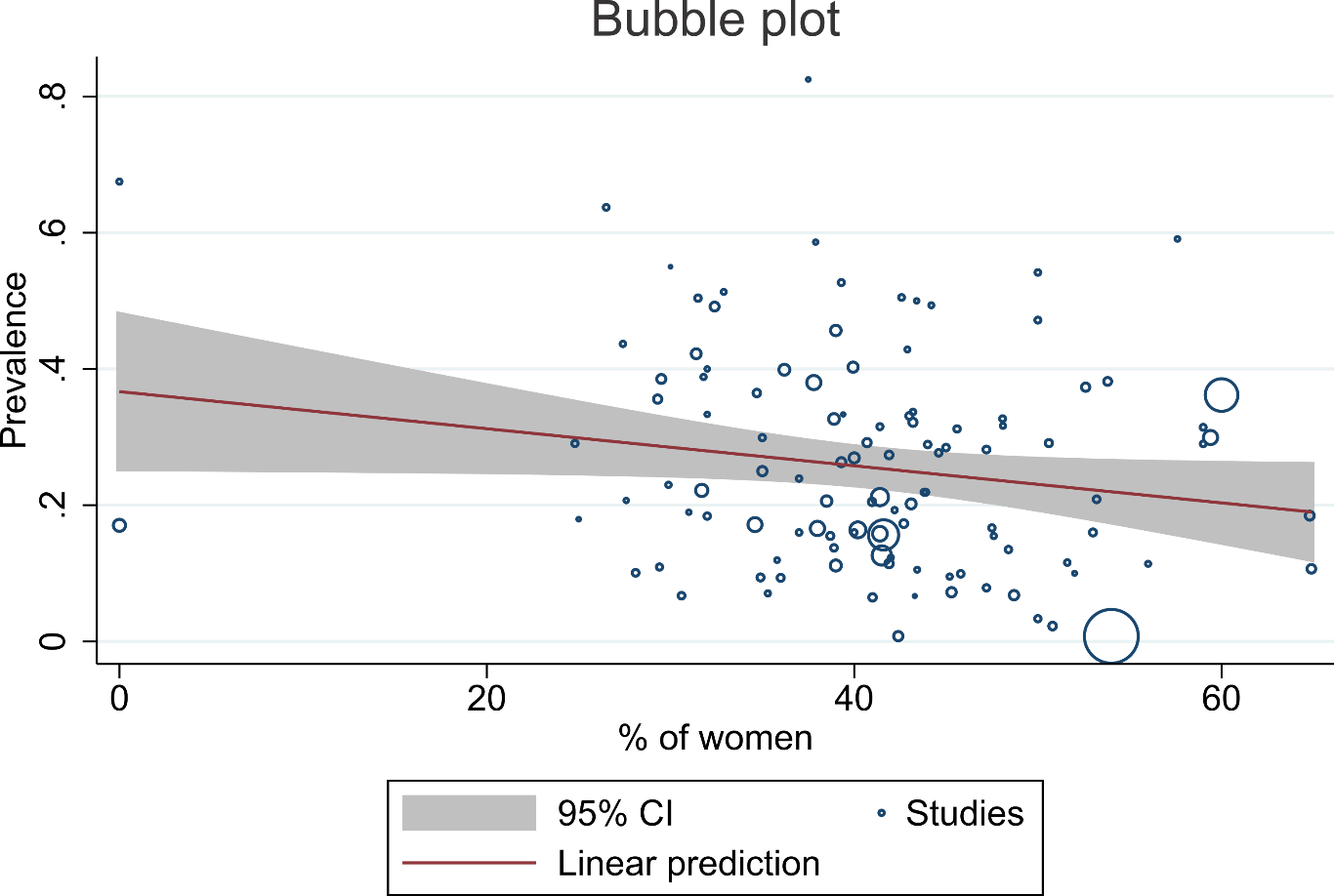


**Supporting Figure 13.** Bubble plot of sarcopenia prevalence by the percentage of women in the study sample.

P-value=0.0446; residual heterogeneity: I2=79.9%; n=113 studies were included in this analysis.


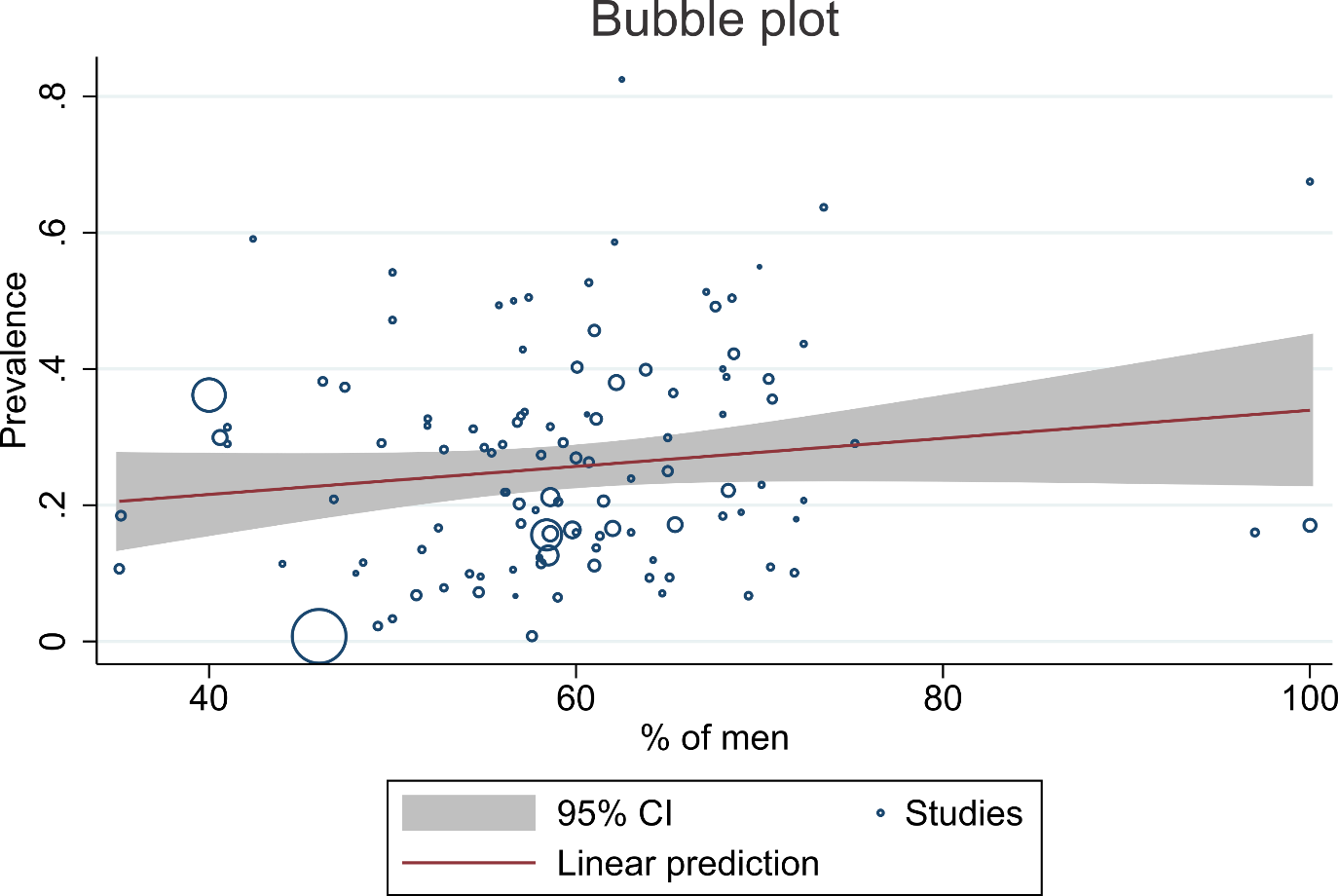


**Supporting Figure 14.** Bubble plot of sarcopenia prevalence by the percentage of men in the study sample.

P-value=0.105; residual heterogeneity: I2=80.2%; n=113 studies were included in this analysis.


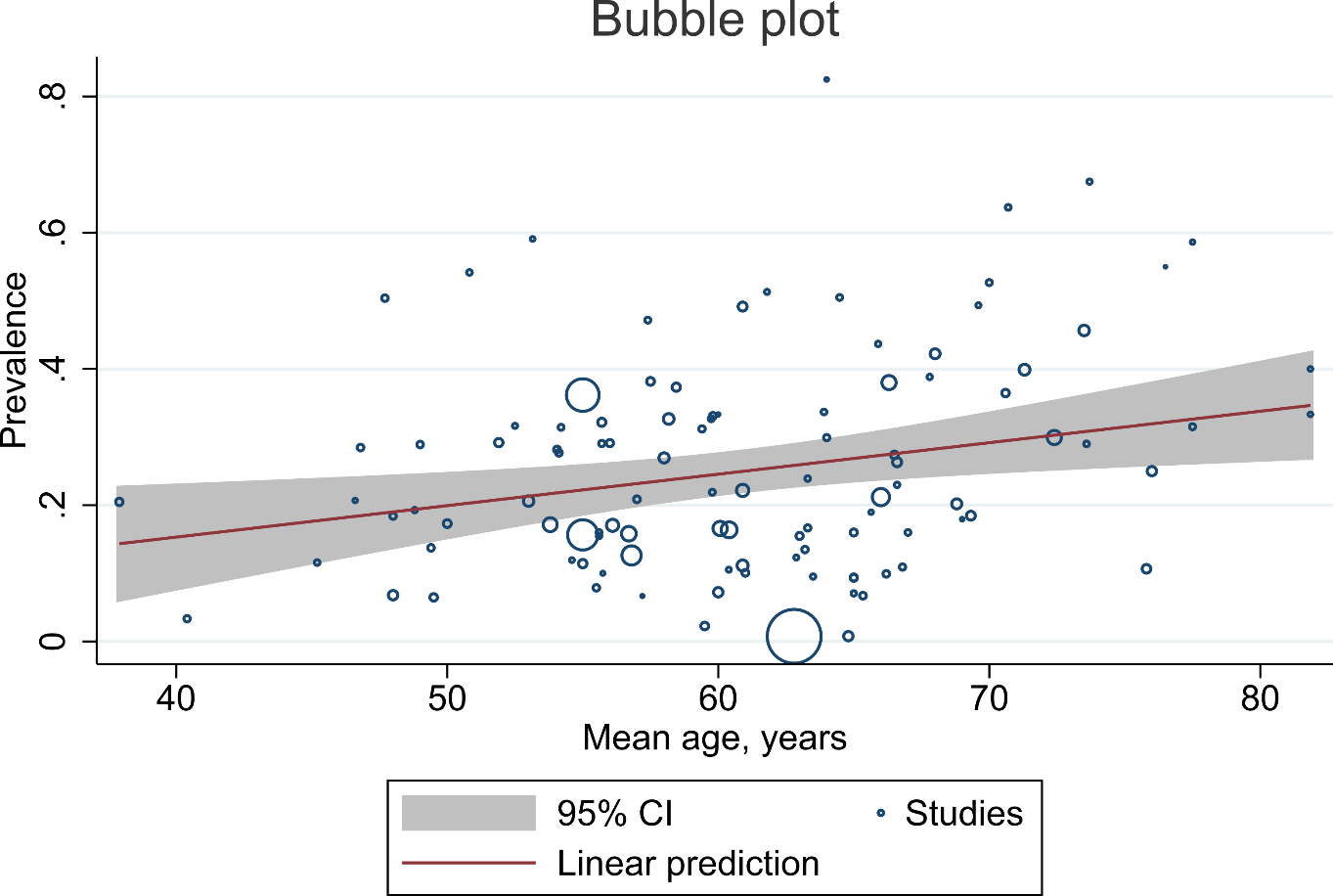


**Supporting Figure 15.** Bubble plot of sarcopenia prevalence by the mean age of patients.

P-value=0.0064; residual heterogeneity: I2=80.3%; n=106 studies were included in this analysis.


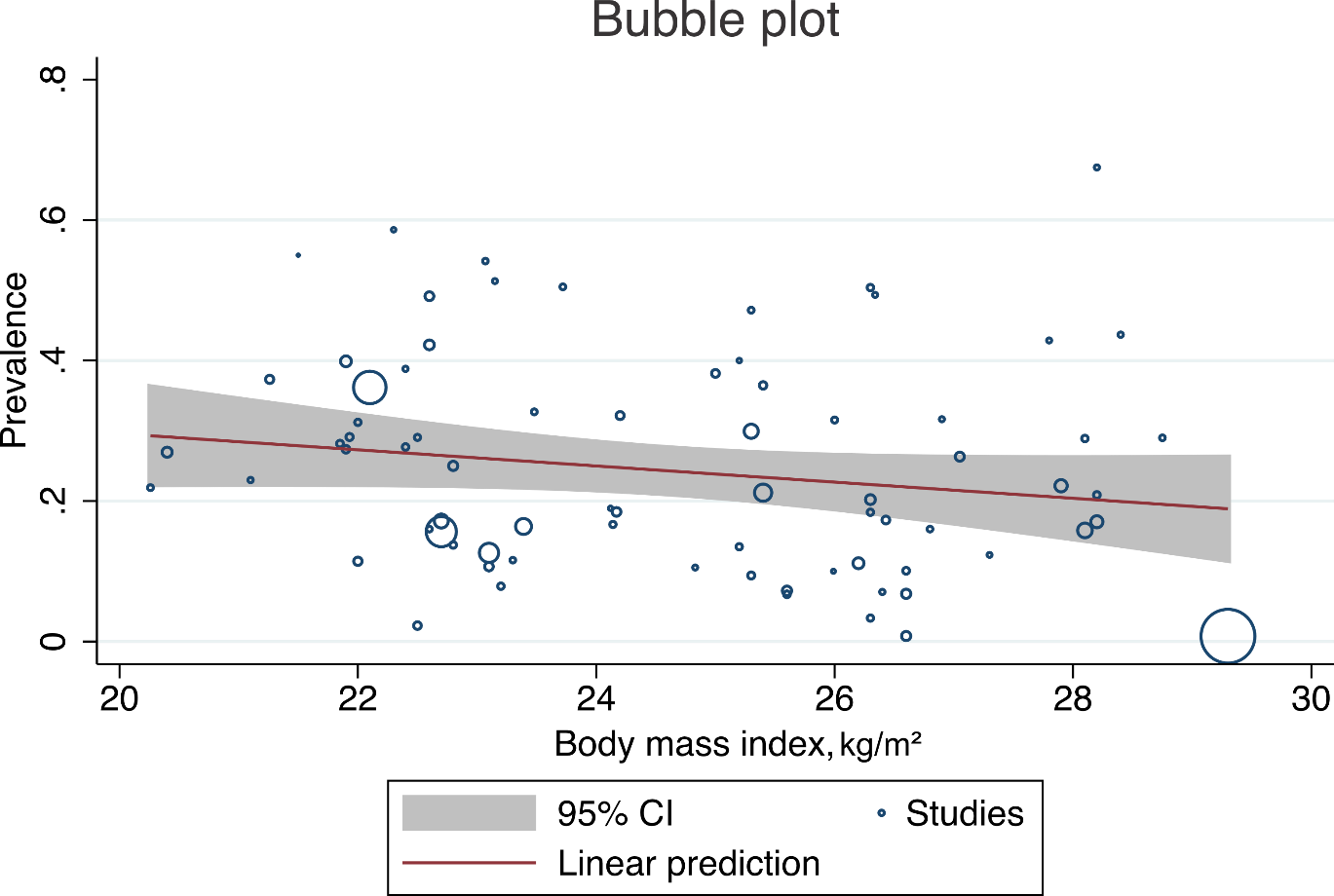


**Supporting Figure 16.** Bubble plot of sarcopenia prevalence by the body mass index of patients.

P-value=0.107; residual heterogeneity: I2=80.1%; n=75 studies were included in this analysis.


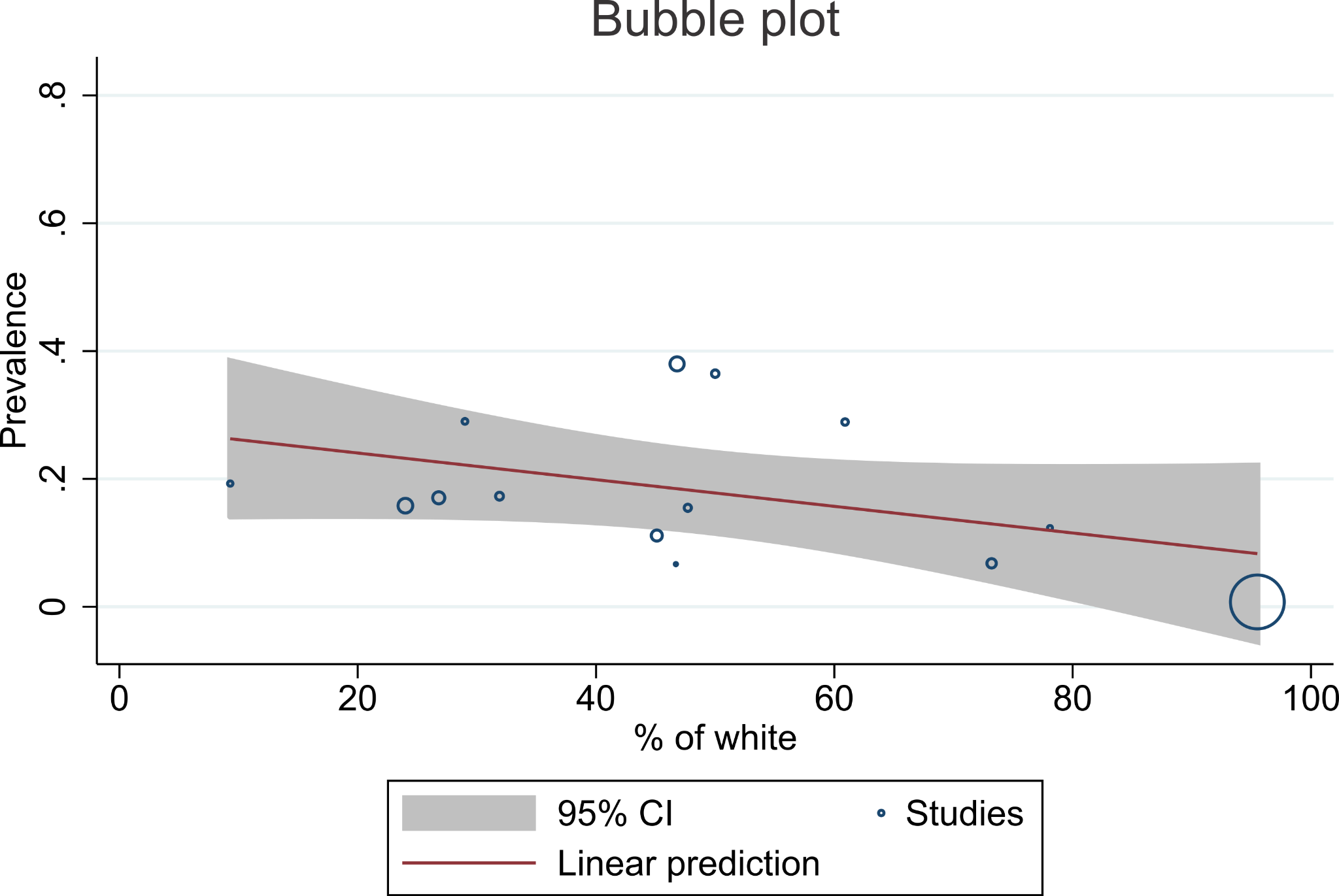


**Supporting Figure 17.** Bubble plot of sarcopenia prevalence by the percentage of white patients.

P-value=0.120; residual heterogeneity: I2=69.4%; n=14 studies were included in this analysis.


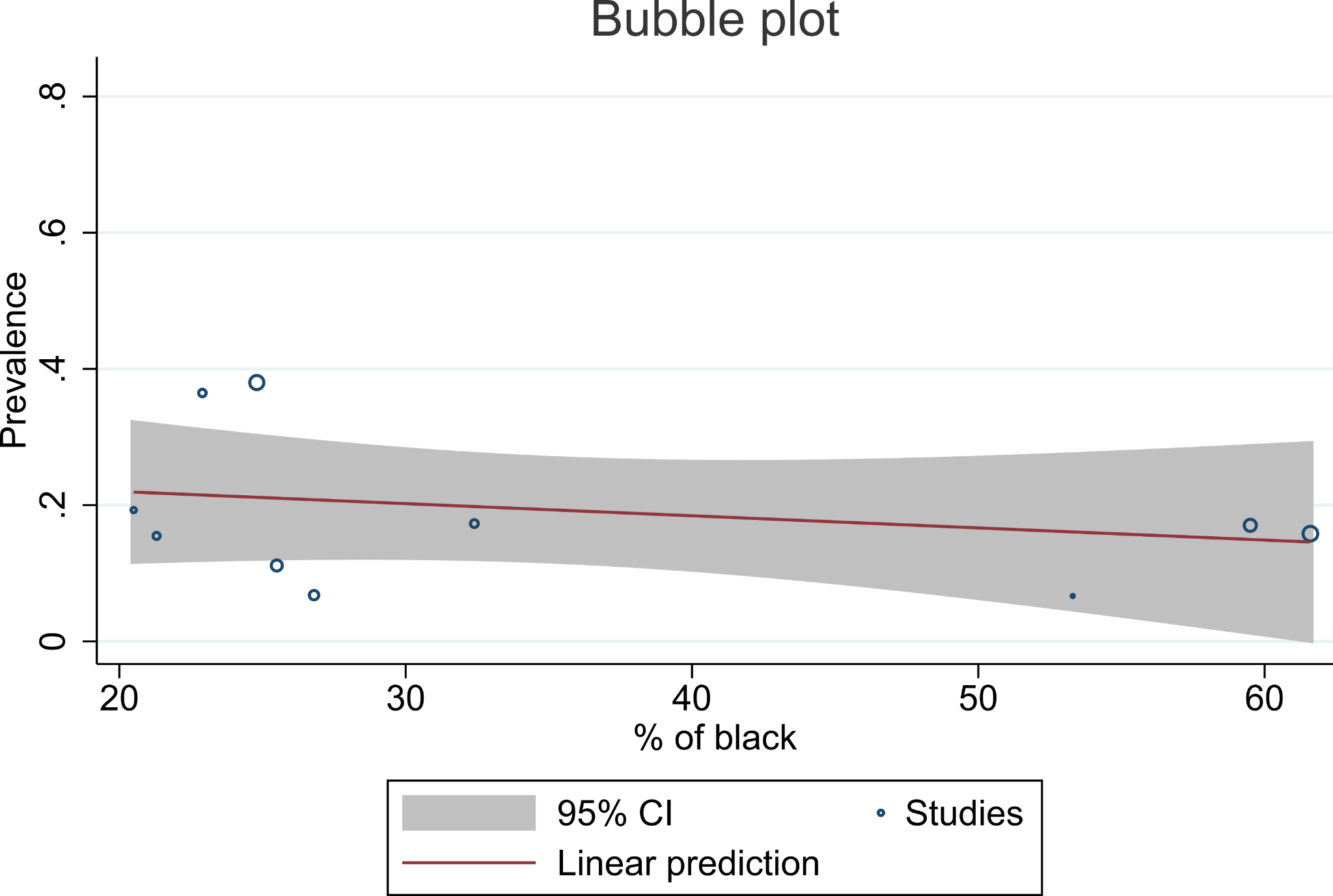


**Supporting Figure 18.** Bubble plot of sarcopenia prevalence by the percentage of black patients.

P-value=0.452; residual heterogeneity: I2=70.4%; n=10 studies were included in this analysis.
